# Childhood maltreatment influences adult brain structure through its effects on immune, metabolic, and psychosocial factors

**DOI:** 10.1073/pnas.2304704121

**Published:** 2024-04-09

**Authors:** Sofia C. Orellana, Richard A. I. Bethlehem, Ivan L. Simpson-Kent, Anne-Laura van Harmelen, Petra E. Vértes, Edward T. Bullmore

**Affiliations:** ^a^Department of Psychiatry, University of Cambridge, Cambridge CB2 0SZ, United Kingdom; ^b^Department of Psychology, University of Cambridge, Cambridge CB2 3EB, United Kingdom; ^c^Institute of Psychology, Leiden University, Leiden 2333 AK, The Netherlands; ^d^Medical Research Council Cognition and Brain Sciences Unit, University of Cambridge, Cambridge CB2 7EF, United Kingdom; ^e^Department of Psychology, University of Pennsylvania, Philadelphia, PA 19104-6241; ^f^Institute of Education and Child Studies, Leiden University, Leiden 2333 AK, The Netherlands; ^g^Cambridgeshire & Peterborough NHS Foundation Trust, Cambridge CB21 5EF, United Kingdom

**Keywords:** childhood maltreatment, metabolic, inflammation, gray matter, MRI

## Abstract

Childhood maltreatment (CM) leads to a lifelong susceptibility to mental ill-health which might be reflected by its effects on adult brain structure, perhaps indirectly mediated by its effects on adult metabolic, immune, and psychosocial systems. Indexing these systemic factors via body mass index (BMI), C-reactive protein (CRP), and rates of adult trauma (AT), respectively, we tested three hypotheses: (H1) CM has direct or indirect effects on adult trauma, BMI, and CRP; (H2) adult trauma, BMI, and CRP are all independently related to adult brain structure; and (H3) childhood maltreatment has indirect effects on adult brain structure mediated in parallel by BMI, CRP, and AT. Using path analysis and data from *N* = 116,887 participants in UK Biobank, we find that CM is related to greater BMI and AT levels, and that these two variables mediate CM’s effects on CRP [H1]. Regression analyses on the UKB MRI subsample (*N* = 21,738) revealed that greater CRP and BMI were both independently related to a spatially convergent pattern of cortical effects (Spearman’s ρ = 0.87) characterized by fronto-occipital increases and temporo-parietal reductions in thickness. Subcortically, BMI was associated with greater volume, AT with lower volume and CPR with effects in both directions [H2]. Finally, path models indicated that CM has indirect effects in a subset of brain regions mediated through its direct effects on BMI and AT and indirect effects on CRP [H3]. Results provide evidence that childhood maltreatment can influence brain structure decades after exposure by increasing individual risk toward adult trauma, obesity, and inflammation.

## Significance

Childhood maltreatment can make individuals more susceptible to poor health outcomes across the lifespan; however, the mediating pathways between early life adversity and adult obesity, inflammation, social trauma, and brain structure are not yet clear. In a large sample of 20,000+ adults, we show childhood maltreatment was directly predictive of adult trauma, obesity, and inflammation and indirectly predictive of brain structural changes associated with these adult immuno-metabolic or social risk factors. These results highlight some of the paths that may be taken to mediate the long-term, systemic consequences of early life adversity for adult brain structure and health risks.

Childhood maltreatment (CM) is commonly understood to comprise experiences of emotional, physical, and sexual abuse or emotional and physical neglect, in the first 15 y of life (1). Individuals affected by CM have an increased lifetime risk of mental ill-health (2) representing approximately 30% of psychiatric patients worldwide (3), and characterized clinically by an increased recurrence, earlier onset, and greater severity of mental health symptoms (2, 4). This lifelong vulnerability to mental ill-health is likely conferred by structural brain alterations commonly present in individuals exposed to early adversity (5). Here, we aim to shed light on the mediating pathways that link exposure to childhood maltreatment to alterations in brain structure many decades later.

It is well established that early adversity has several effects within the highly interrelated domains of immune, metabolic, and psychosocial function. In both prospective human studies and animal models, childhood maltreatment precedes the onset of low-grade chronic inflammation in the periphery, even in the absence of infection (6, 7). Blood inflammatory status has typically been indexed by serum concentration of proinflammatory cytokines and acute phase proteins, such as C-reactive protein (CRP), which are molecular markers of activation of the innate immune system (8, 9). Increased blood cytokine and CRP levels have been shown to predict the onset of psychopathology across several disorders (10, 11) and are characteristically increased in patients with a history of childhood adversity (12, 13).

Metabolically, early life maltreatment is linked to a higher risk of obesity and its associated disorders, such as diabetes and cardiovascular disease (14, 15). Obesity is a chronic proinflammatory factor, (16) as adipose tissue includes many innate immune cells which can release proinflammatory cytokines into the circulation (17). Psychosocially, childhood maltreatment is strongly predictive of revictimization in the form of adult traumatic experiences involving interpersonal conflict, assault, or material precariousness (18, 19). Physiological responses to repeated social stress include chronic activation of the hypothalamic–pituitary–adrenal (HPA) axis and compensatory downregulation of anti-inflammatory signaling mediated via the glucocorticoid receptor (GR) (20, 21). Hypothetically, therefore, long-term metabolic and social effects of CM could contribute to increased inflammation in adult survivors of early adversity.

Alterations in brain structure have also been associated with inflammation, obesity, and adult trauma. Global reductions in brain volume and decreased cortical thickness in the insula and prefrontal cortex have been linked to increased blood CRP levels (22, 23), and increased CRP has also been associated with cerebral atrophy in diverse neurodegenerative disorders (24). Greater body mass index (BMI) is related to decreased global brain volume (25), and visceral fat content has a U-shaped relationship with cortical thickness across all lobes (26), in line with reports of widespread decreases in region-specific cortical thickness associated with higher BMI (27). Adult trauma exposure has been associated with hippocampal volume reduction (28, 29) as well as changes in thickness of prefrontal and temporo-parietal cortical areas (30, 31). Consequently, it is possible that early adversity has long-term influences on brain structure through its preceding impact on immune, metabolic, nervous, and psychosocial systems.

We therefore sought to shed light on the long-term consequences of childhood maltreatment on psychophysiological systems and their interactions. We reasoned that there could be a chain of predictive relationships linking exposure to childhood maltreatment to alterations in adult brain structure via the intermediate effects of childhood maltreatment on risks for obesity, inflammation, and adult trauma. On this basis, we tested the following hypotheses: (H1) childhood maltreatment has indirect effects on CRP through its direct effects on BMI and adult trauma; (H2) adult trauma, BMI, and CRP are all independently related to adult brain structure; and (H3) effects of CM on adult brain structure are mediated by its parallel effects on adult trauma, BMI, and CRP.

## Results

### Sample.

We test all hypotheses on the *N* = 21,738 participants (UKB MRI sample) who had complete, quality-controlled T1-weighted and T2-weighted whole brain MRI data, as well as data on adult trauma, BMI, and CRP available. We additionally replicate H1 on a larger sample (*N* = 116,887) of participants who met the relevant inclusion criteria for this study but lacked imaging data (Table 1). The UK Biobank cohort is wealthier and healthier than the general population (32), and accordingly, rates of CM and AT are lower in this sample (*SI Appendix*, 2.1).

**Table 1.** Demographic and clinical data on UKB sample and subsample with MRI data available

	UKB MRI subsample	95% C	UKB sample	95% CI
*N*	*N* = 21,738	100%, 100%	*N* = 116,887	100%, 100%
Female n(%)	11,684 (54%)	–	65,715 (56%)	–
SES	−1.94 (2.69)	−2.0, − 1.9	−1.68 (2.85)	−1.7, −1.7
Age at baseline	55 (7)	55, 55	56 (8)	56, 56
Age at MRI	64 (7)	64, 64	–	–
Body Mass Index (kg/m^2^)	26.5 (4.2)	26, 27	26.8 (4.6)	27, 27
CRP, mg/L	2.03 (3.53)	2.0, 2.1	2.29 (4.02)	2.3, 2.3
Childhood Maltreatment	1.70 (2.31)	1.7, 1.7	1.76 (2.41)	1.7, 1.8
Adult Trauma	1.93 (2.37)	1.9, 2.0	2.09 (2.51)	2.1, 2.1

Mean and SD (in brackets) are reported for each variable, unless otherwise specified. All values displayed are raw; for log-transformed summary stats, see *SI Appendix*, Table S4.

### Relationships between Childhood Maltreatment, Adult Trauma, BMI, and CRP.

**Fig. 1.**
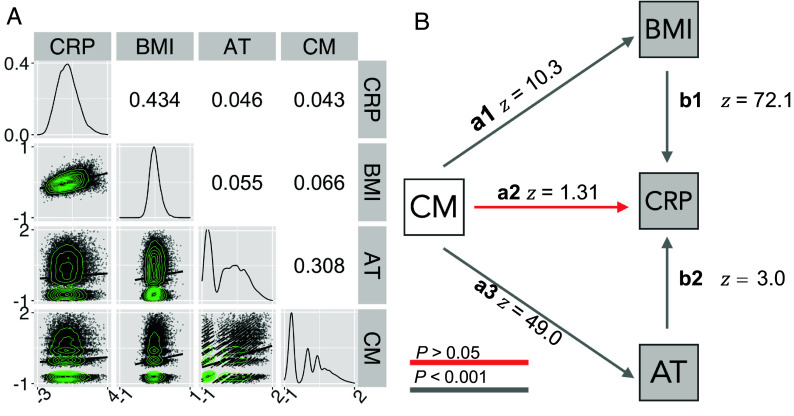
Relationships between childhood maltreatment, adult trauma, BMI, and CRP. (*A*) Correlation matrix representing pair-wise Spearman’s correlations (*Upper* triangle) and scatterplots of the relationships between each pair of variables, with solid lines indicating fitted linear regression models (*Lower* triangle). The diagonal represents the probability density function for each variable. All correlations were significantly greater than zero, with FDR ≤0.05. (*B*) Path diagram representing direct effects of retrospectively ascertained CM (white) on the contemporaneously measured adult (log-transformed) variables, AT, CRP, and BMI (gray). Standardized path coefficients are given as Wald (*z*) statistics.

We used path analysis to examine the magnitude of hypothetically directed relationships between these variables as defined by H1: Childhood maltreatment has effects on adult inflammation (CRP) mediated directly or indirectly by its direct effects on BMI and AT. The correlation matrix demonstrated small-to-moderate positive correlations between each pair of the four model variables (Fig. 1*A*). The strongest correlations were between CRP and BMI (r=0.43), and between CM and AT (r=0.31). All correlations were statistically significant, even when smaller, reflecting the large sample size.

The model fit to this correlation matrix (Fig. 1*B*) included direct effects of retrospectively assessed childhood maltreatment on all 3 adult variables (denoted CM→BMI (*a1*), CM→AT (*a3*), and CM→CRP (*a2*). In path modeling, direct effects that do not share a dependent variable are estimated as independent regression coefficients (33), meaning paths *a1*, *a3*, and *a2* do not correct for each other. This model also included the direct effects of AT and BMI on contemporaneously measured CRP (denoted BMI→CRP*(*b1) and AT→CRP*(*b2). As CM, BMI, and AT all share the same dependent variable (CRP), the paths pointing toward CRP are estimated as partial regression coefficients (33), therefore paths *b1*, *b2*, and *a1* all correct for each other. We additionally also estimated the two possible indirect effects of CM on CRP through the paths CM→BMI→CRP (a1*b1) and CM→AT→CRP (a3*b2). The fit for this model was good: SRMR=0.012;CFI=0.996;RMSEA=0.035.

The estimated path coefficients and their SEs indicated that childhood maltreatment significantly predicted both higher adult BMI (CM→BMI,z=10.343,β=0.072,P<0.001) and greater adult trauma (CM→AT,z=48.882,β=0.315,P<0.001). Note that *z* values represent Wald *Z*’s: *β* coefficients standardized by their standard errors. The direct effects of adult trauma and BMI on CRP were also significant: Higher BMI (BMI→CRP,z=72.047,β=0.434,P<0.001) and higher adult trauma (AT→CRP,z=2.999,β=0.019,P=0.003) were both predictive of higher blood concentration of CRP (Fig. 1*A*). When these contemporaneous effects on CRP were taken into account, the path coefficient representing a direct effect of childhood maltreatment on adult CRP was not significant (CM→CRP,z=1.313,β=0.008,P=0.189) (*SI Appendix*, Table S7). However, indirect effects of childhood maltreatment on CRP, mediated by its direct effects on adult trauma and BMI, were significant: CM→BMI→CRP,z=10.257,β=0,031,P<0.001 (a1*b1); and CM→AT→CRP,z=3.000,β=0.006,P=0.003 (a3*b2) respectively. To test whether the previously reported relationship between CM and CRP (9) disappeared due to the inclusion of indirect effects, we examined the relationship between CM and CRP with a simple linear regression, which yielded significant results (β(SE)=0.03177(0.00470),t=6.757,P<0.0001).

This analysis of data from the UKB MRI sample was repeated using the larger dataset available on the UKB sample (*SI Appendix*, Table 8). In this case, the direct effect of CM on CRP was small but statistically significant: CM→CRP,β=0.0095,z=3.431,P<0.001); see *SI Appendix*, 2.2, Fig. S5*B* and Table S8 for details.

### Independent Effects of Adult Trauma, BMI, and CRP on Cortical Thickness and Subcortical Volumes.

**Fig. 2.**
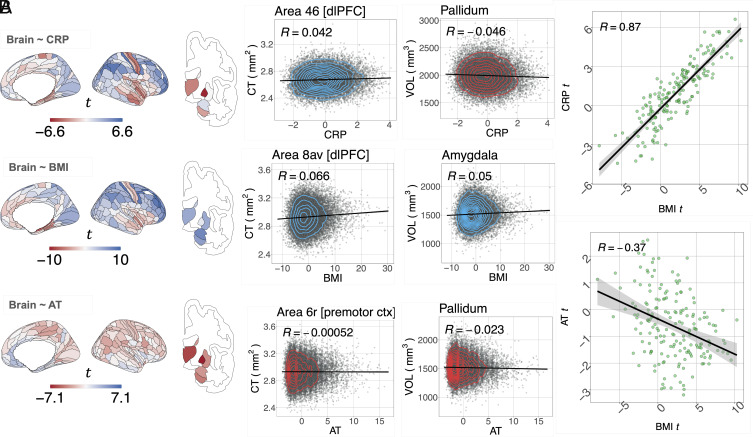
Independent effects of adult trauma, BMI, and CRP on adult cortical thickness and subcortical volumes. (*A, Left column*) Brain maps of independent linear relationships between adult trauma (AT), CRP or BMI, and cortical thickness (CT) and subcortical volume. Each map shows the anatomical distribution of effect sizes (*t*-values); for corresponding maps thresholded for FDR-corrected significance, see *SI Appendix* Fig. S18; for maps of standardized regression *β* coefficients, see SF20A&B. Negative *t*-values indicate a reduction in either cortical thickness or subcortical volume (red), and positive *t*-values indicate an increase in gray matter (blue), as a result of increase in one of these independently treated predictor variables. (*A, Right column*) Illustrative scatterplots of the relationship between each independent variable and the cortical area or subcortical structure most strongly associated with it. Cortical areas are labeled by their specific areal nomenclature and corresponding regional grouping, as defined by the Glasser template (35). (*B, Top panel*) Scatterplot of the effect size (*t*-value) of BMI (*x*-axis) versus the effect size of CRP (*t*-value; *y*-axis) on cortical thickness; each point represents one of 180 cortical areas; Spearman’s correlation $\rho =0.87,P_{\mathrm{spin}}<0.0001$ over all areas; the solid line is the regression of *t*-values for the effect of BMI on *t*-values for the effect of CRP. (*B, Bottom panel*) Scatterplot of the effect size of BMI on CT (*x*-axis) versus the effect size of AT on CT (*y*-axis); Spearman’s ρ=−0.41, $P_{\mathrm{spin}}=0.0009$.

Next, we examine whether BMI, CRP, and AT each have the potential to act as intermediate effectors of CM’s effect on brain structure by determining whether these variables have any direct effects on brain structure, i.e., we tested (H2): Adult trauma, BMI, and CRP are all independently related to adult brain structure. We did this by creating three different linear regression models of the form (Brain ∼ BMI), (Brain ∼ CRP), (Brain ∼ AT) where BMI, CRP, and AT are treated separately as predictors and cortical thickness (or subcortical volume) as the dependent variable at each of 180 cortical areas (or 7 subcortical structures) (Fig. 2). These models included age, sex, socioeconomic status, and the interaction between age and sex as covariates, and they did not correct for each other.

At the cortical level, BMI and CRP displayed a very similar anatomical pattern of cortical thickness variation. The unthresholded maps of linear regression coefficients of BMI and CRP effects on cortical thickness were highly correlated across all cortical areas and robust to spatial autocorrelation as controlled with a spin test (Spearman’s ρ=0.87, $P_{\mathrm{spin}}<0.0001$); Fig. 2*B*. When the effects of CRP and BMI were separately tested for statistical significance at each brain region, with FDR ≤0.05, an extensively overlapping set of regions was identified as significantly associated with both CRP and BMI (*SI Appendix*, Fig. S18). This suggests the existence of a third factor influencing cortical brain structure which shares a high degree of variance with both BMI and CRP, possibly the sequelae of immuno-metabolic interactions within the nervous system (34).

Higher BMI was predictive of significantly increased cortical thickness in 92 areas, with the top 10 largest effects in the dorsolateral-prefrontal cortex, primary somatosensory and motor cortices, and premotor and inferior frontal cortices as well as significantly decreased cortical thickness in 16 areas, with the strongest decreases located in medial temporal, insular and frontal opercular, primary somatosensory and motor, anterior cingulate and medial prefrontal, association auditory, and orbito-frontal cortices (*SI Appendix*, Table S9).

Likewise, higher CRP was predictive of significantly increased cortical thickness in 50 areas, with the top 10 largest significant effects located in dorsolateral-prefrontal, early somatosensory and motor, inferior frontal and parietal, as well as premotor and superior parietal cortices. CRP was also predictive of significantly decreased cortical thickness in 17 areas with the top 10 most significant effects located in insular and frontal opercular, primary somatosensory, auditory and motor, association auditory, lateral temporal, and medial temporal cortices.

In contrast, adult trauma was less strongly predictive of cortical thickness. The unthresholded map of coefficients for the linear regression of AT on cortical thickness was significantly correlated with the map for BMI (ρ=−0.41, $P_{\mathrm{spin}}=0.0009$, and Fig. 2*B*).

However, its spatial correlation with the effects of CRP was not significant (ρ=−0.23; $P_{\mathrm{spin}}=0.0815$; *SI Appendix*, Fig. S19). The relationship between AT and cortical thickness was only significant at 1 cortical area (premotor cortex) after correction for multiple comparisons with FDR ≤0.05 (Fig. 2 and *SI Appendix*, Fig. S18). Cortical effects remained consistent with the use of a coarser parcellation (*SI Appendix*, 2.3).

At the subcortical level, BMI was significantly predictive of increased volume in six subcortical areas, including the nucleus accumbens (β(SE)=0.0189(0.0068);t=2.78). In contrast, CRP was significantly predictive of decreased volume in three structures: pallidum (β(SE)=−0.0438(0.0068);t=−6.46), thalamus (β(SE)=−0.0251(0.0068);t=−3.70), and hippocampus (β(SE)=−0.0187(−0.0068);t=−2.76). Adult trauma was significantly predictive of reduced volume of all subcortical structures tested, including the nucleus accumbens (β(SE)=−0.0339(0.0068);t=−5.00); for details, see Fig. 2*B* and *SI Appendix*, Tables S9–S11. We complemented these analyses by examining the independent effects of childhood maltreatment on cortical thickness and subcortical structures with linear regression; there were no significant effects, indicating childhood maltreatment scores did not directly contribute to changes in brain structure; see *SI Appendix*, 2.4.

CRP, BMI, and AT brain maps remained consistent across nine subsamples corrected for different possibly confounding factors of mental health history, lifestyle factors, and immune and metabolic health; see *SI Appendix*, 1.4 and 3.1.

### Full and Sparse Path Models of Adult Trauma-, BMI-, and CRP-Mediated Effects of CM on Brain Structure.

**Fig. 3.**
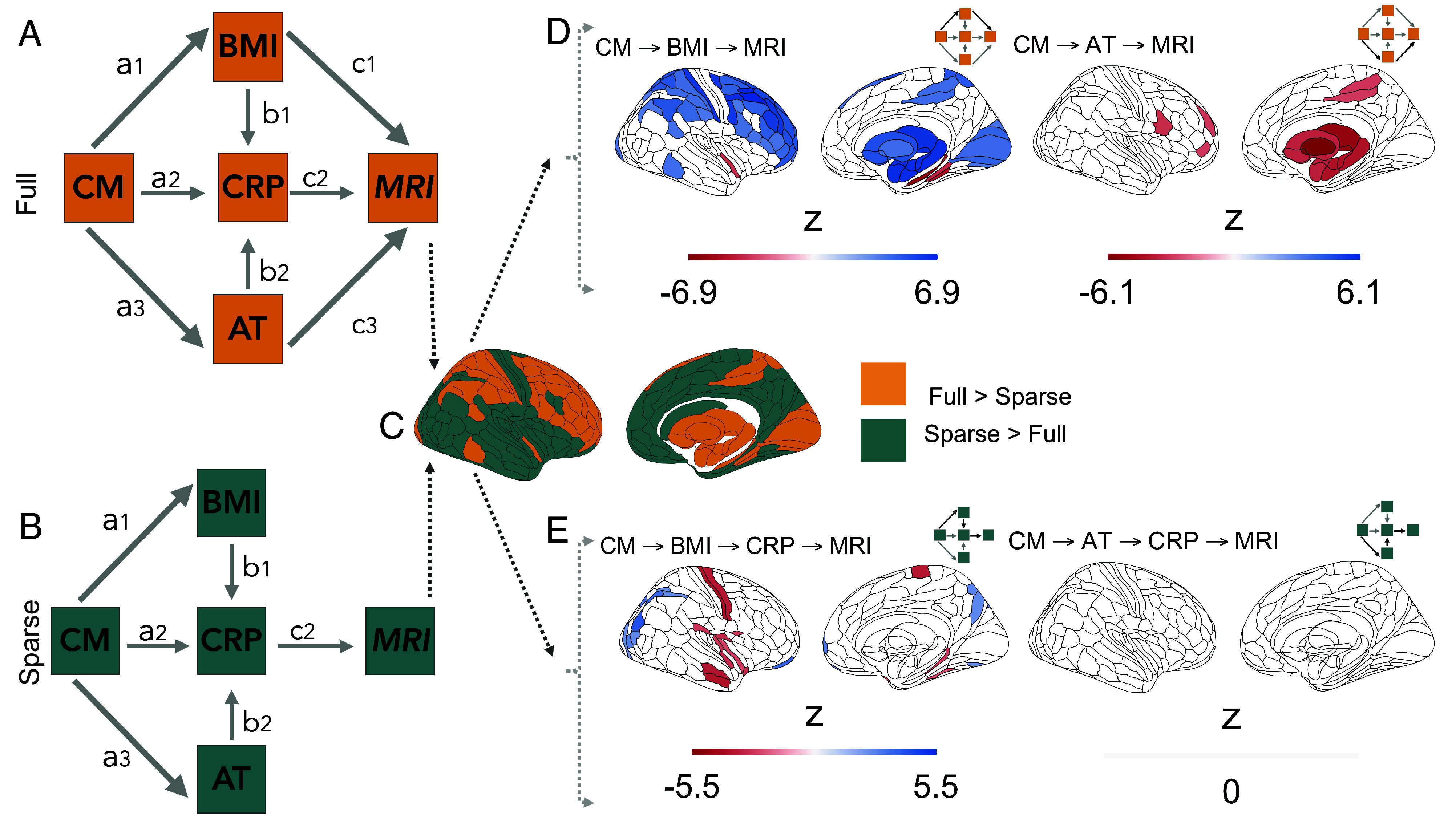
Indirect effects of childhood maltreatment (CM) on cortical subcortical structures can be mediated by direct effects on body mass index (BMI), C-reactive protein (CRP) or adult trauma (AT) in two nested path models (full and sparse). (*A* and *B*) Diagrams of nested path models of indirect effects of CM on brain structure. Full model (*A*): contains direct effects of BMI, CRP and AT on adult brain MRI measurements (paths c1,c2 and c3, respectively); all other paths between non-MRI variables are identical to the model in Fig. 1. Sparse model (*B*) contains a direct effect of CRP, only, on adult brain structure; adult trauma and BMI can have indirect effects on brain MRI measurements mediated by their direct effects on CRP. (*C*) Comparison of full and sparse models: This pair of nested models was evaluated at each of 180 cortical areas defined by the Glasser parcellation (36) and their difference in model goodness-of-fit was measured by the likelihood-ratio. Yellow areas indicate significantly better fit by the full model at FDR ≤0.05; green areas indicate where the sparse model was sufficient to explain local variance. (*D, left.*) Brain map of BMI-mediated indirect effects (Wald *z*) of childhood maltreatment on brain structure, together with a schema highlighting (in bold lines) the paths whose coefficients are combined to estimate the overall magnitude of the indirect effect. Blank areas on the cortical and subcortical maps indicate non-significant results (FDR ≥0.05) or locations where the model was not evaluated. Negative *z*-values indicate decreased grey matter (blue), and positive *z*-values indicate increased grey matter (red), associated with greater BMI predicted by greater CM. (*D, right.*) Indirect effects of childhood maltreatment on brain structure, mediated by adult trauma. Negative *z*-values indicate decreased grey matter (blue); positive *z*-values indicate increased grey matter (red), associated with greater AT predicted by greater CM. For thresholded and unthresholded maps of all paths evaluated see *SI Appendix*, Figs. S21 and S22; and for unstandardized coefficients see *SI Appendix*, Figs. S23 and S24. (*E, left*) Childhood maltreatment had indirect effects on adult brain structure that were mediated by a chain of paths from CM → BMI → CRP. Negative *z*-values indicate reduced CT was indirectly predicted by CM via its direct effects on BMI, which in turn could have effects on brain structure directly or via its effects on CRP. (*E, right*) There were no significant indirect effects of childhood maltreatment mediated by a chain of paths from CM to CRP via AT, i.e., CM → AT → CRP. For maps of all thresholded and unthresholded paths see *SI Appendix*, Figs. S25 and S26; for unstandardized coefficients *SI Appendix*, Figs. S27 and S28. Wald *z* scores represent the product of path coefficients (standardized by their standard errors) constituting each of the indirect effects of CM on brain structure evaluated by these two (full and sparse) models.

As BMI, CRP and AT were shown to independently relate to differences in brain structure (Fig. 2), as well as being affected in adulthood by CM exposure (Fig. 1), we next extended the path model of interactions between childhood maltreatment, adult trauma, BMI, and CRP to also include MRI measurements of cortical thickness or subcortical volume (Fig. 3 *A* and *B*). This increases the degrees of freedom to estimate path model parameters from six (based on a correlation matrix of four variables) to 10 (based on a correlation matrix of five variables) at each brain region. As in the prior model (Fig. 1), not inclusive of MRI, childhood maltreatment had direct effects on adult trauma and BMI, which both had direct effects on CRP. We fitted and compared two versions of the MRI-inclusive model to test the hypothesis (H3): Effects of CM on adult brain structure are mediated by its parallel effects on adult trauma, BMI, and CRP.

The two nested models were designated, respectively, full and sparse. The full model (eight parameters) specified that any of the three adult variables (AT, BMI, or CRP) could have direct effects on brain structure of each region (Fig. 3*A*). As these three variables all share the same independent variable, paths pointing to the given brain region are estimated as partial regression coefficients. The sparse model (six parameters) specified that only CRP had a direct effect on brain structure, i.e., any effects of adult trauma or BMI on brain structure must be mediated indirectly via their effects on CRP (Fig. 3*D*). Neither model passed the Satorra–Bentler scaled $\chi^{2}$ test at any region (all P<0.05), yet both models had good fit across the entire brain given their CFI, RMSEA, and SRMR scores (*SI Appendix*, 2.5).

To determine whether the full or sparse model represented a better account of the mediated effects of childhood maltreatment on brain structure, we estimated the likelihood ratio for the difference in goodness-of-fit between models at each region (FDR≤0.05). This comparative analysis of performance identified 56 cortical and six subcortical structures where the full model performed significantly better in accounting for the data than the sparse model. Whereas the sparse model, where CRP was the ultimate mediator of any indirect effects on brain structure, was a significantly better fit to the data at 124 cortical areas and 1 subcortical structure, the pallidum (Fig. 3*C*). These results suggested that no single model uniformly explained the mediated effects of childhood maltreatment across the brain.

### Indirect Effects of Childhood Maltreatment on Adult Brain Regional Structure (Mediated by BMI, CRP or AT).

To assess the size and significance of hypothetically anticipated indirect effects of childhood maltreatment on brain structure, mediated by the direct effects of childhood maltreatment on AT, BMI, and CRP, in the context of the direct effects of these three variables on brain structure (Fig. 2), we estimated the product of path coefficients for each chain of predictive relationships in both the full and sparse models. We exclusively estimated these coefficients within the set of brain areas where each model was optimally fit (Fig. 3*C*).

In the full model, the product of path coefficients (a1×c1) from {CM → BMI → MRI} was significantly greater than zero within all N=56 cortical and N=6 subcortical areas tested. The strongest indirect effects of childhood maltreatment mediated by adult BMI were concentrated in dorsolateral prefrontal, premotor, and primary somatosensory and motor cortical regions ($z_{\max}=6.89,\beta_{\max}=0.00501,P<0.0001$); and, subcortically, in the amygdala (z=6.43,β=0.00449,P<0.0001), thalamus (z=6.33,β=0.00437,P<0.0001) and hippocampus (z=6.48,β=0.00455,P<0.0001) (Fig. 3*B*, *Left*).

There was also evidence for an indirect effect of childhood maltreatment mediated by adult trauma on brain structure estimated by the product of path coefficients (a3×c3) from {CM → AT → MRI}. This indirect effect passed the threshold for significance in six areas located within premotor, sensorimotor, inferior prefrontal and dorsolateral prefrontal cortical areas ($z_{\max}=-3.31,\beta_{\max}=-0.00718,P=0.0247$), but was significant for all subcortical structures at which the model was evaluated, with the strongest effects in pallidum (z=−6.84,β=−0.0133,P<0.0001), thalamus (z=−5.22,β=−0.0112,P=0.0002) and nucleus accumbens (z=−5.06,β=−0.0109,P=0.0003); see Fig. 3*B*, *Right*.

There were no significant CRP-mediated effects for the product of the coefficients (a2×c2) from {CM → CRP → MRI} (*SI Appendix*, Fig. S21). However, effects mediated by the path {CM → BMI → CRP → MRI} were borderline significant for one cortical area (z=3.18,β=0.00080, P=0.0463) and significant for three subcortical structures: pallidum (z=−6.06,β=−0.00178,P<0.0001), thalamus (z=−5.50,β=−0.00156,P<0.0001) and hippocampus (z=−5.12,β=−0.00140,P<0.0001); see *SI Appendix*, Fig. S21.

In the sparse model, there was some evidence that indirect effects of childhood maltreatment could be mediated by the longer chain of connections from {CM → BMI → CRP → MRI} although the product of path coefficients was small ($z_{\max}=3.96,\beta_{\max}=0.00091,P=0.0009$) and only significant in 27 cortical areas (Fig. 3*E*, *Left*). There was no evidence for indirect effects mediated by {CM → AT → CRP → MRI} in the cortex or subcortex (Fig. 3*E*, *Right*).

These results remained consistent in nine sensitivity analyses designed to test for possibly confounding effects of mental health history, lifestyle factors, and immune and metabolic health on the pattern of relationships between AT, CM, BMI, and CRP. These analyses were carried out by repeating the entire analysis pipeline (*SI Appendix*, 1.4) and examining the correspondence in the pattern of results between our principal analysis and multiple sensitivity analyses specified to address each potentially confounding factor for paths a1∗c1, a3∗c3, and a1∗b1∗c2; see *SI Appendix*, 3.2.

### Discussion.

In this study, we addressed three hypotheses concerning pathways linking retrospectively assessed childhood maltreatment to differences in adult brain structure. We tested (H1) that self-reported experiences of childhood maltreatment partly explain adult inflammation through their effects on adult BMI and trauma; (H2) that adult trauma, BMI, and CRP are significantly associated with variation in adult brain structure; and (H3) that childhood maltreatment can have indirect effects on brain structure mediated by its direct effects on adult trauma, BMI, and CRP, compounded by the direct effects of these adult variables on the brain. Results largely supported these hypotheses and indicated plausible mechanisms of linkage between early life adversity and adult variables, including brain structure.

#### Childhood maltreatment’s influence on BMI, CRP, and adult trauma.

We first examined how childhood maltreatment was related to adult trauma, BMI, and CRP. Path modeling suggested a predictive role for childhood maltreatment in higher BMI and higher levels of self-reported adult trauma. We also found that, although childhood maltreatment does not have direct effects on adult CRP, or near-negligible effects only discoverable in the much larger *N* = 116,887 UKB cohort, it can have proinflammatory effects indirectly via its direct effects on BMI and adult trauma. Higher blood concentrations of proinflammatory signaling molecules have been well documented in adults exposed to maltreatment in childhood (9). However, there are not yet sufficient mechanistic explanations for this association (37). Our results indicate that adult obesity following childhood maltreatment could be a driver of systemic inflammation in adulthood, and that this proinflammatory state can be exacerbated by re-exposure to traumatic stressors in adulthood.

Childhood maltreatment is linked to a host of atypical cognitive processes underlying maladaptive behaviors that likely mediate the increased risks for obesity and traumatic experiences in adulthood (38, 39). Such atypical cognitive processes include heightened social threat monitoring (40–43); impaired learning and cognitive control, i.e., executive dysfunction and increased emotional reactivity (44–46); and reduced reward sensitivity (47–49). These differences in cognition and behaviour are believed to originate from reduced volumes and altered connectivity in salience and prefrontal-amygdalar, fronto-striatal, and fronto-parietal networks; circuits involved in threat-detection, reward processing, and cognitive control, respectively (50).

The cognitive impact of maltreatment may act in tandem with other early life stress-induced physiological alterations and give rise to a so-called “thrifty phenotype” that leads to increased BMI (14). This phenotype is distinguished by greater energy intake and storage and/or decreased energy expenditure (14). In this context, individuals are driven to the consumption of calorie-dense foods through reduced reward sensitivity and executive dysfunction (39, 51), emotional dysregulation-induced use of food as a soothing mechanism (46), and neuroendocrine-mediated deviations in appetite (52). Conversely, lower energy expenditure is often regarded as a downstream consequence of obesity primarily due to excessive food intake, where inflammation-associated fatigue and increased physical mass convergently reduce daily activity levels (52, 53).

Higher rates of adult trauma may also be caused by the cognitive sequelae of childhood maltreatment. For instance, social cognition in maltreated adolescents is best explained by the severity of their exposure to these experiences (54). Adolescents who have experienced maltreatment as children are seen as less socially competent by their peers and teachers (55, 56); and, as adults, they have more dysfunctional interpersonal relationships (57). Heightened monitoring of social threats, challenges in cognitive control over emotions, and lower responsivity to social rewards may drive difficulties in social functioning by decreasing an individual’s ability to successfully negotiate complex social environments, and by increasing life-time vulnerability to stressors and material precariousness (58).

#### How can BMI and adult trauma influence inflammation?.

Body mass index scales linearly with mass of visceral adipose tissue, which is responsible for generating and regulating immune signaling molecules, including IL6 and other proinflammatory cytokines (59). In human observational studies and animal experiments, greater fat mass is robustly positively correlated with increased blood cytokine concentrations (60), making obesity a cause of systemic inflammation. The pathway between self-reported adult trauma and heightened CRP levels is less obvious, though the neuroendocrine system is a candidate mechanism for linkage. The neuroendocrine system is involved in the regulation of physiological responses to stress, and eventual recovery of homeostasis, via the hypothalamic–pituitary–adrenal (HPA) axis which releases and regulates cortisol, an anti-inflammatory glucocorticoid hormone (61, 62). Chronic trauma or stress leads to sustained cortisol secretion and ultimately dysregulation of glucocorticoid receptors leading to failure to down-regulate inflammatory signaling pathways despite high circulating levels of cortisol (20, 63).

#### CRP-, BMI-, and trauma-related variation in brain structure.

We found that CRP, BMI, and adult trauma were all independently related to brain structure. Adult trauma had relatively minor effects on cortical thickness but was strongly associated with volume reductions in all subcortical structures tested. In contrast, BMI and CRP both had widespread and anatomically convergent effects on cortical structure, with divergent and more localized effects on subcortical volumes (Fig. 2). Specifically for the subcortex, BMI was associated with general increases, while CRP was associated with significant reductions in half of the regions tested. This pattern of results suggests that cortical structure may be sensitive to maltreatment-induced dysregulation of metabolic and/or immune systems, whereas adult trauma impacts subcortical brain structure through different biological pathways that possibly converge with immune effects in the subcortex.

Lower subcortical volumes in relationship to adult trauma have been previously reported (28, 64). Limbic structures are rich in GRs and expression density of these receptors can be down-regulated by chronic cortisol secretion (65), subsequently inducing deleterious changes in neuronal plasticity and integrity (66) and leading to macroscopic reductions in volume. This may constitute an independent pathway by which subcortical brain structure is modulated by trauma in adulthood.

The strongest signal for joint immuno-metabolic effects of CRP and/or BMI was increased cortical thickness in prefrontal, frontal, and somatosensory and premotor cortical areas. We also observed decreased thickness of insular, frontal opercular and primary somatosensory cortices in association with increased BMI and CRP, and decreased thickness of the medial temporal cortex associated specifically with higher BMI. While immuno-metabolic effects in the periphery are known to exert reciprocal influence on each other’s function, this process is less well understood inside the central nervous system (34). Our finding of a highly overlapping brain map of immuno-metabolic effects (Fig. 2*B*) suggests that, at a macroscopic level, the interaction between immune and metabolic pathways leads to similar outcomes for the cerebral cortex. This pattern of effects on brain structure could be driven by systemic circulation of proinflammatory cytokines, which can trigger astrocyte-dependent neuroinflammatory processes (53) and subsequent neuronal damage, leading to apparent increases and decreases in gray matter thickness and volume (53). However, increases can be a signal of both deleterious and neuroplastic effects (67): Greater hippocampal volumes are observable in animal models of both exercise and high-fat diets, with the latter being exclusively driven by histologically confirmed neurogenesis (67). A putative mechanism for volumetric increases in high-fat diets is an inflammation-driven increase in extracellular free-water content, which has been shown to scale linearly with CRP across cortical regions of the default-mode network in humans (68), and across most of the brain with increasing BMI (69, 70).

We report extensive cortical regional associations between greater BMI and increased cortical thickness. Accordingly, global CT increases with increasing BMI have been previously observed (71) and our overall pattern of effects is echoed in previous studies of gray matter structure in relation to increasing BMI. We replicate the reductions in thickness of temporal and medial-frontal regions (72–75) and even though the trend across prior volumetric studies is toward cortical volume reductions (25, 76, 77), some studies reported increased volume of occipital and frontal cortical regions with greater BMI (78–80). Prior trends in association of BMI with subcortical volumes are mixed (25, 72, 81, 82), although several studies find increases in nucleus accumbens (81), hypothalamus (78, 83) amygdala and hippocampus (72, 84) with greater BMI.

Heterogeneity of results across the published literature might emerge due to methodological variability. For instance, positive associations between volume and BMI might have been previously obscured by sample size and composition: Nonsignificant volumetric increases in hippocampal (85) and occipito-frontal regions (86) have been reported in small sample (N<100) studies, with more widespread cortical and subcortical effects being apparent with larger (N>1,000) samples (72, 80) or extensive exclusion criteria for cardiovascular and neuropsychiatric diseases (78, 79). This suggests that the full pattern of medial frontal and temporal cortical decreases, and frontal and occipital cortical increases, associated with obesity might have been previously attenuated by lack of statistical power.

#### Pathways from childhood maltreatment to variation in adult brain structure.

Finally, we found that the effects of BMI, CRP, and adult trauma on brain structure could be partially attributed to childhood maltreatment’s prior effects on these adult variables. Our comparison of full vs sparse path models suggested that indirect effects of childhood maltreatment mediated by adult trauma caused decreased volume of subcortical structures (Fig. 3*B*), in regions which also showed volume reduction as a direct effect of adult trauma (Fig. 2*A*). Indirect effects of childhood maltreatment mediated by BMI and CRP, formalized by a predictive chain of the form {CM → BMI → CRP → MRI} also caused reductions in volume in some regions of the subcortex as well as decreased thickness of temporo-parietal and other cortical areas (Fig. 3*E*), including the motor strip, which showed a similar pattern of thickness reduction as a direct effect of CRP (Fig. 2*A*). Conversely, frontal, occipital, and subcortical increases in gray matter were better explained by a direct effect of BMI that was not contingent on the immune state indexed by CRP. Although small in absolute size, these effects are consistent with the hypothesis that BMI, CRP, and AT are involved in the genesis of psychopathology after childhood maltreatment (28, 87) as they are capable of mediating changes in brain structure.

By considering multiple and parallel pathways of childhood maltreatment’s indirect influence on adult brain structure, we identified two distinct physiological systems possibly enabling the biological embedding of early life stress across the lifetime: an immuno-metabolic path, primarily linked to widespread differences across the cortex and subcortex, and a putatively neuroendocrine pathway linked to reductions in limbic and subcortical volumes. Joint dysregulation across immuno-metabolic, neuroendocrine, and cardiovascular systems has been previously described as the core feature of allostatic load, i.e., the multisystem physiological dysregulation resulting from chronic stress (66, 88, 89), which appears to be a core feature of the long-term outcomes of childhood maltreatment (89, 90). Our results extend this notion by showcasing how multisystems dysregulation explained by childhood maltreatment could be linked to widespread and distinct differences in brain structure.

While we do not provide evidence for how immuno-metabolic and neuroendocrine pathways drive variation in cortical thickness and subcortical volume, the literature suggests that neurotoxicity induced by physiological dysregulation in these same systems is likely involved (20, 88, 91). However, our findings highlight how neurotoxic factors could have different consequences on brain structure depending on the putative causal pathway considered. This is likely because neuroendocrine dysregulation, which is the primary system expected to dysfunction under chronic stress, appears to primarily and directly affect regions rich in glucocorticoid receptors such as those in the subcortex (88). On the other hand, immuno-metabolic dysregulation is expected to primarily affect brain structure through inflammatory processes triggered by obesity (91) which have the capacity to exert widespread cortical effects (34). Our findings of regional variation in increases or decreases of MRI-derived measures of gray matter structure mediated by the immuno-metabolic pathway coincide with previous studies independently linking immune (68, 92) and metabolic dysregulation (93) to macroscopic brain structure. This suggests that inflammatory processes capable of modulating brain structure have consequences for neuronal health that vary on a region-by-region basis. Indeed, it is possible that the same neurotoxic process leads to neuronal death apparent as decreases in thickness in one region and, due to variability in the local cell milieu, increases induced by greater extracellular water content in another (68).

By examining a sample of over 21,000 participants, we have pinpointed population-level trends on possible pathways of childhood maltreatment’s long-term influence on brain structure. However, individual outcomes in macroscopic brain phenotypes, e.g., reductions as opposed to increases in volumes of the subcortex, will depend on how the interaction of all factors weighing on brain development (genetics, environment, etc.) ultimately bias the relative contribution of each pathway toward phenotypic variation (20). The extent to which the observed differences in brain structure comprise a signal of deviation away from normative brain maturation over the lifecourse will need to be further established via future studies using MRI data collected longitudinally. Of special promise are studies initiating in childhood, e.g., the ABCD developmental cohort (94), as these will render it feasible to test more complex developmental models of long-term iterative interaction between childhood maltreatment, early brain and behavioral changes predisposing to adult BMI, CRP, and trauma, and the brain and cognitive effects of these adult risk factors for psychopathology.

#### Limitations.

There are several limitations to this study. First, path models with cross-sectional data do not allow us to draw conclusions on causality. Therefore, inferences on the directionality of relationships between the contemporaneous variables (BMI, CRP, AT) are limited. However, the extent of model-to-data agreement allows us to affirm that the hypothesized relationships are in good agreement with observed correlations between variables (95). Second, we used retrospective self-report as an index of childhood maltreatment. Meta-analyses have shown that retrospective, as opposed to prospective, measures of CM are more sensitive to recall bias due to the effects of adult psychopathology (96). Third, we were also mindful that the pattern of relationships between AT, CM, CRP, BMI, and MRI variables might be confounded by a number of other unmodeled variables, in addition to the effects of age, sex, and SES that were already corrected prior to the principal analysis. In particular, we considered the potentially confounding influences of mental health disorders, lifestyle factors, and immune and metabolic physical health. We found that sensitivity analyses of all of these factors did not materially confound our principal results (*SI Appendix*, 3); yet the scope of these analyses was limited by the data available in UK Biobank. For instance, we were unable to correct for childhood SES, although it has been associated with differences in cognitive function that co-occur with other CM-related risks and likely enhances the adverse impact of CM (97, 98). Fourth, the UKB cohort is a low-diversity sample with individuals of largely white European descent (*SI Appendix*, 3.3) and greater socioeconomic status than the average population (32). The nonrepresentatively affluent status of the UKB cohort implies that the rates of CM and AT reported by UKB participants will be underestimates of the population prevalence of lived experience of maltreatment and trauma, in childhood and adult life. Future work should aim to replicate these findings in more demographically and socioeconomically diverse samples. Fifth, future studies should also aim to consider a wider range of potentially relevant immune and endocrine markers of childhood and adult stress effects, and a broader range of MRI phenotypes beyond the principal focus on regional macrostructural metrics in this study.

### Conclusions.

Here, we show that childhood maltreatment can exert long-term indirect influences on adult brain structure through the physiological response to chronic adversity in immuno-metabolic and psychosocial systems as indexed by BMI, CRP, and adult trauma. We therefore provide a mechanistic explanation as to how psychopathology risks may be sustained across the lifetime after childhood maltreatment.

## Materials and Methods

### Data and Sample.

Data were obtained from the UK Biobank (UKB) (study ref no. 20904) which is a cohort of approximately 500,000 participants (ages 39 to 73), recruited from the United Kingdom population between 2006 and 2010, who completed extensive biological phenotyping and were additionally assessed with a follow-up online mental health and behavior questionnaire between 2012 and 2013 (99). Ethical approval for the use of these data was provided by the UKB research ethics committee, and the Human Biology Research Ethics Committee, University of Cambridge (Cambridge, UK). Principal analyses focused on a subset of participants (N ∼ 40,000) for whom MRI data of the brain were acquired and both T1- and T2-weighted images passed quality control (QC) (UKB MRI sample). Eligibility criteria for this sample are detailed in *SI Appendix*, 1.1. UK Biobank participants who did not have MRI data but who otherwise met eligibility criteria formed an internal replication sample (UKB sample; *N* = 21,738) for non-MRI analyses; see *SI Appendix*, 1.1.

### Immune, Metabolic, and Psychosocial Phenotypes.

#### C-reactive protein (CRP).

C-reactive protein was measured in blood serum by high-sensitivity immuno-turbidimetric assays (units: mg/L), on a Beckman Coulter AU5800.

#### Body mass index (BMI).

Body mass index ($kg/m^{2}$) was derived from weight (kg) and height (m) measurements with a Tanita BC-418 body composition analyzer and a Seca 202 measuring rod respectively.

#### Childhood maltreatment (CM).

Self-reports of childhood maltreatment were measured by the childhood trauma screener (CTS), which yields a total sum score across five questionnaire items (100). Responses were acquired as part of the UKB mental health questionnaire (101). For details on questionnaire data acquisition methodology, see ref. 101. The CTS assesses emotional, physical, and sexual abuse, as well as emotional and physical neglect in childhood (102); for details see *SI Appendix*, 1.2.1.

#### Adult trauma (AT).

Adult trauma scores were defined as the sum total of scores on five items from the UKB mental health questionnaire, which assessed adult experiences corresponding to the early life experiences measured by the CTS (101); for details see *SI Appendix*, 1.2.2.. These items indexed emotional, physical, and sexual abuse in interpersonal relationships as well as poor closeness of relationships and financial security after age 16 (101).

### Brain MRI Phenotypes.

#### MRI data: preprocessing, quality control.

Details for MRI acquisition protocols in UKB can be found in *SI Appendix*, 1.3. Downloaded 3D-MPRAGE T1-weighted images were preprocessed according to the Human Connectome Project (HCP) minimal Freesurfer pipeline (36). This pipeline includes artifact removal, pial and cortical surface generation, cross-modal registration, and standard-space alignment. T2-weighted images were used to derive more accurate surface representations (36, 103). Subjects without T2-w scans whose images have been processed with the HCP pipeline have been shown to have biased morphometric measures (104) and therefore participants without T2-w data were excluded from this analysis. Freesurfer’s reconstruction quality was quantified with the Euler index (105). Each image had the cortex parcellated according to the Glasser brain atlas (360 cortical regions), from which the macrostructural measures of cortical thickness (CT) were estimated (35). Volumetric partitions of 14 subcortical structures were also defined by Freesurfer (106).

A regional measurement in an individual scan, i.e., $CT_{i,j}$ the cortical thickness of the *i*th region in the *j*th participant, was excluded from analysis if it was more than five median absolute deviations (MAD) from the regional median, i.e. $CT_{i,j}\pm 5MAD$. Both volume and CT measures were corrected for sex, age at the time of scanning, sex×age interaction, and the Townsend deprivation index as a proxy measure of socioeconomic status. We also corrected for batch effects due to scanning at multiple UKB imaging centers (107); position of the head and radiofrequency receiver coil; and frame-wise displacement (FD)—a frame-to-frame index of head motion derived from functional MRI data that can serve as a proxy measure of motion-induced bias during structural MRI scanning in the same session (108, 109); see *SI Appendix*, 1.3.

Finally, bilaterally homologous MRI measures were averaged for each participant, resulting in data on CT at each of 180 symmetrized cortical areas and volume of 7 symmetrized subcortical structures; *SI Appendix*, 1.3.3.

### Statistical Analyses.

All analyses were conducted in RStudio (R version 4.2.2) (110); *SI Appendix*, 1.3.5.

#### Distribution and nuisance correction of non-MRI phenotypes.

All non-MRI phenotypes (CRP, BMI, CM, and AT) displayed a high distributional skewness (*SI Appendix*, Fig. S2*A*). To correct for this, natural scale values were log-transformed (*SI Appendix*, Fig. S2*B*) and used as the basis for all analyses. Additionally, all measures were corrected (i.e., nuisance regressed) for sex, age at the time of baseline visit, sex × age interaction, and the Townsend deprivation index (socioeconomic status). For results of these regressions, see *SI Appendix*, Tables S5 and S6. Correlations between all variables were estimated with Spearman’s method.

#### Linear models correction for spatial autocorrelation.

We used linear regression to separately address the relationships between CRP, BMI, AT, and CM and brain structure (H2). These models were independent and did not account for other variables’ effects aside from age, sex, socioeconomic status, and the interaction between age and sex. When examining the correlation between the resulting spatial maps, spin tests were used to derive *P*-values corrected for spatial autocorrelation (111).

#### Path analysis.

Path analysis can be regarded as an extension of multiple regression that allows for statistical inference on a hypothetical set of directed relationships between correlated variables. The hypothetical relationships are first depicted in a path model where the hypothesized direction of effects between any two variables, e.g., A→B, or direct path, is represented by an arrow starting at the predictor variable (*A*) and ending at the dependent variable (*B*). Path coefficients, e.g., *a*, characterize the sign and magnitude of these directed relationships. Coefficients are estimated by iteratively updating the model-implied covariance matrix Σ (built on the basis of the path model depiction) with the coefficients of highest likelihood given the covariance matrix *S* observed in the data (112). As a result, path estimates corresponding to directed relationships with a common dependent variable are equivalent to partial regression coefficients, whereas path coefficients where the dependent variable has a single predictor are equivalent to regular regression coefficients (33). Indirect effects between variables are represented by a chain of paths, e.g., A→B, B→C for the indirect effect of *A* on *C*, and a corresponding series of path coefficients, e.g., a,b. The product of the path coefficients a×b is a measure of the size of the indirect effect and, if significant, an indicator of mediation, such that the prediction of *C* by *A* is at least partially mediated through *B*.

Path coefficients were estimated using the “lavaan” package in R (113). To obtain reliable statistics despite remaining nonnormalities in the log-transformed data, we implemented robust maximum likelihood estimators with Huber–White standard errors (MLR) which are asymptotically equivalent to a Yuan–Bentler test statistic (113). MLR yields raw parameter estimates, the ratio of the parameter estimates and their SE (i.e., the Wald *Z* statistic or standardized parameter estimates), and the *P*-value for the Wald *z* statistic. Statistical inference on the significance of all paths (direct or indirect) was done on the basis of these *P*-values. For models including brain MRI variables, we corrected for multiple comparisons with the false discovery rate (FDR) at 5% where the number of tests considered was equal to the number of anatomical regions tested (N=187) in a symmetrized whole brain map. As the product of the multiple path coefficients constituting an indirect path (e.g., a×b) is normally distributed for large *N*s, there was no need to rely on nonparametric methods of statistical inference for indirect (mediation) effects (114); for further details, see *SI Appendix*, 1.3.4.

##### Assessment of model to data fitness.

We measured the fit of our path models to the data with the following measures: the Satorra–Bentler scaled $\chi^{2}$ fit test, the comparative fit index (CFI), the root mean square error of approximation (RMSEA) with its CI, and the standardized root mean squared residuals (SRMR) (115). We report the robust variants of these measures due to the use of an MLR estimator (116). We evaluated model goodness-of-fit by the following criteria: CFI, acceptable fit 0.95 to 0.97, good fit >0.97; SRMR, acceptable fit 0.05 to 0.10, good fit <0.05; and RMSEA, acceptable fit 0.05 to 0.08, good fit <0.05. Finally, we compare the relative fit of nested models to the data with chi-square-based comparisons of difference in the likelihood ratio. As this method is how we choose the set of brain regions in which to evaluate direct and indirect effects for a given model, we corrected for multiple comparisons with the more stringent familywise error rate (FWER) 5% Holm method. The number of tests considered was equal to the number of anatomical regions tested (N=187).

Note that the technical capacity to estimate directed or asymmetrical paths between variables does not double as a tool for providing evidence of causality when any two variables are contemporaneously measured. In such cases, a good-fitting path model can serve as a useful indicator of agreement between theory and data (117), but it does not substantiate an underlying causal mechanism.

#### Model contrasts.

The MLR fit functions used to estimate path coefficients yield a *t*-statistic which is the basis for comparison between nested models. For the UKB MRI sample, we fitted two nested models and contrasted their performance at each region with a likelihood-ratio test where the difference in *t*-statistics between the two models under the null hypothesis follows a $\chi^{2}$ distribution with *k* degrees of freedom where $k=p_{1}-p_{2}$ and $p_{1},p_{2}$ denote the number of path coefficients estimated in models 1 and 2, respectively. Likelihood ratio (LR) statistics were tested for statistical significance with FDR = 5% to correct for multiple comparisons and significant LR tests indicate that the data were best explained by the more complex model, with a larger number of path coefficients, at that region.

## Supplementary Material

Appendix 01 (PDF)

## Data Availability

All data were downloaded from the UK BIOBANK database (http://www.ukbiobank.ac.uk/) (99) under application number 20904. The UK BIOBANK provides summary statistics of its data via an associated data showcase, and data access is available after completion of an application via the UK BIOBANK’s online access management system (99). Code for the main analyses of this study can be found in the following GitHub repository: https://github.com/s-orellana/UKB_CM_Brain (118).
