# Supplementary material for: Childhood maltreatment influences adult brain structure through its effects on immune, metabolic, and psychosocial factors
Source: Proc Natl Acad Sci U S A. 2024 Apr 9;121(16):e2304704121. doi: 10.1073/pnas.2304704121 (PMC11032474; doi:10.1073/pnas.2304704121)
Supplement: Supplementary file 1 — Appendix 01 (PDF) [file pnas.2304704121.sapp.pdf]

SUPPLEMENT: Childhood maltreatment influences adult brain structure through its effects on immune, metabolic and psychosocial factors

Sofia C. Orellana[a,1], Richard A.I. Bethlehem[b*,a], Ivan L. Simpson-Kent[c*,d,e], Anne-Laura van Harmelen[f*,a,2], Petra E. Vértes[a,2], and Edward T. Bullmore[a,g,2]

[a]Department of Psychiatry, University of Cambridge, Cambridge CB2 0SZ, U.K.
[b]Department of Psychology, University of Cambridge, Cambridge CB2 3EB, U.K.
[c]Institute of Psychology, Leiden University, Leiden 2333 AK, The Netherlands
[d]MRC Cognition and Brain Sciences Unit, University of Cambridge, Cambridge CB2 7EF, U.K.
[e]Department of Psychology, University of Pennsylvania, Philadelphia, PA 19104-6241, U.S.A
[f]Institute of Education and Child Studies, Leiden University, Leiden 2333 AK, The Netherlands
[g]Cambridgeshire & Peterborough NHS Foundation Trust, Cambridge CB21 5EF, U.K.
[*]Current address
[1]Corresponding author: sdco2@cam.ac.uk
[2]These authors contributed equally to this work

March 13, 2024

Contents

SI 1 Supplementary Methods 6
SI 1.1 Sample selection . . . 6
SI 1.2 Immune, metabolic and psychosocial phenotypes . . . 8
SI 1.2.1 Childhood maltreatment (CM) . . . 8
SI 1.2.2 Adult Trauma (AT) . . . 8
SI 1.2.3 Phenotype log transform . . . 8
SI 1.3 MRI acquisition and processing . . . 8
SI 1.3.1 MRI post-processing and quality control . . . 9
SI 1.3.2 Imaging nuisance variables details . . . 9
SI 1.3.3 Bilateral atlas construction . . . 10
SI 1.3.4 Estimation and inference of indirect path model effects . . . 11
SI 1.3.5 Software . . . 11
SI 1.4 Sensitivity analyses: procedure . . . 11
SI 1.5 Sensitivity analyses: continuous phenotypes . . . 12
SI 1.5.1 Anxiety scores . . . 12
SI 1.5.2 Depression scores . . . 12
SI 1.6 Sensitivity analyses: categorical phenotypes . . . 13
SI 1.6.1 Never diagnosed with a mental health condition . . . 13
SI 1.6.2 Exercise frequency . . . 13
SI 1.6.3 Non-smoking . . . 13
SI 1.6.4 No metabolic syndrome . . . 13
SI 1.6.5 Anti-inflammatory medication free . . . 14
SI 1.6.6 Inflammatory illness free . . . 14
SI 1.6.7 Anti-flammatory illness and medication free . . . 14
SI 1.7 Sensitivity analyses: Ethnicity demographics . . . 14

SI 2 Supplementary Results 15
SI 2.1 Childhood maltreatment and adult trauma sample characteristics . . . 15
SI 2.2 Relationships between childhood maltreatment, adult trauma, BMI and CRP in the larger UKB sample . . . 17
SI 2.3 Replication of effects of adult trauma, BMI and CRP on cortical thickness with a coarser parcellation. . . . 18
SI 2.4 Effects of CM on cortical thickness and subcortical structure . . . 19
SI 2.5 Complementary goodness of fit assessments . . . 20

SI 3 Sensitivity Analyses 23
SI 3.1 Sensitivity replication of H2 . . . 23
SI 3.2 Sensitivity replication of H3 . . . 26
SI 3.3 Sensitivity replication of H3 considering ethnicity . . . 31

SI 4 Supplementary Figures 32

SI 5 Supplementary Tables 43

List of Figures

SF1 Sample selection algorithm . . . 7
SF2 QQ plots of immune, metabolic and psychosocial phenotypes . . . 9
SF3 Symmetry of brain structural effects of AT, CRP, and BMI across hemispheres . . . 10
SF4 Cumulative percentages of CM and AT total questionnaire scores (sumscores) for the UKB MRI lager UKB samples . . . 16
SF5 Replication of relationships between childhood maltreatment, adult trauma, BMI and CRP in the larger UKB sample . . . 17

SF6 Unthresholded brain maps of independent relationships between adult trauma (AT), BMI, or CRP with coarser parcellation of the Glasser atlas 18
SF7 Independent effects of childhood maltreatment on cortical thickness and subcortical volume. 19
SF8 Full model goodness-of-fit indices density plots for the whole brain 21
SF9 Sparse model goodness-of-fit indices density plots for the whole brain 22
SF10 Cross-sample correlation matrix for t-values derived from the (Brain $\sim$ BMI) linear regression model evaluated across 187 regions of the brain for the principal analysis and 10 sensitivity analyses to address specific potentially confounding variables . . . 23
SF11 Cross-sample correlation matrix for t-values derived from the (Brain $\sim$ CRP) linear regression model evaluated across 187 regions of the brain for the principal analysis and 10 sensitivity analyses to address specific potentially confounding variables . . . 24
SF12 Cross-sample correlation matrix for t-values derived from the (Brain $\sim$ AT) linear regression model evaluated across 187 regions of the brain for the principal analysis and 10 sensitivity analyses to address specific potentially confounding variables . . . 25
SF13 Diagram of procedure used to evaluate the consistency between the principal analysis and multiple independent sensitivity analyses of the indirect effects of CM on brain structure (H3) 27
SF14 Verification of H3: Sensitivity analyses of the effects of adult or lifetime mental health disorder diagnosis on indirect paths from CM to changes in brain regional MRI metrics. 28
SF15 Verification of H3: Sensitivity analyses of the effects of lifestyle factors on indirect paths from CM to changes in brain regional MRI metrics 29
SF16 Verification of H3: Sensitivity analyses of the effects of different physical health factors on indirect paths from CM to changes in brain regional MRI metrics 30
SF17 Verification of H3: Sensitivity analyses replicating key findings of indirect effects of CM on MRI metrics in the subsample of only white individuals. 31
SF18 Thresholded brain maps of independent linear relationships between adult trauma (AT), CRP or BMI and cortical thickness and subcortical volume 32
SF19 Correlation of adult trauma and BMI effects on cortical thickness 33
SF20 Brain maps of independent linear relationships between adult trauma (AT), CRP or BMI and cortical thickness and subcortical volume 34
SF21 Thresholded indirect (z) effects of childhood maltreatment on brain structure for all paths in the full model 35
SF22 Unthresholded indirect (z) effects of childhood maltreatment on brain structure for all paths in the full model 36
SF23 Thresholded unstandardised indirect (β) effects of childhood maltreatment on brain structure for all paths in the full model 37
SF24 Unthresholded unstandardised indirect (β) effects of childhood maltreatment on brain structure for all paths in the full model 38
SF25 Thresholded indirect (z) effects of childhood maltreatment on brain structure for all paths in the sparse model 39
SF26 Unthresholded indirect (z) effects of childhood maltreatment on brain structure for all paths in the sparse model 40
SF27 Thresholded unstandardised indirect (β) effects of childhood maltreatment on brain structure for all paths in the sparse model 41
SF28 Unthresholded unstandardised indirect (β) effects of childhood maltreatment on brain structure for all paths in the sparse model 42

List of Tables

ST1 Summary statistics for sensitivity characteristics in the UKB MRI subsample 12
ST2 Ethnicity break-down for the UKB MRI subsample 14
ST3 Number of regions remaining consistent (overlapping) across principal and sensitivity analyses. 26

ST4 Log transformed values of clinical data on UKB sample and sub-sample with MRI data available . . . 43
ST5 Nuisance regression model results for immune, metabolic and psychosocial on UKB Imaging sample . . . 44
ST6 Nuisance regression model results for immune, metabolic and psychosocial on the larger UKB sample . . . 45
ST7 Path model coefficients for the relationships between childhood maltreatment, adult trauma, BMI and CRP in the UKB MRI sample . . . 46
ST8 Path model coefficients for the relationships between childhood maltreatment, adult trauma, BMI and CRP in the larger UKB sample. . . . 47
ST9 linear regression results at each brain area with BMI as the dependent variable and cortical thickness or subcortical volume as an independent variable . . . 48
ST10 linear regression results at each brain area with CRP as the dependent variable and cortical thickness or subcortical volume as an independent variable . . . 52
ST11 linear regression results at each brain area with AT as the dependent variable and cortical thickness or subcortical volume as an independent variable . . . 56

SI 1 Supplementary Methods

SI 1.1 Sample selection

The UK BIOBANK provided participant data for this study. Principal analyses focused on a subset of participants invited for a multi-modal MRI follow-up measurement for whom, at the time of analysis, N=40,680 had images of the brain available for download. Exclusion criteria consisted of the following: (1) imaging data was deemed low quality (see SI MRI post-processing and quality control); (2) data on socieconomic status (Townsend Deprivation Index) was missing; (3) C-reactive protein assays were missing; (4) Body Mass Index data was missing; (5) any of the mental health questionnaire items relevant to this study were unanswered. Participants who met criteria 2-5 but for whom MR images were not acquired or were deemed low quality were selected as a replication sample for non-imaging analyses. Figure SF1 illustrates our sample selection protocol showing that the imaging sample has N=21,738 subjects and the non-imaging sample has N=116,887.

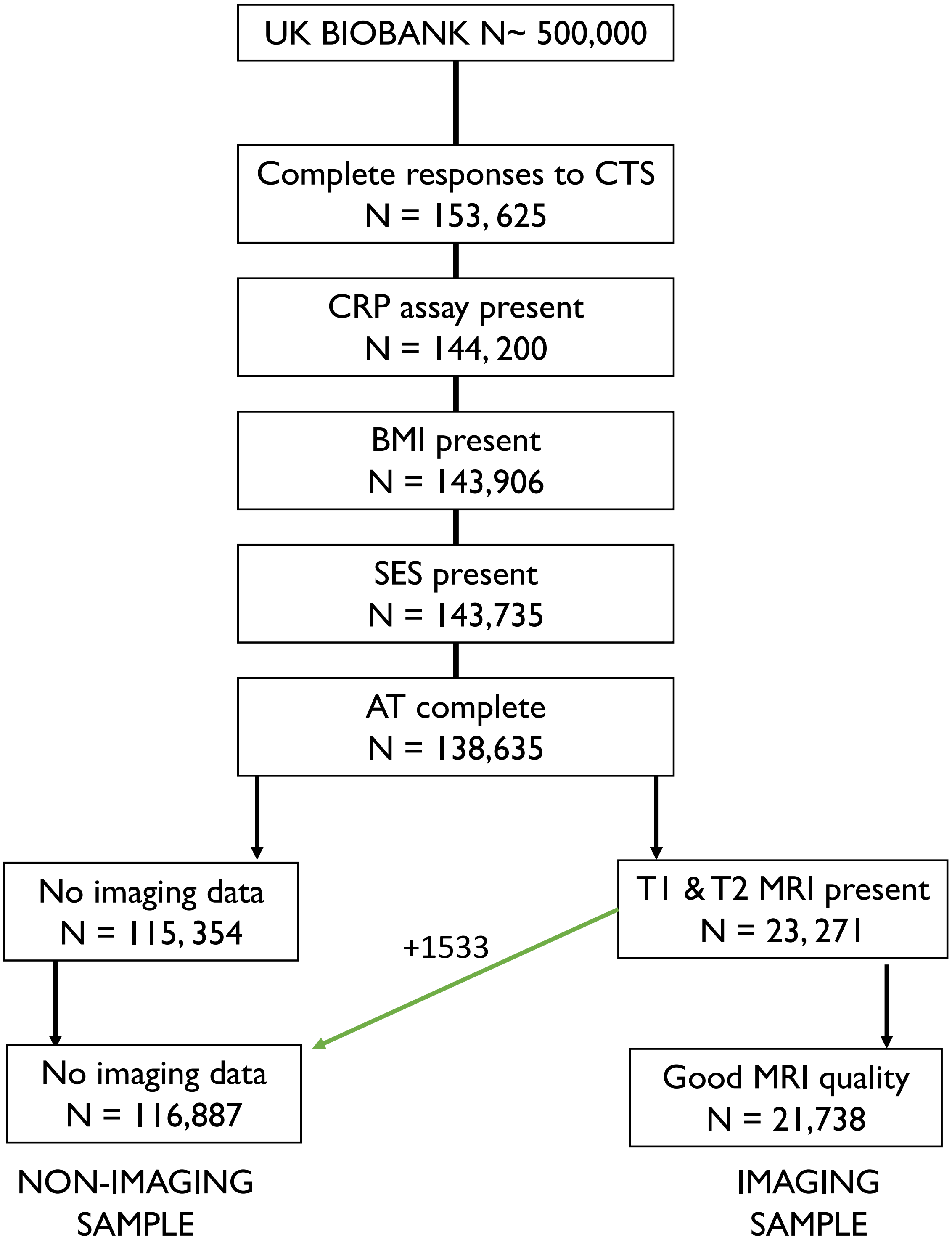

Figure SF1: Sample selection algorithm. CTS = childhood trauma questionnaire; BMI = body mass index; SES = socieconomic status as indexed by the Townsend Deprivation Index; AT = adult trauma questionnaire; MRI = magnetic resonance imaging.

SI 1.2 Immune, metabolic and psychosocial phenotypes

SI 1.2.1 Childhood maltreatment (CM)

Items corresponding to the childhood trauma screener (CTS) [12, 16] were extracted from the online mental health questionnaire developed by the UK BIOBANK Mental Health steering group [7] and a total "Childhoood Maltreatment" sumscore was built with them. The CTS is a shortened version of the commonly implemented childhood trauma questionnaire (CTQ) and its items can be divided into abuse and neglect subscales [5, 12].

SI 1.2.2 Adult Trauma (AT)

Adult trauma scores were derived from relevant items of the UK BIBANK online mental health questionnaire [7]. The questionnaire items from which the total sum scores were constructed are the following: (1) I have been in a confiding relationship (reversed); (2) A partner or ex-partner deliberately hit me or used violence in any other way; (3) A partner or ex-partner repeatedly belittled me to the extent that I felt worthless; (4) A partner of ex-partner sexually interfered with me, or forced me to have sex against my wishes; (5) There was money to pay the rent or mortgage when I needed it (reversed). These items were responses to the prompt “since I was sixteen”, indicating that subjects could report events relevant to the items that took place after childhood and early adolescence. Responses were given by a 0-4 Likert scale with 0 = “Never true” and 4 = “very often true.” Individuals responding “prefer not to answer” were excluded. Positive items were reverse coded, with higher total scores indicating greater trauma.

SI 1.2.3 Phenotype log transform

Due to the high distributional skewness (Figure SF2) of childhood trauma, adult trauma, BMI and CRP these variables were log-transformed. As CM and AT were coded from 0 to 4, 1 was summed to all scores in order to allow the mathematimatical computation of the log-transform. Transformed values then formed the basis of all analyses.

SI 1.3 MRI acquisition and processing

Structural magnetic resonance images of the whole brain were acquired on Siemens Skyra 3T scanners with 32-channel head coils. Acquisition took place with three identical scanners at three different dedicated imaging centers [20]. Reconstruction of images from k-space, and bias field correction took place in the scanner with standard Siemens software, without applying gradient distortion correction [3]. Further details on the UK BIOBANK's imaging protocol can be found in:

- http://biobank.ctsu.ox.ac.uk/crystal/refer.cgi?id=2367
- http://biobank.ctsu.ox.ac.uk/crystal/refer.cgi?id=1977

Next, 3D MPRAGE T1-weighted scanner-pre-processed images were then pre-processed according to the Human Connectome Project (HCP) minimal Freesurfer pipeline [15]. Processing included, artifact removal, pial and cortical surface generation, cross-modal registration, and alignment to standard space. When available, T2-w FLAIR images were used in order to derive more accurate surface representations [15, 13]. Euler indices were derived as a proxy measure of Freesurfer's reconstruction quality [27]. The cortex was anatomically segmented according to the Glasser brain atlas

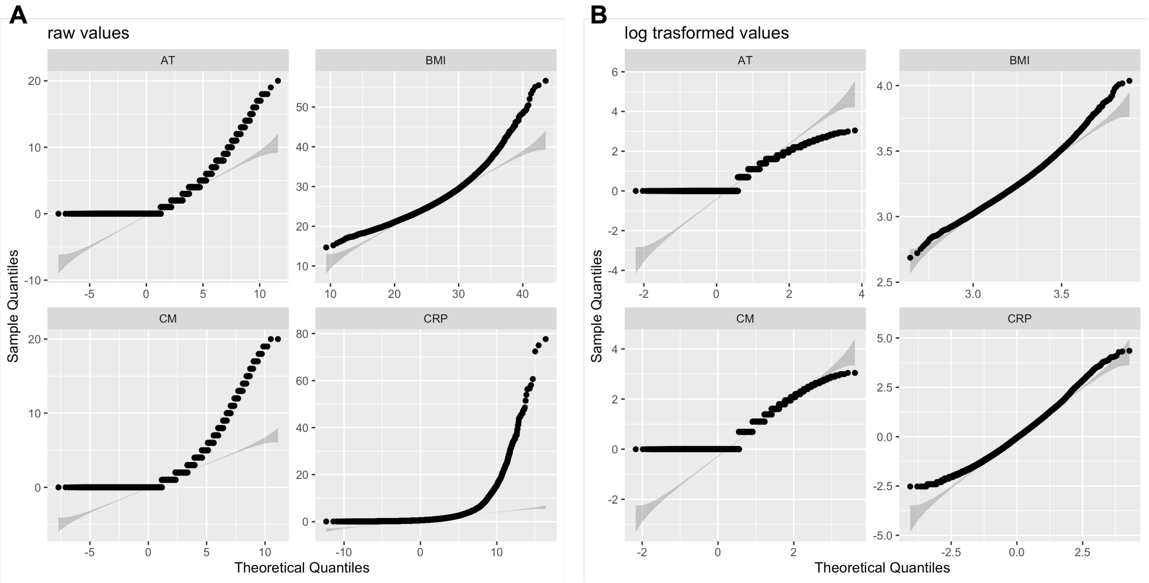

Figure SF2: QQ plots of immune, metabolic and psychosocial phenotypes. A. Raw values as supplied by UK BIOBANK (CRP and BMI) or as sumscores of UK BIOBANK questionnaires (childhood maltreatment (CM) and adult trauma (AT). **B.** log_{10} transformed phenotype values

into a total of 360 regions [14], and cortical thickness (CT) estimates were derived for each. Volumetric partitions of the subcortex were yielded from the ASEG atlas[10]. We only make use of the resulting grey matter structures in analyses: thalamus, caudate, putamen, pallidum, hippocampus, amygdala and accumbens.

SI 1.3.1 MRI post-processing and quality control

Cortical thickness estimates have been shown to be consistently biased if these were estimated without T2-w FLAIR input [18, 2]. Therefore, subjects without these data were excluded from imaging analyses. In this sample, those without a T2-w image had consistently lower mean cortical thickness values [33]. This is in line with previous observations that absence of FLAIR images for pre-processing is an important source of confounds in UK BIOBANK [2]. Additionally, and prior to nuisance correction, we excluded a given individual's ROI from analyses if it was a CT or volume outlier with a deviation 5 times the median absolute deviation ($\mp$ 5 MAD).

SI 1.3.2 Imaging nuisance variables details

Both CT and volume estimates were corrected for imaging confounds. The basic confounds considered were sex, age at scanning, the sex and age interaction, and the Townsend deprivation index - our proxy measure of socioeconomic status (SES). Additional parameters included BIOBANK imaging centre effects [9], Freesurfer's Euler index –a reliable measure of data quality [27]-, head position in the scanner, and framewise displacement (FD). FD is an index of frame-to-frame head motion derived from functional MRI data. Head motion during structural image acquisition is capable of introducing artifacts that bias CT and volume estimates [26]. FD has been shown to have high within- subject stability across different sessions of fMRI acquisition, and it is therefore possible to assume that it provides a sensible estimate of subject motion during structural MRI acquisition [30]. Finally, positioning of both the head and radio-frequency receiver coil were considered due to their

tendency to vary across participants, potentially introducing bias. Head position was characterized through x,y,z coordinates, plus z- positioning of the coil.

SI 1.3.3 Bilateral atlas construction

We ran regression models for AT, BMI, CM and CRP independently as predictors of cortical thickness or subcortical volume across all 374 ROIs in the Glasser cortical and aseg subcortical atlases. Symmetrical effects can be observed for the cortex and subcortex across all variables (fig.SF3A&B) and these are shown to be highly correlated (fig.SF3C). Due to these observations we averaged region-wise left and right values of CT or volume, for the cortex and the subcortex respectively, yielding a new atlas of 187 ROIs (180 cortex, 7 subcortex).

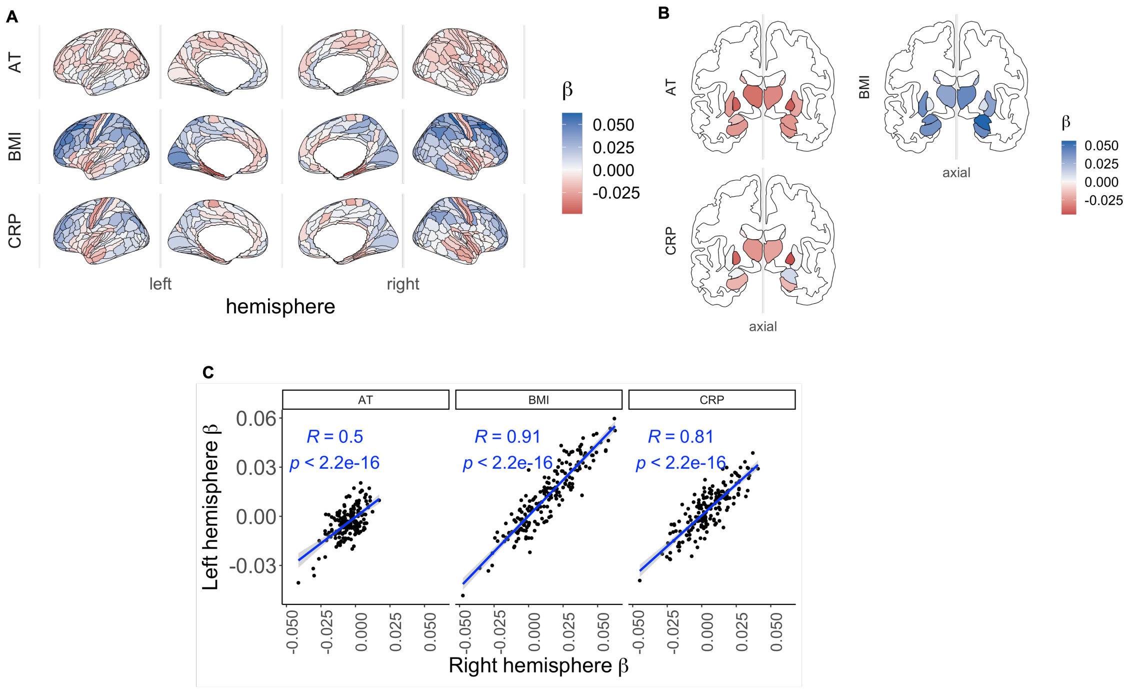

Figure SF3: Symmetry of brain structural effects of AT, CRP, and BMI across hemispheres. **A**. Independent linear effects for AT, CRP and BMI on cortical thickness for all non-lateralized 360 cortical areas. β unstandardized beta coefficients. **B**. Independent linear effects for AT, CRP and BMI on subcortical volumes for all non-lateralized 14 subcortical regions **C**. Scatterplots of unstandardized effects (β) correlations (Pearson, ρ) between left and right hemispheres.

SI 1.3.4 Estimation and inference of indirect path model effects

The product of the path coefficients $a \times b$ is not normally distributed for small Ns [6]. In such cases, inference on significance often relies on the construction of non-parametric sampling distributions for the product of coefficients through either resampling (e.g., bootstrapping) or simulation (e.g., Monte Carlo) procedures [8, 25]. However, for extremely large Ns, such as those in our UKB MRI sample, the joint distribution of the products approaches normality [4, 24]. Significance of the product of coefficients can then be assessed with the Delta method, lavaan's default choice for statistical inference, which solely relies on the variance of coefficients being multiplied to obtain the standard error and associated t-statistics of their joint distribution [32, 28]. Due the the large size of both of our samples we therefore relied on the Delta method to derive the p-values of indirect effects/paths.

However, for data that has been scaled before fitting the models lavaan is unable to correctly estimate the p-values of extremely small coefficients. For the indirect effects where this is the case, we only report the unstandardised coefficients and automatically treat these as non-significant.

SI 1.3.5 Software

All analyses were conducted in RStudio version 2022.12.0+353 running R version 4.2.2 using the packages: lavaan [28], dplyr [36], and tidyverse [37]. Plots were constructed with the package ggplot2 [35] and brain surface plots were created with ggseg and ggsegglasser [22, 22].

SI 1.4 Sensitivity analyses: procedure

Sensitivity analyses involved the re-testing of our entire analysis pipeline with slight modifications to the underlying data by considering additional categorical or continuous nuisance variables pertaining to mental health (anxiety and depression scores), lifestyle factors (exercise and smoking), and health factors relevant to this study (immune and metabolic factors). All sensitivity analyses were performed in the UKB MRI subsample; characteristics used to build the sensitivity subsamples can be found in Table ST1; description of these characteristics and how they were obtained can be found in sections SI 1.5 and SI 1.6.

For categorical sensitivity characteristics (described in SI 1.6) we first determined the percentage of the sample for whom the variable applied, e.g. the percentage of non-smoking individuals. Details can be found in table ST1. For characteristics that applied to 80% or more of the sample we simply excluded the rest of the individuals who did not contain this characteristic and repeated the entire analysis pipeline. For characteristics applying to $< 80\%$ we added the characteristic as a nuisance variable in the regression model principally used to correct for age, sex, and SES in all of the study's principal variables: CM, BMI, CRP, AT, and all brain regions. For *continuous* characteristics we repeated this same last step of considering the characteristic as a nuisance variable.

Table ST1: Summary statistics for sensitivity characteristics in the UKB MRI subsample. All characteristics were used to build a sensitivity subsample, except for those marked with *. Summary statistics of symptom scores for anxiety and depression exclude answers that were < 1

Characteristic	Number of participants (%) / Mean (SD)
Ever had anxiety*	6216 (29%)
Anxiety symptoms at worst episode	1.58 (0.34)
Ever had depression*	11364 (52%)
Depression symptoms at worst episode	1.68 (0.36)
Never clinically diagnosed with a mental health condition	15259 (70%)
Sufficient weekly vigorous exercise	10142 (47%)
Sufficient weekly exercise	15197 (70%)
Non-smoker	20437 (94%)
No metabolic syndrome	20047 (92%)
No inflammatory illness	18422 (85%)
No anti-inflammatory medication	18467 (85%)
Anti-inflammatory medication or inflammatory illness free	16314 (75%)

SI 1.5 Sensitivity analyses: continuous phenotypes

SI 1.5.1 Anxiety scores

Anxiety scores were derived from the UK Biobank online mental health questionnaire [7]. The questionnaire items that contributed to the total anxiety score, pertaining to the worst episode of anxiety recalled by each respondent, were the following: (1) difficulty concentrating; (2) frequent trouble falling or staying asleep; (3) feeling keyed up or on edge; (4) feeling more irritable than usual; (5) feeling restless; (6) tense, sore, or aching muscles. Participants responded to these items with either "Yes" or "No." The total anxiety score was computed as the sum of "Yes" responses across all items. Respondents who chose not to answer any of the items, or for whom there was not complete data available, were excluded from this sensitivity analysis. N= 6,216 participants (29% of the sample) reported ever having had anxiety; see Table ST1. Higher total scores indicated greater anxiety during the most severe episode of anxiety experienced by the respondent and all scores were log transformed before analysis.

SI 1.5.2 Depression scores

Depression scores were derived from the UK Biobank online mental health questionnaire [7]. The questionnaire items that contributed to the total depression score, pertaining to the to the worst episode of depression recalled by each respondent, were the following: (1) changes in sleep; (2) feelings of tiredness; (3) feelings of worthlessness; (4) difficulty concentrating; (5) thoughts of death; (6) weight change. Participants responded to these items with either "Yes" or "No." The total depression score was computed as the sum of "Yes" responses across all items. Respondents who chose not to answer any of the items, or for whom there was not complete data available, were excluded from this sensitivity analysis. N = 11,364 participants (29% of the sample) reported ever having had depression; see Table ST1. Higher total scores indicated greater depression during the most severe episode of depression experienced by the respondent and all scores were log transformed before analysis.

SI 1.6 Sensitivity analyses: categorical phenotypes

New sub-samples of the UKB MRI-subsample were created according to whether subjects met a specific set of criteria delineated by the variables below:

SI 1.6.1 Never diagnosed with a mental health condition

This subsample was constructed by excluding individuals who were ever diagnosed with mental health problems by a professional, according to self-reports, even if they did not currently have the diagnosis. This binary condition was constructed by offering respondents a set of mental health problems to choose from, which were combined into a single “Yes” or “No” answer. Individuals who chose not to reply, or who had missing data for this item, were excluded from the subsample.

SI 1.6.2 Exercise frequency

These samples were constructed by excluding individuals who did not meet two different criteria of weekly exercise.”Sufficient Weekly Vigorous Exercise” excluded individuals who did not meet the 2017 UK Physical activity guidelines of either 150 minutes of moderate or 75 minutes of vigorous physical activity per week. The subsample of ”Sufficient Weekly Exercise” expanded on the previous criteria and excluded individuals who did not meet the 2017 UK Physical activity guidelines of 150 minutes of walking, 150 minutes of moderate activity, or 75 minutes of vigorous activity per week. Individuals were excluded from a subsample when data for the corresponding criterion was missing.

SI 1.6.3 Non-smoking

This subsample was constructed by excluding individuals who report current tobacco use (smoking) on most days, all days or only occasionally. Individuals with missing data or who preferred not to report on their tobacco smoking behavior were also excluded.

SI 1.6.4 No metabolic syndrome

This subsample was constructed identifying individuals exhibiting features consistent with metabolic syndrome. Metabolic syndrome was defined in accordance with the latest provisional joint consensus guidelines from the International Diabetes Federation (IDF) and the American Heart Association/National Heart, Lung and Blood Institute (AHA/NHLBI) [1]. Specifically, an individual was considered to have metabolic syndrome when three or more of the following conditions were present: (a) Hyperglycemia: defined as fasting glucose levels of 5.56 mmol/L or higher, or a self-report of a diabetes diagnosis. As no differentiation was given between type 1 and type 2 diabetes in the UKB, either was considered. (b) Raised blood pressure: defined as systolic pressure of 130 mmHg or higher and diastolic pressure of 85 mmHg or higher, or a self-report of a previous hypertension diagnosis. (c) Elevated triglycerides: defined as triglycerides levels of 1.7 mmol/L or higher. Low HDL cholesterol: defined with sex-specific HDL cholesterol cutoff points of less than 1.03 mmol/L for males and less than 1.29 mmol/L for females. Individuals were not considered for a metabolic syndrome label if data for any of these conditions was missing. Additionally, the criterion for central obesity was not considered when defining this subsample as this would forcefully restrict the variance of BMI, a central variable across all hypotheses tested.

SI 1.6.5 Anti-inflammatory medication free

This subsample was constructed by excluding participants self-reporting the use of medications capable of biasing immunological assays [17, 19]. In the UKB, participants were asked to report any medications used/taken. Only medications taken daily, every month, or every three months were recorded [23].Following the procedure by Wu and colleagues [39] the list of all medications taken by UKB participants (N=6745) was narrowed down to only those used by at least at least 10 participants in the full sample; a total of N=1809. These medications were then classified by their active ingredients according to the Anatomical Therapeutic Chemical (ATC) Classification System [29], and further subdivided into 184 subgroups given by the first three levels of the ATC classification [39]. Any participant self-reporting the use of medications in the following subgroups, which were deemed capable of biasing immunological assays, were excluded: Anti-inflammatory, anti-rheumatoid, immunosuppressants medications and drugs relevant to the treatment of diabetes. We warranted an exception for 'ibuprofen' -an anti-inflammatory drug- given its common over-the-counter use. Participants with missing medication data or who did not report on their medication use were also excluded.

SI 1.6.6 Inflammatory illness free

This subsample was constructed by excluding participants who self-reported the following inflammatory illnesses capable of biasing immunological assays [17, 19]: cardiovascular, chronic liver and kidney disorders, diabetes type I and type II, respiratory and pulmonary disorders, including asthma.

SI 1.6.7 Anti-flammatory illness and medication free

This subsample was constructed by excluding all participants meeting the criteria for either the "anti-inflammatory medication free" subsample or the "inflammatory illness free" subsample.

SI 1.7 Sensitivity analyses: Ethnicity demographics

We provided additional sample characteristics of self-reported ethnic background in the UKB MRI subsample, see Table ST2. The percentage of non-white individuals in the sample was too small for a meaningful analysis of ethnic differences. Therefore, a new subsample of ethnically-white individuals only was built, and sensitivity analyses repeating the entire analysis pipeline with it were performed.

Table ST2: Ethnicity break-down in the UKB MRI sample

	Ethnicity	Number of subjects	Percentage
1	Asian or Asian British	694	3.19
2	Black or Black British	46	0.21
3	Chinese	54	0.25
4	Mixed	586	2.70
5	Other ethnic group	102	0.47
6	White	20204	92.94
7	Unanswered	52	0.24

SI 2 Supplementary Results

SI 2.1 Childhood maltreatment and adult trauma sample characteristics

We computed the cumulative distribution of childhood maltreatment (CM) and adult trauma (AT) total sum of questionnaire item scores (sumscores) in both the larger UKB and the UKB MRI samples as displayed in Figure SF4. The total possible score in either questionnaire is 20 when the Linkert scale used to quantify the responses to each questionnaire item runs from 0 = "Never true" to 4 = "very often true." In a validation and standardization of the childhood trauma screener (CTS), the questionnaire from which the CM scores are computed [12, 7], a score of 8 has a cumulative percentage of 80% [38]. Comparatively, our equivalent score of 7 has a cumulative percentage of 96.2% in the UKB MRI subsample; see Figure SF4. European self-reports of childhood maltreatment place prevalence rates in Europe at 13% for sexual abuse, 12% for physical abuse, 22% for emotional abuse and 27% for general neglect [21]. When dichotomizing self-reports across all items in the Abuse and Neglect subscales we have somewhat comparable rates for abuse: UKB MRI sample $n = 3429(15.8\%)$; UKB sample $n = 18847(16.1\%)$. As well as substantially lower rates of neglect: UKB MRI sample $n = 1584(7.29\%)$; UKB sample $n = 9433(8.07\%)$. These results indicate that the rates of CM in the UK BIOBANK are lower than in the general population. However, this is expected as it is well established that the UK BIOBANK cohort is generally wealthier and healthier that the general population [11]. No comparative prevalence rates are available for the AT scores as these are not a validated measurement tool, but rather a set of questions developed by UK BIOBANK to mimic the CTS items in adult life [7].

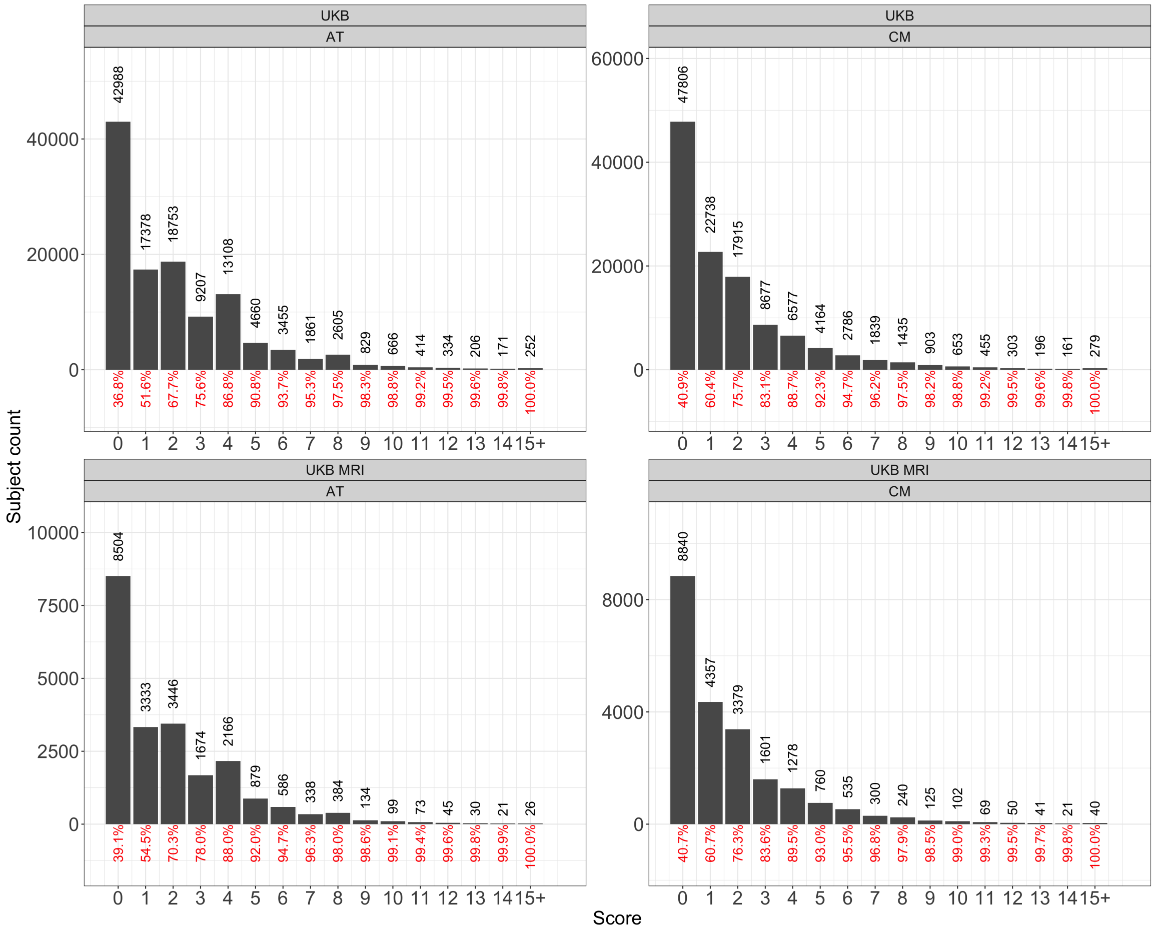

Figure SF4: Cumulative percentages of CM and AT total questionnaire scores (sumscores) for the UKB MRI lager UKB samples. The number of individuals in the sample with a given score is given in black at the top of the bar, and the corresponding cumulative percentages in red at the bottom.

SI 2.2 Relationships between childhood maltreatment, adult trauma, BMI and CRP in the larger UKB sample

We repeated analyses verifying our first hypothesis (H1) which states that BMI and AT mediate the relationship between CM and CRP. These replication analyses were conducted on all subjects of the UK BIOBANK who met our inclusion criteria but had no brain imaging data, or whose imaging data was of poor quality. H1 results were replicated on this sample except for the direct relationship between CRP and CM. In this sample, which is at least five times as large (N = 116,887), there was a direct of effect of childhood maltreatment on CRP ($CM \rightarrow CRP, z = 3.431, P < 0.001$). See Figure SF5B and Table ST8 for details. Model fit in this sample was good: $SRMR = 0.010; CFI = 0.997; RMSEA = 0.031$.

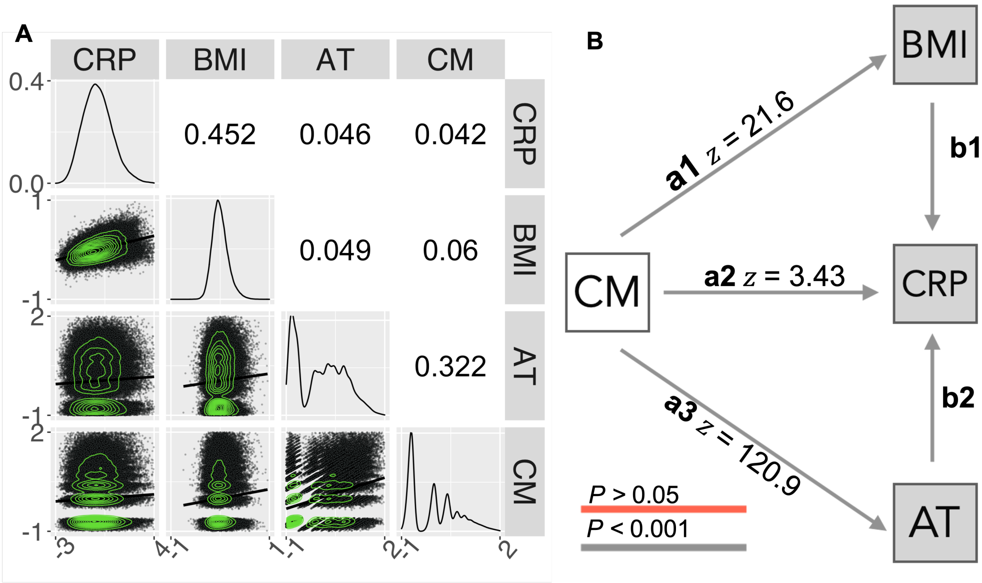

Figure SF5: Replication of relationships between childhood maltreatment, adult trauma, BMI and CRP in the larger UKB sample. **A**. Correlation matrix representing pair-wise Spearman's correlations (upper triangle) and scatterplots of the relationships between each pair of variables, with solid lines indicating fitted linear regression models (lower triangle). The diagonal represents the probability density function for each variable. All correlations were significantly greater than zero, with FDR $\leq$ 0.05. **B.** Path diagram representing direct effects of retrospectively ascertained CM (white) on the contemporaneously measured adult variables, AT, CRP, and BMI (grey). Standardized path coefficients are given as Wald (z) statistics

SI 2.3 Replication of effects of adult trauma, BMI and CRP on cortical thickness with a coarser parcellation.

We examined whether our results examining the effects of AT, BMI and CRP on CT remained consistent when using a coarser parcellation, or brain map, with a smaller number of areas. We leveraged the fact that the Glasser brain atlas groups each of its 180 areas into 22 distinct and spatially adjacent brain regions [14]. We then averaged cortical thickness of each set of areas within a given region, yielding 22 regional estimates of cortical thickness. This approach has been followed elsewhere [34]. Next, adult trauma, BMI and CRP were each treated separately as independent variables in three different linear regression models with cortical thickness as the dependent variable at each of the 22 *new* cortical areas. For all variables, the resulting brain maps remained qualitatively consistent with the main results relying on the more granular 180-area parcellation, see Figure SF6.

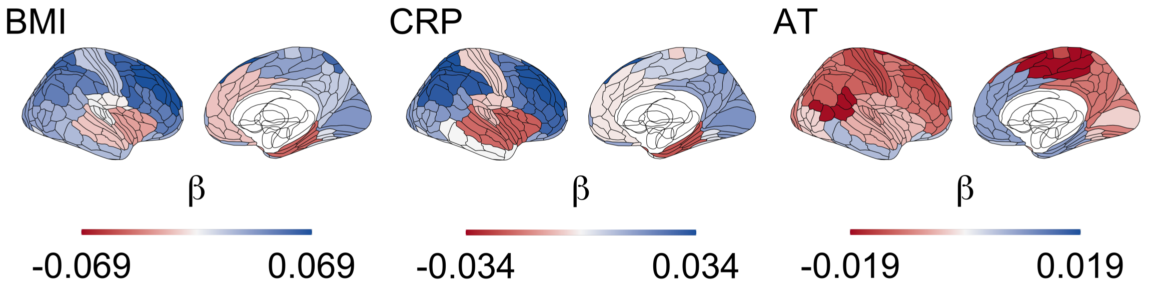

Figure SF6: Unthresholded brain maps of independent relationships between adult trauma (AT), BMI, or CRP with coarser parcellation of the Glasser atlas. Before obtaining empirical results, each area was grouped within one of Glasser's brain regions (or groupings) [14] and the average within-region cortical thickness was computed. Lines delineating the original brain regions are left as visual reference on the brain maps. β = unstandardised regression coefficients.

SI 2.4 Effects of CM on cortical thickness and subcortical structure

We constructed an independent linear regression model with childhood maltreatment scores as the predictor and cortical thickness or subcortical volume as the dependent variable. We found that CM scores did not significantly predict differences in cortical thickness or subcortical volume; see Figure SF7.

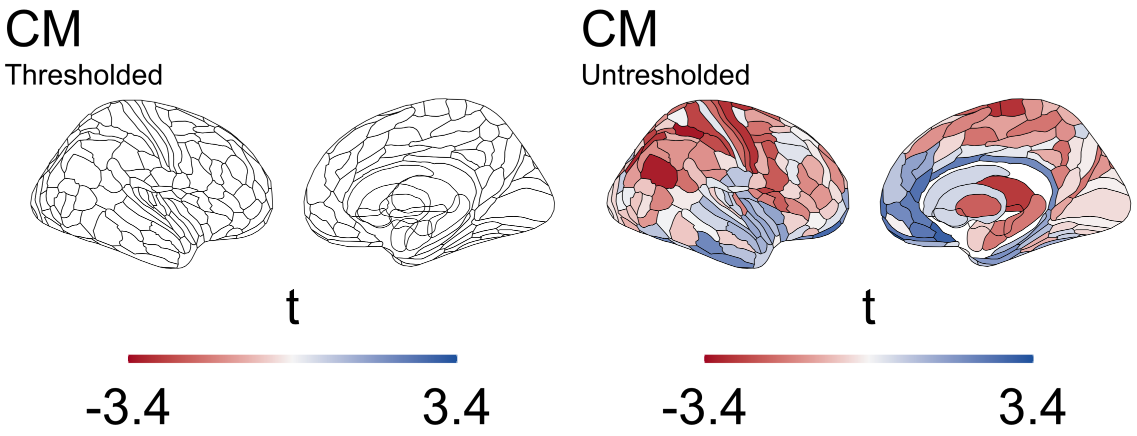

Figure SF7: Independent effects of childhood maltreatment on cortical thickness and subcortical volume. Cortical surface maps show the anatomical distribution of effect sizes(t-values) and the absence of significant effects of CM on brain structure after controlling for multiple comparisons with FDR$<$ 0.05-corrected thresholded effects.

SI 2.5 Complementary goodness of fit assessments

Following the convention in path modelling for evaluating goodness of fit, we supplement the Satorra-Betler χ^2 tests with the following additional indices: Comparative Fit Index (CFI), the root mean square error of approximation (RMSEA), and the standardized root mean squared residuals (SRMR) [31]. We evaluated each model at $n = 180$ cortical areas and $n = 7$ subcortical structures and derived their goodness of fit measures. Results for the full and sparse models can be seen in figures SF8 and SF9 respectively. Neither model passed the Satorra-Bentler scaled χ^2 test at any region (all $P < 0.05$). Note that this test is extremely sensitive to small discrepancies between the observed correlation matrix to which the model is fit and the matrix *implied* by a given path model. Therefore, significant p-values (which signal poor data and model agreement) are often expected for large Ns and fitness is evaluated jointly with other measures [31]. In this case both models can be considered to have good fit across the entire brain given their good CFI, RMSEA, and SRMR scores (see figures SF8 and SF9).

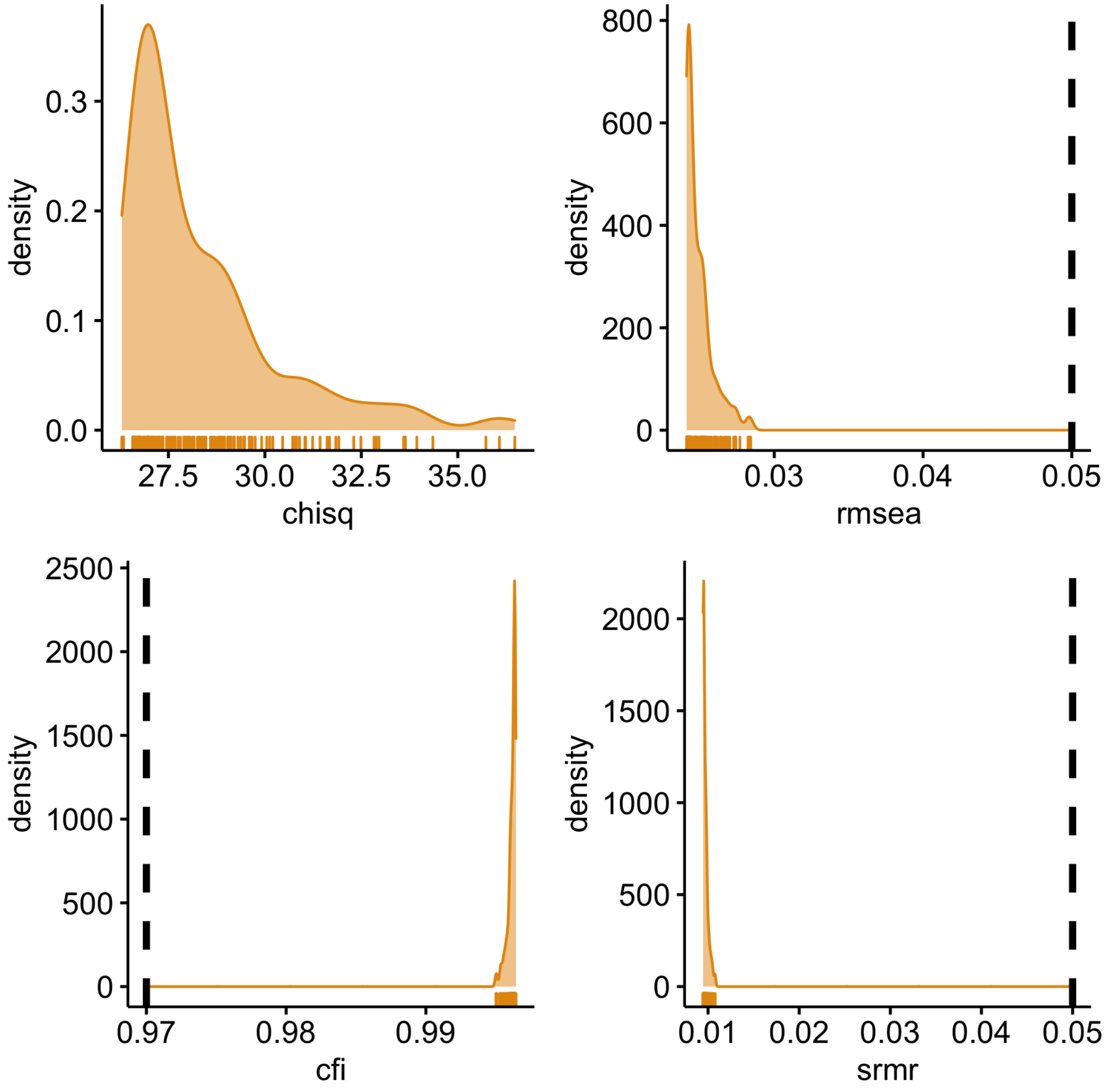

Figure SF8: Full model goodness-of-fit indices density plots for the whole brain. Dashed lines indicate good fit thresholds, where rmsea and srmr are deemed good if < 0.05, and cfi > 97.

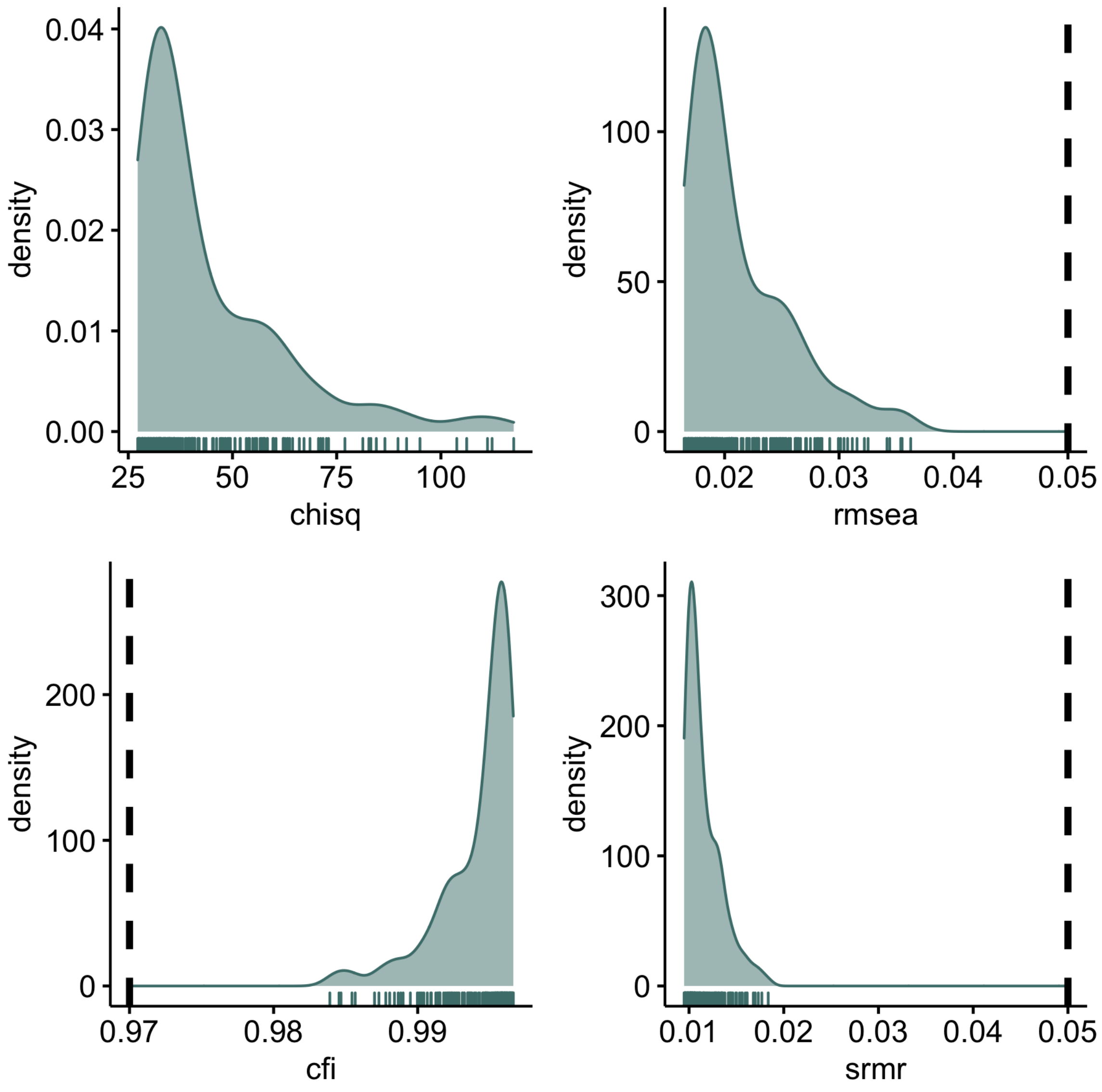

Figure SF9: Sparse model goodness-of-fit indices density plots for the whole brain. Dashed lines indicate good fit thresholds, where rmsea and srmr are deemed good if < 0.05, and cfi > 97.

SI 3 Sensitivity Analyses

SI 3.1 Sensitivity replication of H2

We repeated the entire analysis pipeline and verified our main results in relation to H2. For each sensitivity subsample we once again created three different linear regression models of the form (Brain $\sim$ BMI), (Brain $\sim$ CRP), and (Brain $\sim$ AT) where BMI, CRP and AT were treated separately as predictors and cortical thickness and subcortical volume as the dependent variable across 187 brain regions. Then, separately for BMI, CRP and AT we obtained the resulting t-values across 187 regions and correlated them across the original and sensitivity subsamples. Sensitivity subsamples were derived from the sensitivity characteristics highlighted in Table ST1. The resulting correlation matrices for BMI (Figure SF10), CRP (Figure SF11) and AT (Figure SF12) show that findings remain stable across all samples.

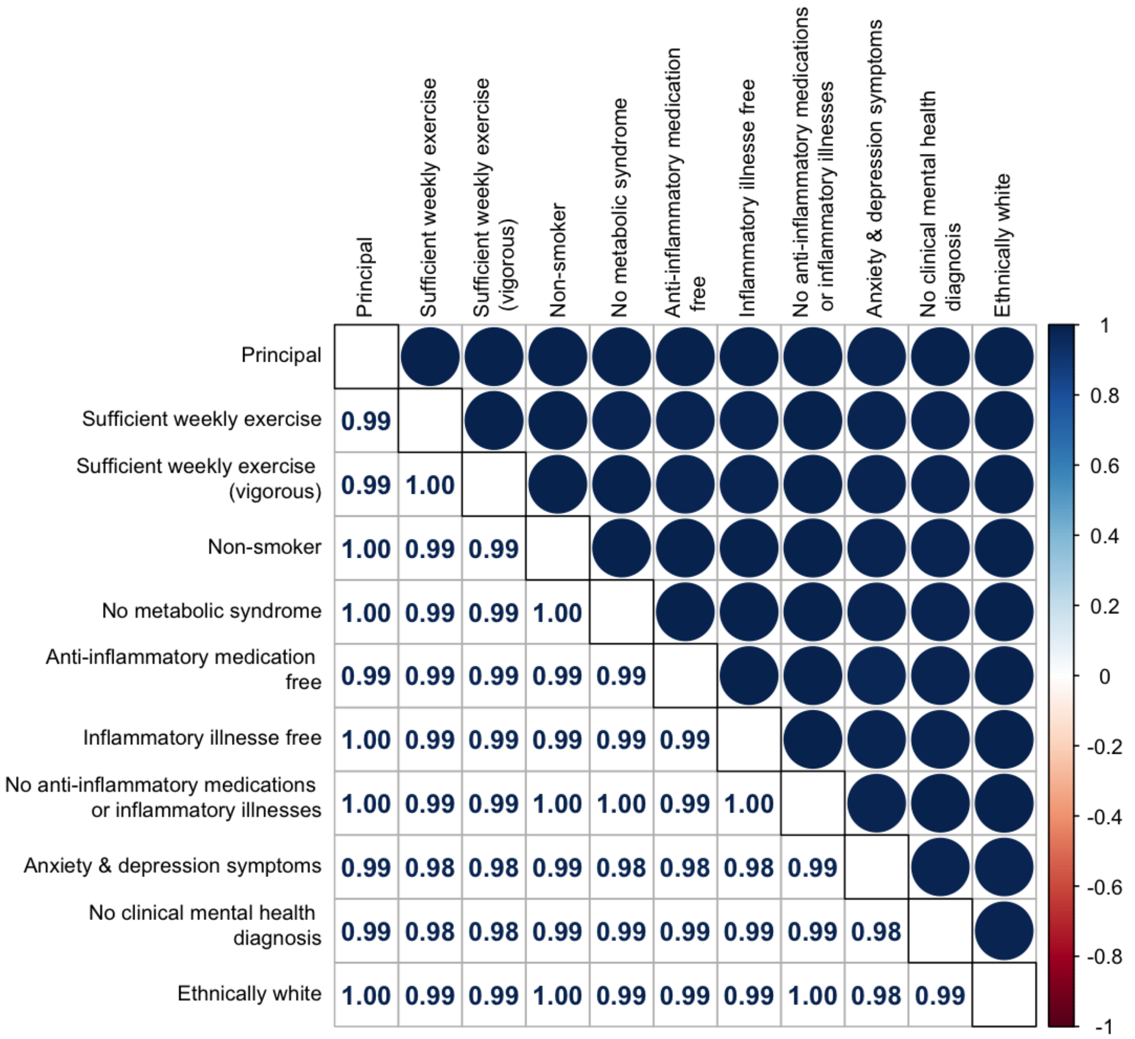

Figure SF10: Cross-sample correlation matrix for t-values derived from the (Brain $\sim$ BMI) linear regression model evaluated across 187 regions of the brain for the principal analysis and 10 sensitivity analyses to address specific potentially confounding variables. Columns and rows correspond to different sensitivity subsamples.

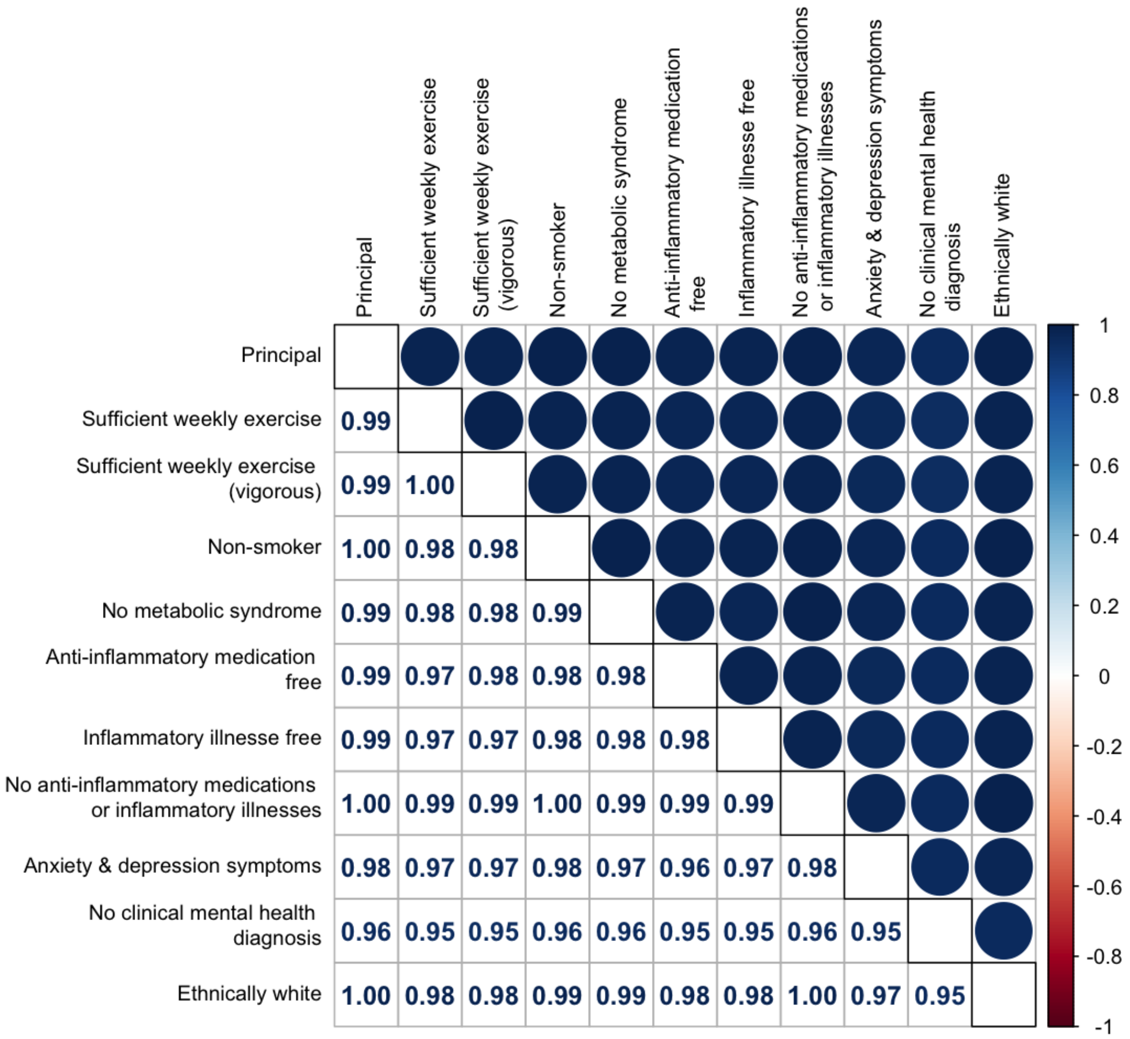

Figure SF11: Cross-sample correlation matrix for t-values derived from the (Brain $\sim$ CRP) linear regression model evaluated across 187 regions of the brain for the principal analysis and 10 sensitivity analyses to address specific potentially confounding variables. Columns and rows correspond to different sensitivity subsamples.

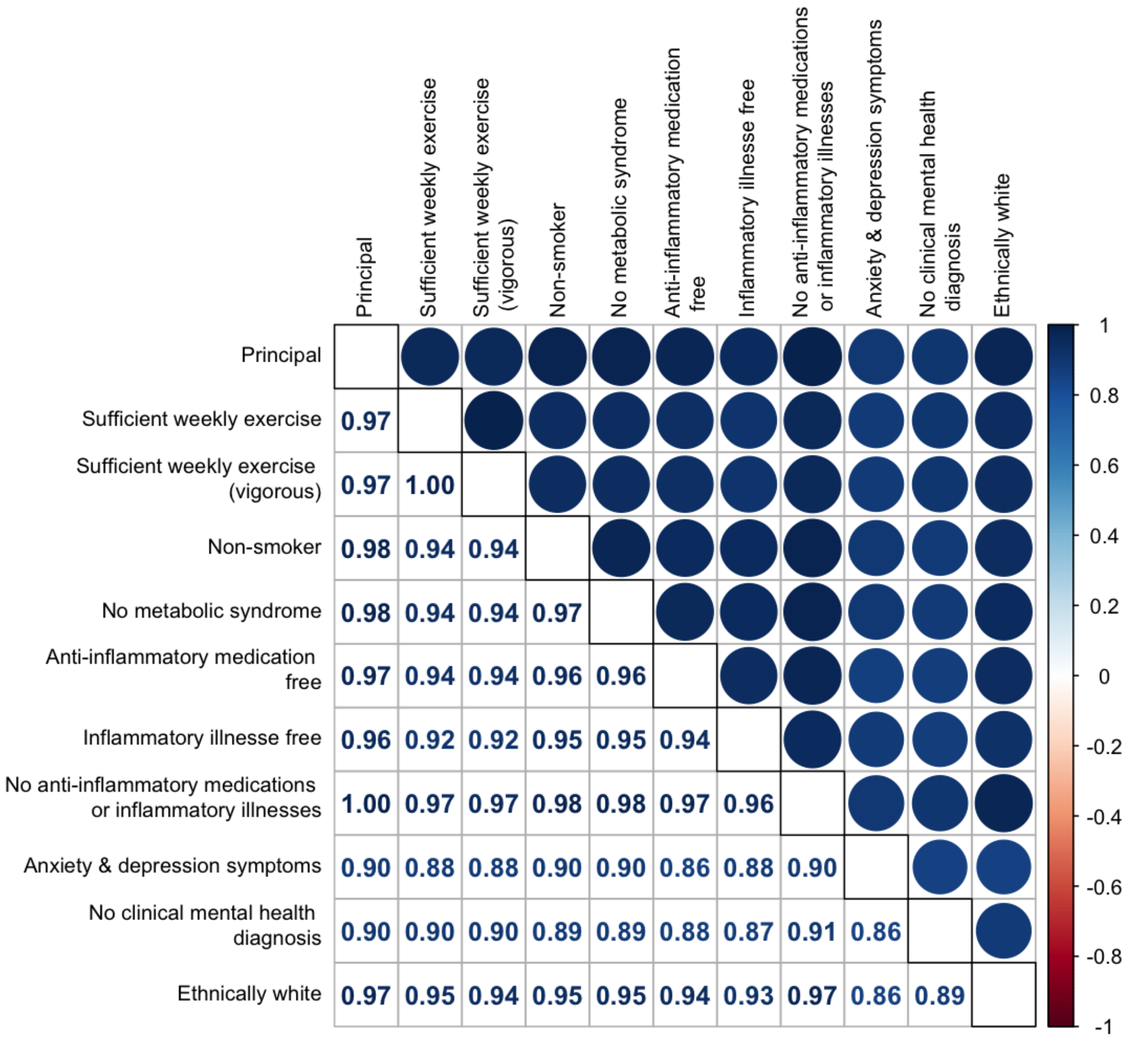

Figure SF12: Cross-sample correlation matrix for t-values derived from the (Brain $\sim$ AT) linear regression model evaluated across 187 regions of the brain for the principal analysis and 10 sensitivity analyses to address specific potentially confounding variables. Columns and rows correspond to different sensitivity subsamples.

SI 3.2 Sensitivity replication of H3

We repeated the entire analysis pipeline and verified our main results in relation to H3. Specifically, for all sensitivity subsamples derived from the sensitivity characteristics highlighted in Table ST1, as well the ethnicity-corrected sensitivity subsample (see SI 1.7), we aimed to formally replicate some of the elements in Figure 3 and correlate the results of each sensitivity analysis with those of the principal analysis. For each sensitivity analysis, we replicated the cortical map in Figure 3C, which depicts the regions where the full path model has a significantly better fit than the sparse model in yellow; and those regions where the sparse model has a significantly better fit than the full model in green (Figure SF13A). Next, for both the full and the sparse models, we selected the set of regions where model performance remained consistent across the principal analysis and the sensitivity analysis, such that we only evaluated in the next step regions that "remained" yellow or green in both the principal and sensitivity analyses (Figure SF13B). The percentage of regions that remained consistently yellow or green for each sensitivity analysis relative to the principal analyses can be found in Table ST3. We then evaluated the significant indirect paths of CM influence on brain structure for which we had estimated path coefficients in the full and sparse models, i.e., $a1 * c1$ and $a3 * c3$ for the full model, and $a1 * b1 * c2$ for the sparse model. For these paths, and in the consistently yellow or green regions defined previously, we estimated the z-values of indirect effects of CM on brain structure in multiple regions and correlated these results from the principal and sensitivity analyses. This allowed us to estimate the consistency of these hypothetically driven results after specific consideration of each potentially confounding variable (Figure SF13C).

Table ST3: Number of regions remaining consistent (overlapping) across principal and sensitivity analyses. Percentages in each column are expressed relative to the total number of regions identified as best fit by either a full ($n = 56$) or a sparse ($n = 124$) path model in the principal analysis; thus, a percentage indicates how consistent the results of the likelihood-ratio test of goodness-of-fit in a given sensitivity analysis were in contrast to the principal analysis.

**Subsample**	**Full N (%)**	**Sparse N (%)**
**Principal**	**56 (100%)**	**124 (100%)**
Sufficient weekly exercise	54 (96.43%)	122 (98.39%)
Sufficient weekly exercise (vigorous)	54 (96.43%)	122 (98.39%)
Non-smoker	49 (87.5%)	124 (100%)
No metabolic syndrome	55 (98.21%)	123 (99.19%)
Anti-inflammatory medication free	51 (91.07%)	123 (99.19%)
Inflammatory illness free	53 (94.64%)	122 (98.39%)
No anti-inflammatory medications or inflammatory illnesses	56 (100%)	124 (100%)
Anxiety & depression symptoms	49 (87.5%)	122 (98.39%)
No clinical mental health diagnosis	53 (94.64%)	122 (98.39%)
Ethnically white only	52 (92.86%)	124 (100%)

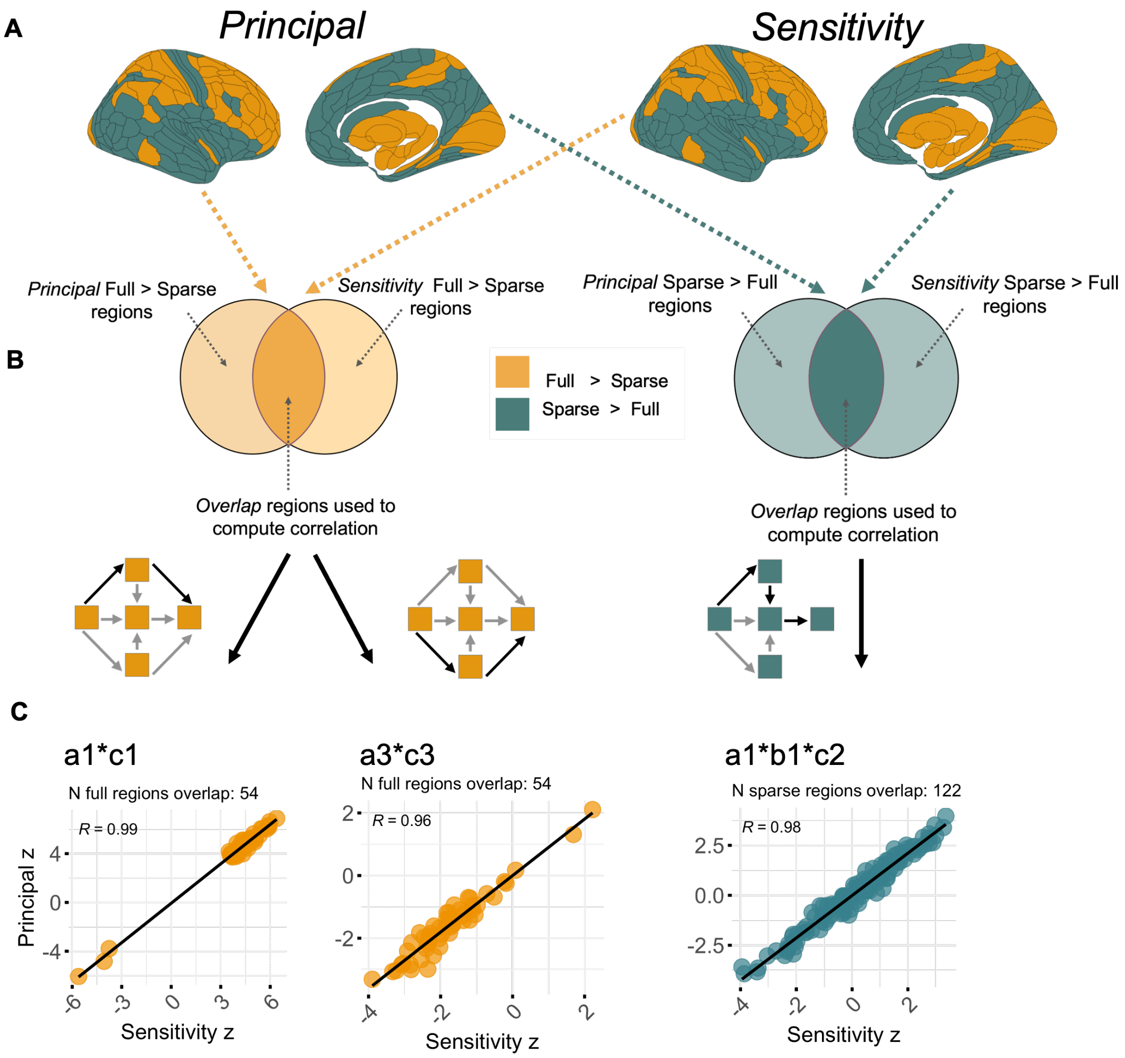

Figure SF13: Diagram of procedure used to evaluate the consistency between the principal analysis and multiple independent sensitivity analyses of the indirect effects of CM on brain structure (H3). **A.** First step: replication of the cortical map in Figure 3C for both the principal and the sensitivity analysis. This map shows those regions where the full model (Fig. 3A) has a significantly better fit than the sparse model as determined by a likelihood-ratio test (Fig. 3D), **in yellow**; and those regions where the sparse model has the better fit **in green**. **B.** Second step: the subset of regions where the full (or sparse) model consistently had the better fit across both principal and sensitivity analyses was selected. **C.** Third step: for each region where the full or sparse model consistently provided a better fit, indirect path coefficients were estimated for each of the paths shown to mediate significant indirect effects of CM on brain structure in the principal analysis, i.e. paths $a1 * c1$, $a3 * c3$ for the full model and path $a1 * b1 * c2$ for the sparse (Figures 3 B&E). The size of indirect effects of CM on brain structure at each region was estimated by a z-score and the consistency of results between principal and sensitivity analyses was assessed in terms of the correlation between their corresponding regional z-vectors. Exemplary scatterplots for each indirect path where coefficients were estimated are shown in this panel. The number of regions overlapping as either full or sparse between the principal and sensitivity analyses are displayed atop each plot, indicating sensitivity-to-principal results consistency in the likelihood-ratio test of goodness-of-fit (see Table ST3). In the principal analyses the full model covers a lower number of brain regions (56 for the full vs 124 for the sparse) and therefore yellow scatterplots display less data points than green scatterplots. Note that the distribution of $a1 * c1$ values is polar due to the intersection of this path (i) only being tested in the small number of regions covered by the full model; (ii) having significant effects across all 56 regions of the full model, and thus no effects near zero.

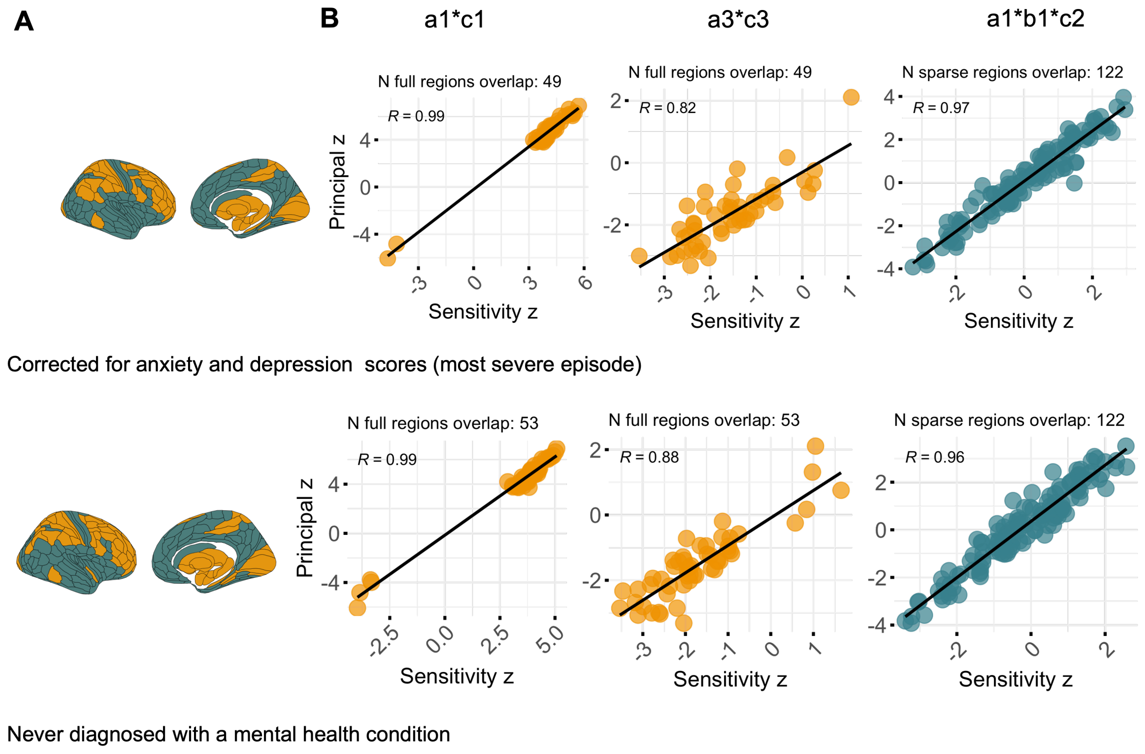

Figure SF14: Verification of H3: Sensitivity analyses of the effects of adult or lifetime mental health disorder diagnosis on indirect paths from CM to changes in brain regional MRI metrics.
This figure formally replicates some of the elements in Figure 3 of the main text, but after controlling for possible effects of adult mental health status in two ways. **Top row**: Results after adding depression and anxiety scores as covariates to the regression model used to residualise CM, AT, BMI, CRP and MRI metrics prior to path analysis. **Bottom row:** Results after excluding individuals ever diagnosed with a mental health disorder. (**A panels**) Cortical maps of regions where the full path model provided a significantly better fit than the sparse model (yellow) or did not provide a superior fit compared to the sparse model (green). This pair of nested models was evaluated at each of 180 cortical areas and 6 subcortical structures defined by the Glasser parcellation, and the likelihood-ratio statistic was used as a measure of relative goodness-of-fit. The maps are very similar but not identical: N=X regions (Y%) were consistently yellow or green in both maps. (**B panels**) Scatterplots of indirectly mediated effects of childhood maltreatment on brain structure (Wald Z) for the principal analysis (y-axis; data corrected for age, sex, and SES) vs the sensitivity analysis (x-axis; data additionally corrected for anxiety and depression scores). Paths corresponding to the full model are plotted in yellow; paths for the sparse model are plotted in green. **Left**: scatterplot of principal vs sensitivity analyses of the BMI-mediated path from CM to brain structure ($CM \rightarrow BMI \rightarrow Brain$; the product of path coefficients $a1 * c1$); **Centre**: scatterplot of principal vs sensitivity analyses of the AT-mediated path from CM to brain structure ($CM \rightarrow AT \rightarrow Brain$; the product of path coefficients $a3 * c3$); **Right**: scatterplot of the principal vs sensitivity analyses of the BMI-and-CRP-mediated path from CM to brain structure ($CM \rightarrow BMI \rightarrow CRP \rightarrow Brain$; the product of path coefficients $a1 * b1 * c2$). We found that the direction and magnitude of effects across the three paths were highly correlated (minimum $r = 0.82$), with the results of the principal analysis for both mental health-related sensitivity analyses, showing that the relationships between CM, AT, BMI, CRP and MRI metrics were not substantially confounded by adult mental health disorder.

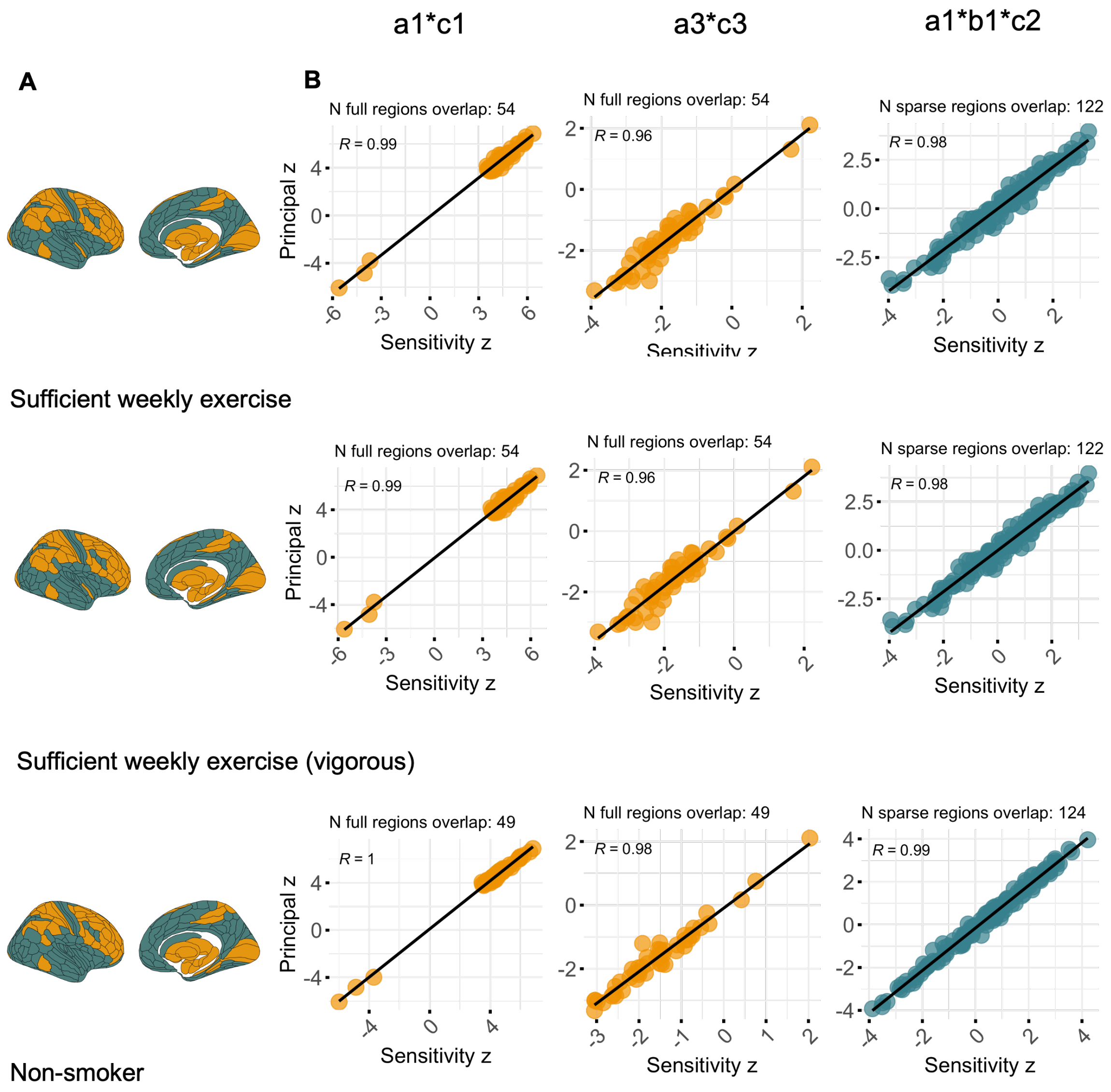

Figure SF15: Verification of H3: Sensitivity analyses of the effects lifestyle factors on indirect paths from CM to changes in brain regional MRI metrics. Plots are as described in figure SF14. Samples displayed are those considering different lifestyle factors and consist of (1) subjects who perform sufficient exercise weekly; (2) subjects who perform sufficient and/or vigorous exercise weekly; (3) subjects who do not smoke.

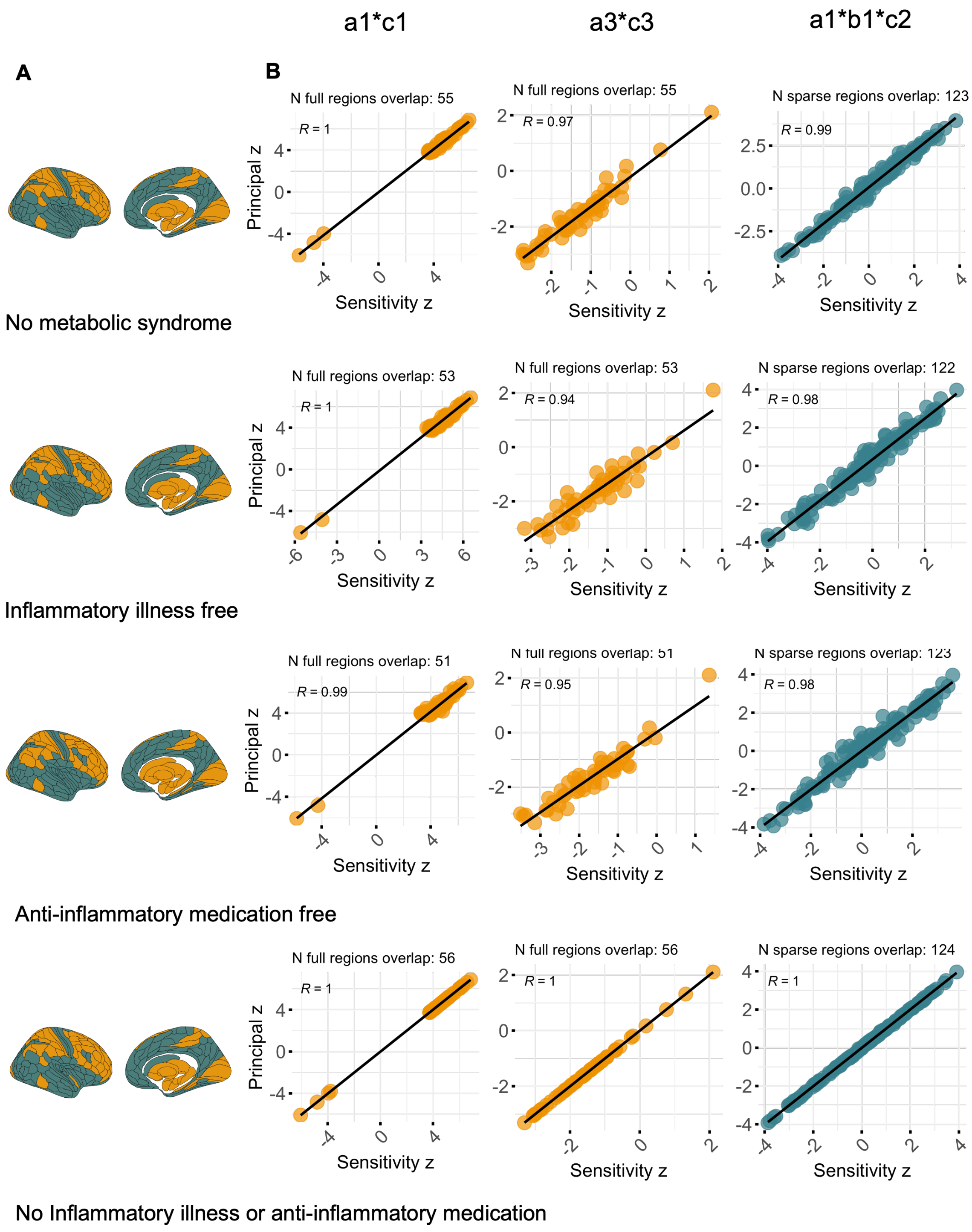

Figure SF16: Verification of H3: Sensitivity analyses of the effects of different physical health factors on indirect paths from CM to changes in brain regional MRI metrics Plots are as described in figure SF14. Samples displayed are those considering different physical health factors and consist of (1) subjects with no metabolic syndrome; (2) subjects who report no inflammatory illnesses; (3) subjects who take no anti-inflammatory medication; (4) subjects who do not report any inflammatory illnesses or take no anti-inflammatory medication

SI 3.3 Sensitivity replication of H3 considering ethnicity

We provided additional sample characteristics of self-reported ethnicity in the UKB MRI subsample. The percentage of non-white individuals in the sample was too small for a meaningful analysis of ethnic differences, see Table ST2. Therefore, a new subsample of comprised by only ethnically-white individuals was built and used to perform sensitivity analyses repeating the entire analysis pipeline and verifying the results for H3 according to the procedure outlined in Figure SF13.

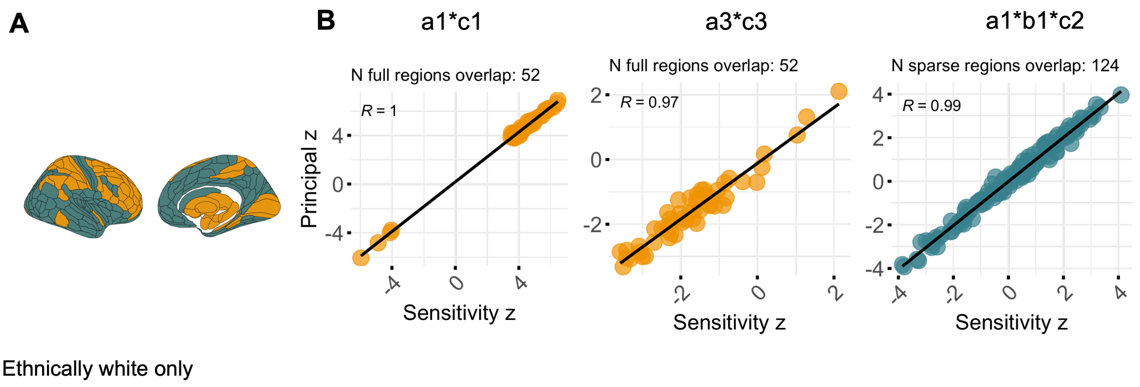

Figure SF17: Verification of H3: Sensitivity analyses replicating key findings of indirect effects of CM on MRI metrics in the subsample of only white individuals. Plots are as described in figure SF14.

SI 4 Supplementary Figures

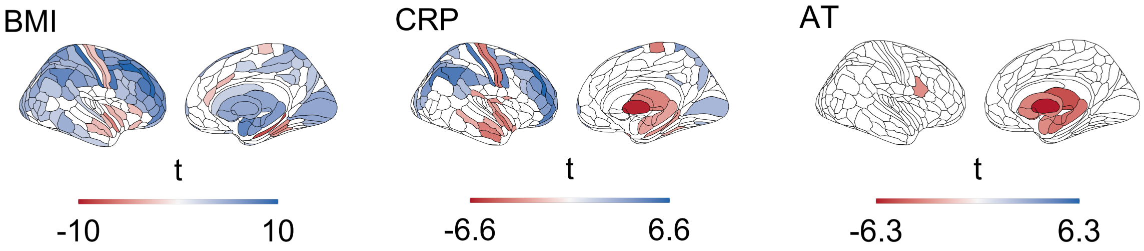

Figure SF18: Thresholded brain maps of independent linear relationships between adult trauma (AT), CRP or BMI and cortical thickness and subcortical volume. Each map shows the anatomical distribution of thresholded effects at FDR ≤ 0.05 effects (t values)

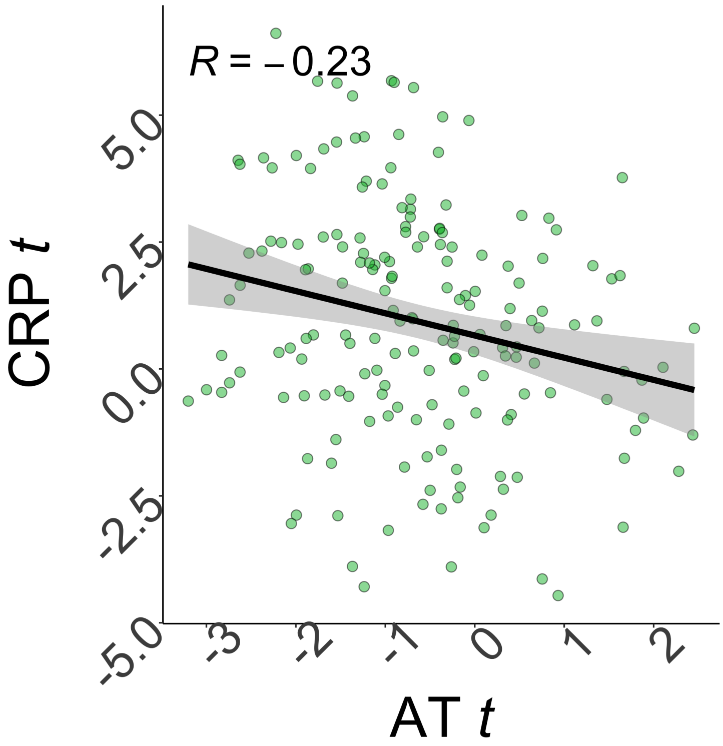

Figure SF19: Correlation of adult trauma and BMI effects on cortical thickness. Scatterplot of effect (t-value) of CRP (y-axis) versus effect of AT (x-axis) on cortical thickness; each point represents one of 180 cortical areas. Spearman's correlation $\rho = -0.23$ over all areas, solid line is the regression of t_{CRP} on t_{AT}

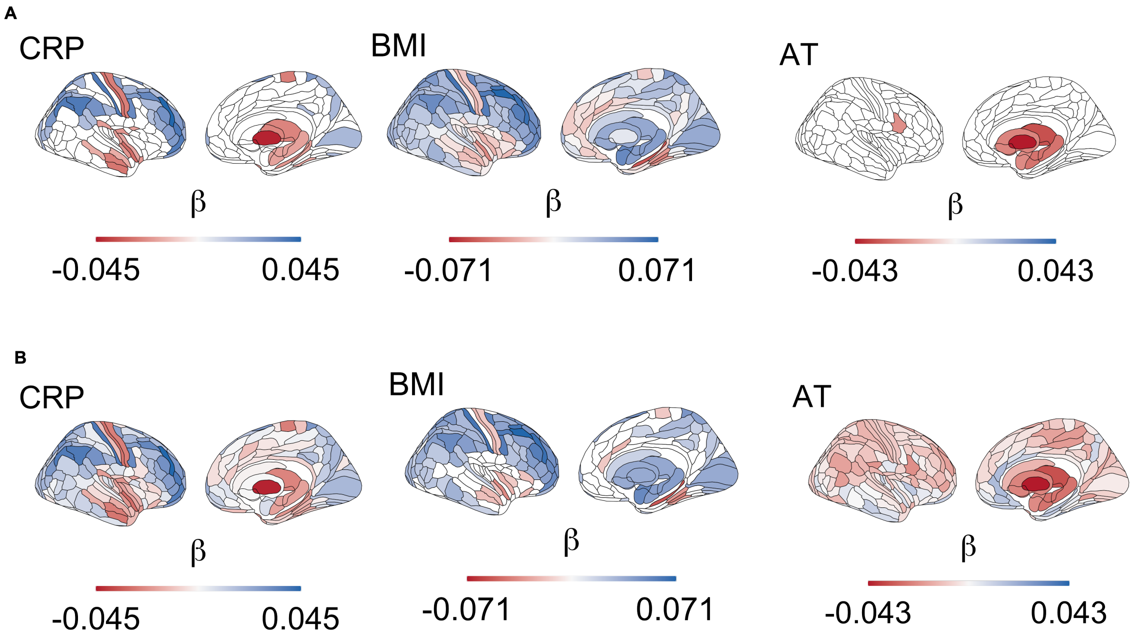

Figure SF20: Brain maps of independent linear relationships between adult trauma (AT), CRP or BMI and cortical thickness and subcortical volume. A Each map shows the anatomical distribution of unthresholded linear regression β coefficients. **B** β maps thresholded for FDR$<$ 0.05 corrected significance.

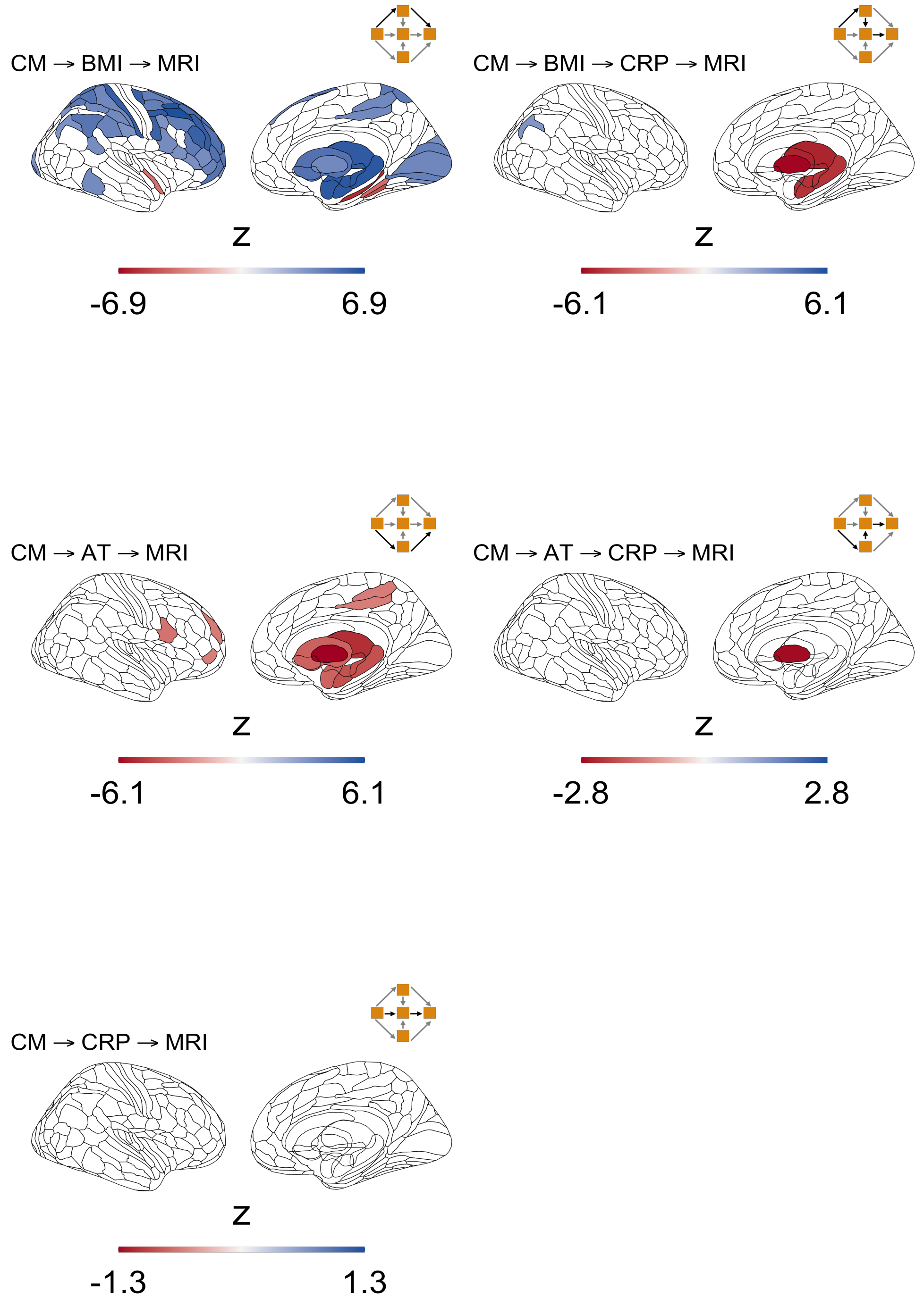

Figure SF21: Thresholded indirect (z) effects of childhood maltreatment on brain structure for all paths in the full model.

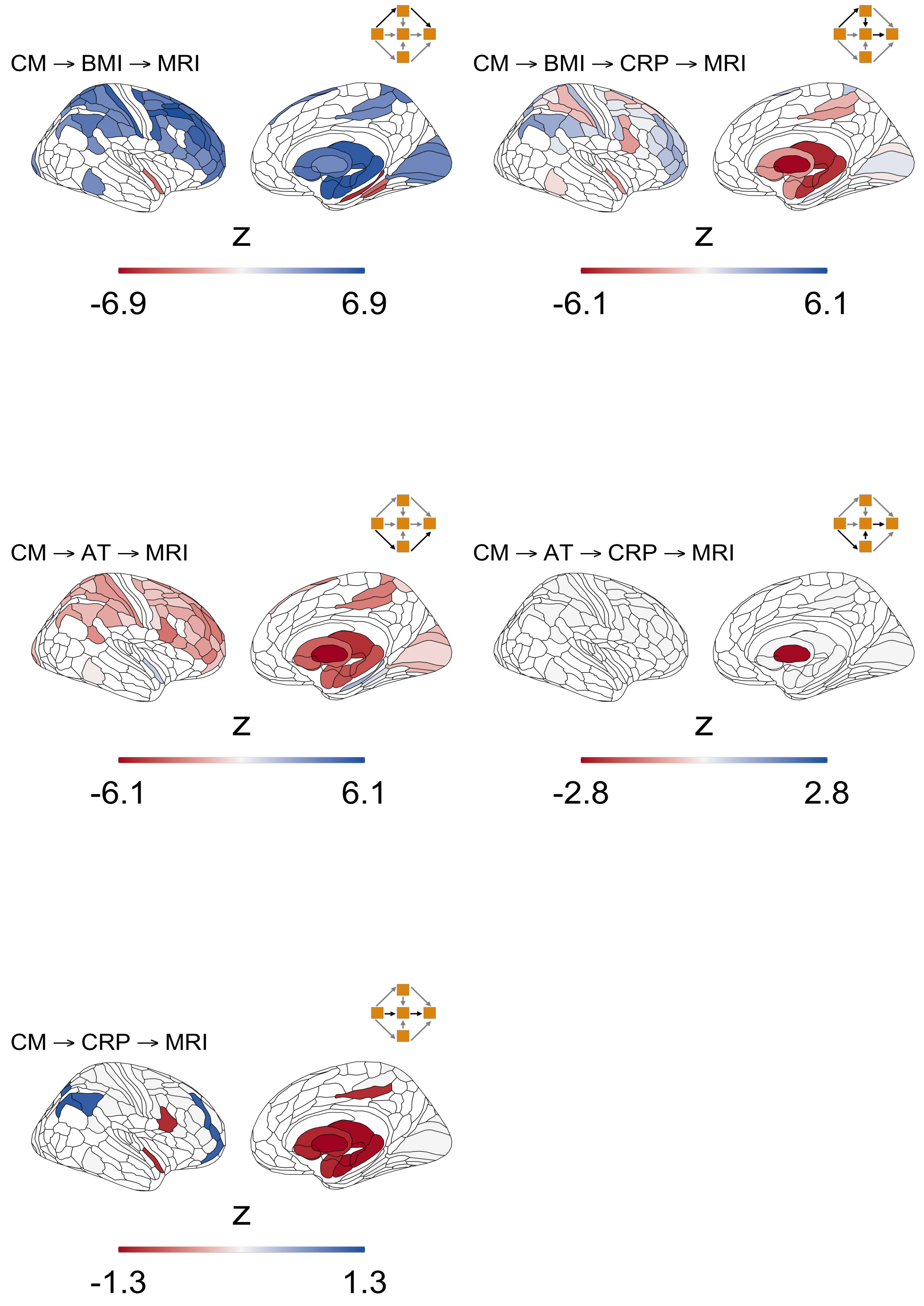

Figure SF22: Unthresholded indirect (z) effects of childhood maltreatment on brain structure for all paths in the full model.

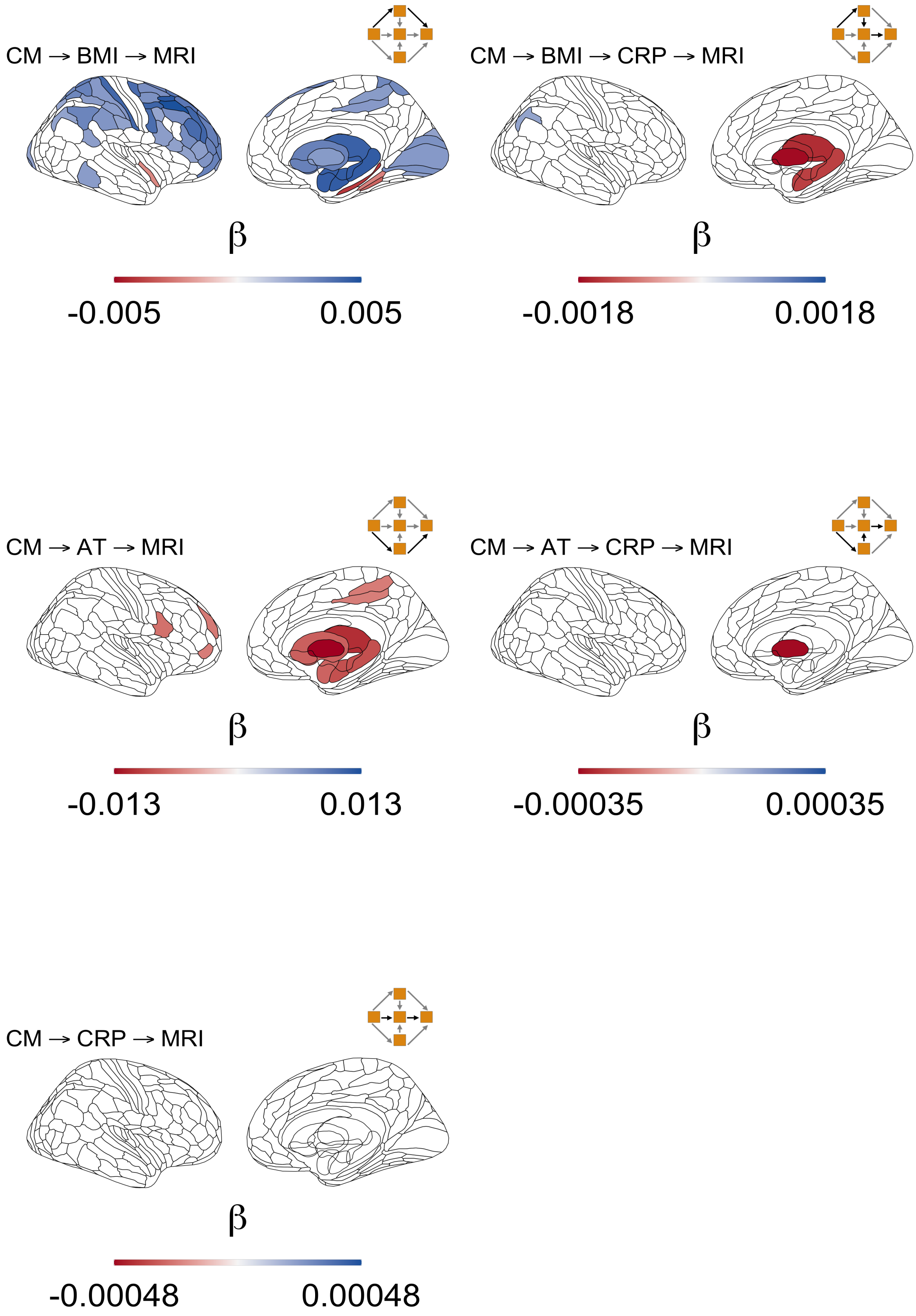

Figure SF23: Thresholded unstandardised indirect (β) effects of childhood maltreatment on brain structure for all paths in the full model.

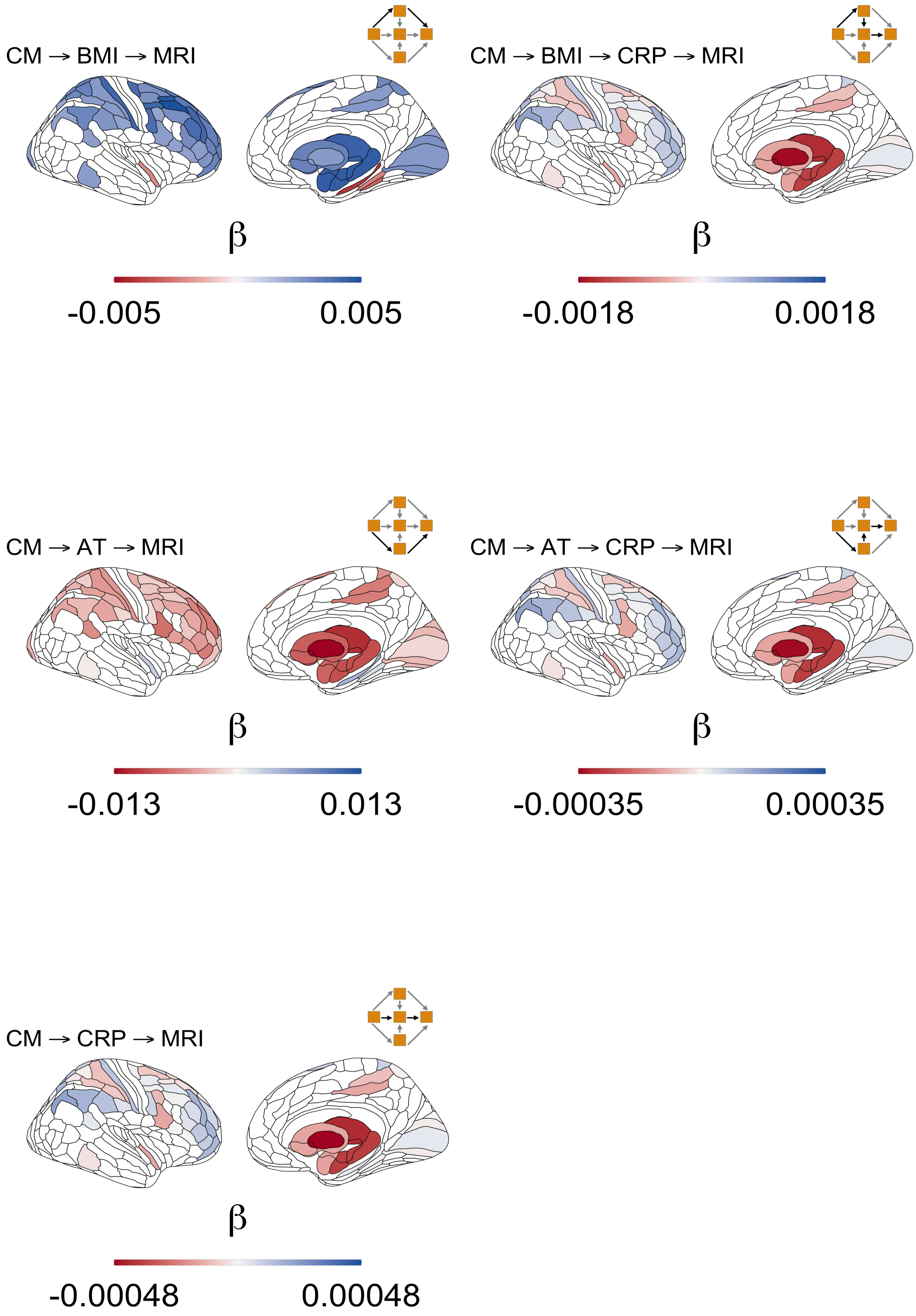

Figure SF24: Unthresholded unstandardised indirect (β) effects of childhood maltreatment on brain structure for all paths in the full model.

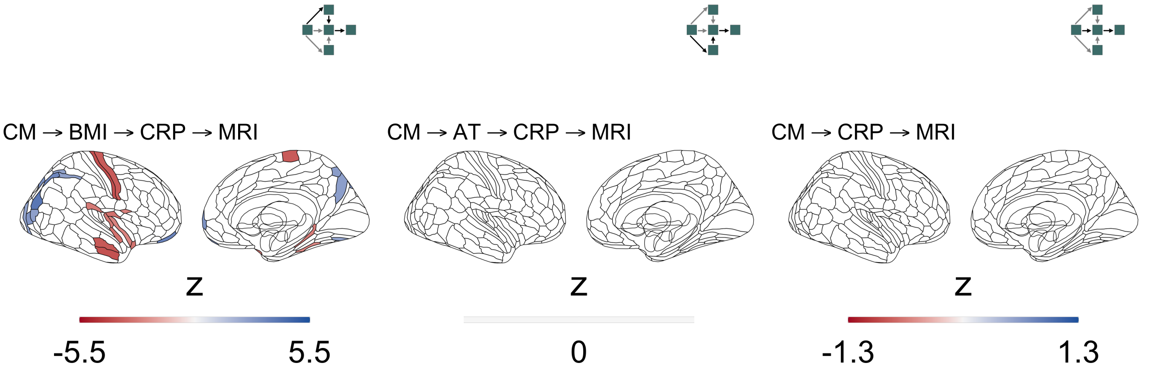

Figure SF25: Thresholded indirect (z) effects of childhood maltreatment on brain structure for all paths in the sparse model.

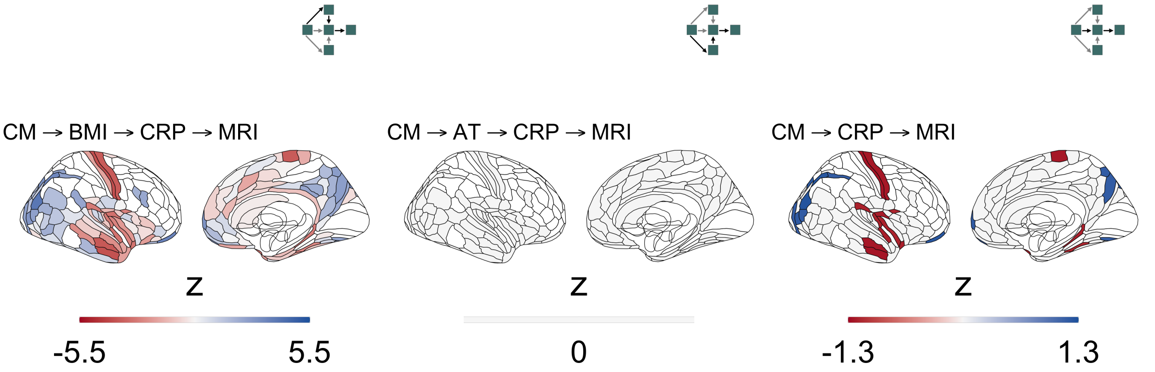

Figure SF26: Unthresholded indirect (z) effects of childhood maltreatment on brain structure for all paths in the sparse model.

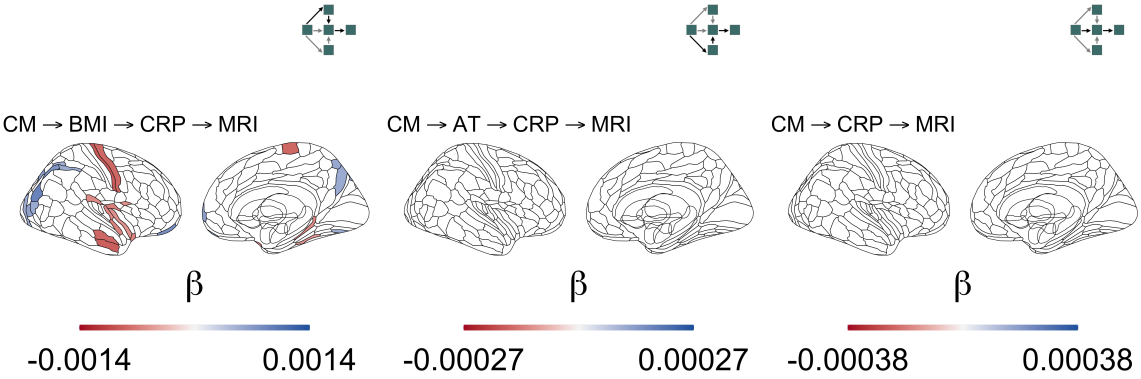

Figure SF27: Thresholded unstandardised indirect (β) effects of childhood maltreatment on brain structure for all paths in the sparse model.

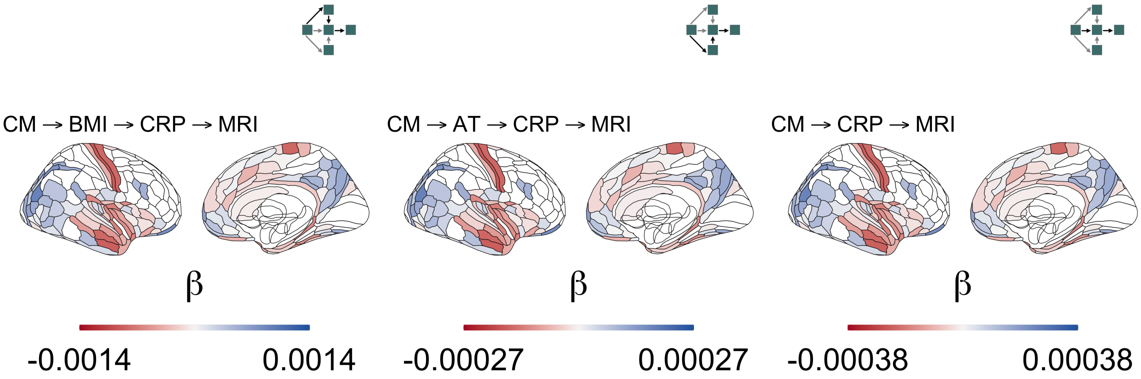

Figure SF28: Unthresholded unstandardised indirect (β) effects of childhood maltreatment on brain structure for all paths in the sparse model.

SI 5 Supplementary Tables

Table ST4: Log transformed values of clinical data on UKB sample and sub-sample with MRI data available. Mean and standard deviation (in brackets) are reported for each variable, unless otherwise specified.

	**UKB MRI sub-sample**	**95% C**	**UKB sample**	**95% CI**
N (%)	N = 21,738	-	N = 116,887	-
Female (%)	11,684 (54%)	-	65,715 (56%)	-
Body Mass Index, log	3.26 (0.15)	3.3, 3.3	3.28 (0.16)	3.3, 3.3
CRP, log	0.11 (1.02)	0.10, 0.12	0.21 (1.05)	0.20, 0.22
Childhood Maltreatment, log	0.72 (0.71)	0.71, 0.73	0.73 (0.72)	0.72, 0.73
Adult Trauma, log	0.79 (0.74)	0.78, 0.80	0.84 (0.75)	0.84, 0.85

Table ST5: Nuisance regression model results for immune, metabolic and psychosocial on UKB Imaging sample.

**nuisance variable**	β	**SE**	**t**	$P-$**value**
**CRP**				
deprivation	0.015	0.003	5.801	< 0.0001
age	0.018	0.001	13.436	< 0.0001
sex (male)	0.236	0.104	2.270	0.0232
age*sex (male)	-0.005	0.002	-2.691	0.0071
**BMI**				
deprivation	0.003	0.000	7.062	< 0.0001
age	0.001	0.000	7.588	< 0.0001
sex (male)	0.116	0.015	7.550	< 0.0001
age*sex (male)	-0.001	0.000	-4.808	< 0.0001
**AT**				
deprivation	0.040	0.002	21.986	< 0.0001
age	-0.001	0.001	-0.775	0.4383
sex (male)	-0.047	0.074	-0.631	0.5279
age*sex (male)	-0.003	0.001	-1.867	0.0619
**CM**				
deprivation	0.027	0.002	14.820	< 0.0001
age	-0.001	0.001	-1.182	0.2372
sex (male)	0.031	0.072	0.436	0.6629
age*sex (male)	-0.001	0.001	-0.846	0.3977

**nuisance variable**	β	**SE**	**t**	$P-$**value**
**CRP**				
deprivation	0.013	0.001	12.390	< 0.0001
age	0.018	0.001	33.818	< 0.0001
sex (male)	0.181	0.045	4.051	< 0.0001
age*sex (male)	-0.004	0.001	-5.231	< 0.0001
**BMI**				
deprivation	0.003	0.000	17.126	< 0.0001
age	0.001	0.000	17.751	< 0.0001
sex (male)	0.083	0.007	12.005	< 0.0001
age*sex (male)	-0.001	0.000	-6.235	< 0.0001
**AT**				
deprivation	0.040	0.001	51.727	0.0000
age	-0.003	0.000	-7.290	< 0.0001
sex (male)	-0.145	0.032	-4.562	< 0.0001
age*sex (male)	-0.001	0.001	-0.920	0.3574
**CM**				
deprivation	0.023	0.001	31.490	< 0.0001
age	-0.003	0.000	-8.481	< 0.0001
sex (male)	-0.135	0.031	-4.347	< 0.0001
age*sex (male)	0.002	0.001	3.763	0.0002

Table ST6: Nuisance regression model results for immune, metabolic and psychosocial on the larger UKB sample.

Table ST7: Path model coefficients for the relationships between childhood maltreatment, adult trauma, BMI and CRP in the UKB MRI sample.

Relationship	Predictor	Outcome	Path	β	SE	Z	$P-$value	95% CI
Direct	CM	BMI	a1	0.0721	0.0070	10.3433	0.0000	0.0584, 0.0857
Direct	CM	CRP	a2	0.0084	0.0064	1.3130	0.1892	-0.0042, 0.0210
Direct	CM	AT	a3	0.3155	0.0065	48.8822	0.0000	0.3028, 0.3281
Direct	BMI	CRP	b1	0.4340	0.0060	72.0471	0.0000	0.4222, 0.4458
Direct	AT	CRP	b2	0.0193	0.0064	2.9991	0.0027	0.0067, 0.0319
Indirect	CM	BMI→CRP	a1*b1	0.0313	0.0030	10.2570	0.0000	0.0253, 0.0373
Indirect	CM	AT→CRP	a3*b2	0.0061	0.0020	2.9896	0.0028	0.0021, 0.0101

Table ST8: Path model coefficients for the relationships between childhood maltreatment, adult trauma, BMI and CRP in the larger UKB sample.

**Relationship**	**Predictor**	**Outcome**	**Path**	β	**SE**	**Z**	P**−value**	**95% CI**
Direct	CM	BMI	a1	0.0654	0.0030	21.5850	0.0000	0.0595, 0.0713
Direct	CM	CRP	a2	0.0095	0.0028	3.4307	0.0006	0.0041, 0.0150
Direct	CM	AT	a3	0.3289	0.0027	120.8475	0.0000	0.3235, 0.3342
Direct	BMI	CRP	b1	0.4531	0.0026	176.8608	0.0000	0.4481, 0.4581
Direct	AT	CRP	b2	0.0214	0.0028	7.7605	<0.0001	0.0160, 0.0268
Indirect	CM	BMI→CRP	a1*b1	0.0296	0.0014	21.3995	0.0000	0.0269, 0.0323
Indirect	CM	AT→CRP	a3*b2	0.0070	0.0009	7.7408	<0.0001	0.0053, 0.0088

Table ST9: linear regression results at each brain area with BMI as the dependent variable and cortical thickness or subcortical volume as an independent variable

Brain area	Layer	β	SE	t-value	P_{FDR}
Thalamus-Proper	Subcortex	0.03685	0.00678	5.43670	0.00000
Caudate	Subcortex	0.01711	0.00678	2.52237	0.02038
Putamen	Subcortex	0.03591	0.00678	5.29760	0.00000
Pallidum	Subcortex	0.00733	0.00678	1.08074	0.36339
Hippocampus	Subcortex	0.04191	0.00678	6.18406	0.00000
Amygdala	Subcortex	0.05266	0.00677	7.77494	0.00000
Accumbens-area	Subcortex	0.01885	0.00678	2.77992	0.01105
V1	Cortex	0.03622	0.00678	5.34374	0.00000
MST	Cortex	0.02052	0.00678	3.02614	0.00533
V6	Cortex	0.00690	0.00678	1.01782	0.39280
V2	Cortex	0.03669	0.00678	5.41238	0.00000
V3	Cortex	0.03090	0.00678	4.55814	0.00002
V4	Cortex	0.02672	0.00678	3.94009	0.00023
V8	Cortex	0.02199	0.00678	3.24315	0.00284
4	Cortex	-0.01570	0.00678	-2.31427	0.03450
3b	Cortex	-0.00433	0.00678	-0.63897	0.59983
FEF	Cortex	0.03875	0.00678	5.71652	0.00000
PEF	Cortex	0.02370	0.00678	3.49482	0.00123
55b	Cortex	0.05914	0.00677	8.73474	0.00000
V3A	Cortex	0.01848	0.00678	2.72452	0.01255
RSC	Cortex	0.00876	0.00678	1.29086	0.27256
POS2	Cortex	0.02122	0.00678	3.12861	0.00392
V7	Cortex	0.01863	0.00678	2.74737	0.01196
IPS1	Cortex	0.02757	0.00678	4.06651	0.00014
FFC	Cortex	0.00315	0.00678	0.46453	0.69828
V3B	Cortex	0.01505	0.00678	2.21860	0.04351
LO1	Cortex	0.03058	0.00678	4.51001	0.00002
LO2	Cortex	0.00678	0.00678	1.00015	0.40085
PIT	Cortex	0.00540	0.00678	0.79561	0.50521
MT	Cortex	0.02255	0.00678	3.32489	0.00215
A1	Cortex	0.00709	0.00678	1.04474	0.37932
PSL	Cortex	0.02154	0.00678	3.17581	0.00341
SFL	Cortex	0.03525	0.00678	5.19976	0.00000
PCV	Cortex	0.01783	0.00678	2.62865	0.01588
STV	Cortex	0.03329	0.00678	4.91126	0.00000
7Pm	Cortex	0.02186	0.00678	3.22397	0.00300
7m	Cortex	-0.00178	0.00678	-0.26220	0.82402
POS1	Cortex	0.00206	0.00678	0.30417	0.79948
23d	Cortex	0.00860	0.00678	1.26832	0.28146
v23ab	Cortex	-0.00791	0.00678	-1.16582	0.32551
d23ab	Cortex	0.01148	0.00678	1.69205	0.13782
31pv	Cortex	0.02118	0.00678	3.12391	0.00393
5m	Cortex	0.00275	0.00678	0.40472	0.73271
5mv	Cortex	0.02762	0.00678	4.07366	0.00014
23c	Cortex	0.02597	0.00678	3.83042	0.00035
5L	Cortex	0.04619	0.00678	6.81744	0.00000

24dd	Cortex	0.00795	0.00678	1.17179	0.32462
24dv	Cortex	0.01592	0.00678	2.34742	0.03215
7AL	Cortex	0.05007	0.00677	7.39063	0.00000
SCEF	Cortex	0.00937	0.00678	1.38186	0.23663
6ma	Cortex	0.04741	0.00678	6.99769	0.00000
7Am	Cortex	0.04041	0.00678	5.96244	0.00000
7PL	Cortex	0.04959	0.00677	7.32067	0.00000
7PC	Cortex	0.04397	0.00678	6.48832	0.00000
LIPv	Cortex	0.04110	0.00678	6.06512	0.00000
VIP	Cortex	0.04692	0.00678	6.92523	0.00000
MIP	Cortex	0.03751	0.00678	5.53443	0.00000
1	Cortex	0.06309	0.00677	9.32011	0.00000
2	Cortex	0.02863	0.00678	4.22320	0.00007
3a	Cortex	-0.02127	0.00678	-3.13634	0.00386
6d	Cortex	0.05524	0.00677	8.15707	0.00000
6mp	Cortex	0.01010	0.00678	1.48891	0.20262
6v	Cortex	0.05848	0.00677	8.63691	0.00000
p24pr	Cortex	-0.00960	0.00678	-1.41484	0.22778
33pr	Cortex	-0.00903	0.00678	-1.33139	0.25741
a24pr	Cortex	-0.02111	0.00678	-3.11280	0.00403
p32pr	Cortex	-0.00309	0.00678	-0.45618	0.70073
a24	Cortex	-0.00941	0.00678	-1.38735	0.23603
d32	Cortex	-0.00713	0.00678	-1.05162	0.37785
8BM	Cortex	0.00074	0.00678	0.10951	0.91280
p32	Cortex	-0.00113	0.00678	-0.16588	0.88381
10r	Cortex	0.00884	0.00678	1.30310	0.26871
47m	Cortex	-0.00778	0.00678	-1.14636	0.33376
8Av	Cortex	0.07074	0.00677	10.45591	0.00000
8Ad	Cortex	0.04686	0.00678	6.91653	0.00000
9m	Cortex	-0.01409	0.00678	-2.07681	0.06099
8BL	Cortex	0.03062	0.00678	4.51598	0.00002
9p	Cortex	0.04178	0.00678	6.16462	0.00000
10d	Cortex	0.02702	0.00678	3.98448	0.00019
8C	Cortex	0.03945	0.00678	5.82069	0.00000
44	Cortex	0.03078	0.00678	4.54052	0.00002
45	Cortex	0.03086	0.00678	4.55207	0.00002
47l	Cortex	0.00670	0.00678	0.98837	0.40535
a47r	Cortex	0.05252	0.00677	7.75406	0.00000
6r	Cortex	0.02418	0.00678	3.56611	0.00097
IFJa	Cortex	0.02375	0.00678	3.50176	0.00122
IFJp	Cortex	0.02170	0.00678	3.19980	0.00318
IFSp	Cortex	0.04242	0.00678	6.26015	0.00000
IFSa	Cortex	0.03789	0.00678	5.59010	0.00000
p9-46v	Cortex	0.05838	0.00677	8.62206	0.00000
46	Cortex	0.06627	0.00677	9.79245	0.00000
a9-46v	Cortex	0.05300	0.00677	7.82470	0.00000
9-46d	Cortex	0.04880	0.00677	7.20388	0.00000
9a	Cortex	0.04572	0.00678	6.74707	0.00000
10v	Cortex	0.00451	0.00678	0.66539	0.58386
a10p	Cortex	0.03285	0.00678	4.84576	0.00000

10pp	Cortex	0.03288	0.00678	4.84963	0.00000
11l	Cortex	0.02824	0.00678	4.16501	0.00009
13l	Cortex	0.01415	0.00678	2.08617	0.06012
OFC	Cortex	0.00127	0.00678	0.18697	0.87509
47s	Cortex	-0.01991	0.00678	-2.93535	0.00693
LIPd	Cortex	0.02331	0.00678	3.43718	0.00151
6a	Cortex	0.03431	0.00678	5.06189	0.00000
i6-8	Cortex	0.06366	0.00677	9.40420	0.00000
s6-8	Cortex	0.04699	0.00678	6.93498	0.00000
43	Cortex	0.00650	0.00678	0.95819	0.41580
OP4	Cortex	0.00991	0.00678	1.46109	0.21204
OP1	Cortex	-0.00844	0.00678	-1.24430	0.29129
OP2-3	Cortex	-0.00459	0.00678	-0.67645	0.57931
52	Cortex	-0.00467	0.00678	-0.68911	0.57719
RI	Cortex	-0.00217	0.00678	-0.32022	0.79332
PFcm	Cortex	0.00601	0.00678	0.88681	0.45857
PoI2	Cortex	-0.02277	0.00678	-3.35736	0.00194
TA2	Cortex	-0.01849	0.00678	-2.72613	0.01255
FOP4	Cortex	-0.00084	0.00678	-0.12363	0.90645
MI	Cortex	-0.01520	0.00678	-2.24125	0.04140
Pir	Cortex	0.00401	0.00678	0.59127	0.62556
AVI	Cortex	-0.00566	0.00678	-0.83420	0.48849
AAIC	Cortex	-0.01307	0.00678	-1.92697	0.08415
FOP1	Cortex	-0.00652	0.00678	-0.96171	0.41580
FOP3	Cortex	-0.00105	0.00678	-0.15465	0.88658
FOP2	Cortex	-0.00345	0.00678	-0.50922	0.67564
PFt	Cortex	0.02894	0.00678	4.26850	0.00006
AIP	Cortex	0.02876	0.00678	4.24202	0.00007
EC	Cortex	-0.00956	0.00678	-1.40975	0.22818
PreS	Cortex	-0.01318	0.00678	-1.94353	0.08166
H	Cortex	-0.05471	0.00677	-8.07755	0.00000
ProS	Cortex	0.00182	0.00678	0.26889	0.82323
PeEc	Cortex	-0.00460	0.00678	-0.67885	0.57931
STGa	Cortex	-0.01096	0.00678	-1.61630	0.15864
PBelt	Cortex	-0.00539	0.00678	-0.79459	0.50521
A5	Cortex	0.00424	0.00678	0.62508	0.60652
PHA1	Cortex	-0.03259	0.00678	-4.80737	0.00001
PHA3	Cortex	-0.02975	0.00678	-4.38805	0.00004
STSda	Cortex	-0.01994	0.00678	-2.94075	0.00693
STSdp	Cortex	-0.00764	0.00678	-1.12622	0.34251
STSvp	Cortex	-0.00553	0.00678	-0.81539	0.49730
TGd	Cortex	-0.00296	0.00678	-0.43646	0.71200
TE1a	Cortex	-0.00743	0.00678	-1.09494	0.35772
TE1p	Cortex	0.03192	0.00678	4.70884	0.00001
TE2a	Cortex	0.01787	0.00678	2.63523	0.01573
TF	Cortex	-0.00380	0.00678	-0.55972	0.64078
TE2p	Cortex	0.01579	0.00678	2.32775	0.03358
PHT	Cortex	0.01840	0.00678	2.71270	0.01288
PH	Cortex	0.00565	0.00678	0.83292	0.48849
TPOJ1	Cortex	0.00384	0.00678	0.56600	0.63983

TPOJ2	Cortex	0.01239	0.00678	1.82751	0.10453
TPOJ3	Cortex	0.01729	0.00678	2.54956	0.01922
DVT	Cortex	0.01992	0.00678	2.93756	0.00693
PGp	Cortex	0.03430	0.00678	5.05928	0.00000
IP2	Cortex	0.02567	0.00678	3.78543	0.00042
IP1	Cortex	0.03184	0.00678	4.69595	0.00001
IP0	Cortex	0.01182	0.00678	1.74236	0.12486
PFop	Cortex	0.03309	0.00678	4.88152	0.00000
PF	Cortex	0.04230	0.00678	6.24190	0.00000
PFm	Cortex	0.05167	0.00677	7.62765	0.00000
PGi	Cortex	0.02172	0.00678	3.20312	0.00318
PGs	Cortex	0.04108	0.00678	6.06212	0.00000
V6A	Cortex	0.03015	0.00678	4.44733	0.00003
VMV1	Cortex	0.00324	0.00678	0.47723	0.69245
VMV3	Cortex	0.00215	0.00678	0.31747	0.79332
PHA2	Cortex	-0.04071	0.00678	-6.00650	0.00000
V4t	Cortex	0.01793	0.00678	2.64448	0.01547
FST	Cortex	0.02305	0.00678	3.39869	0.00170
V3CD	Cortex	0.01765	0.00678	2.60201	0.01685
LO3	Cortex	0.02304	0.00678	3.39789	0.00170
VMV2	Cortex	-0.00162	0.00678	-0.23810	0.83871
31pd	Cortex	0.01715	0.00678	2.52883	0.02020
31a	Cortex	0.00111	0.00678	0.16412	0.88381
VVC	Cortex	-0.00341	0.00678	-0.50257	0.67680
25	Cortex	-0.01125	0.00678	-1.65842	0.14665
s32	Cortex	-0.01323	0.00678	-1.95096	0.08094
pOFC	Cortex	0.01734	0.00678	2.55688	0.01900
PoI1	Cortex	-0.00656	0.00678	-0.96652	0.41580
Ig	Cortex	-0.01324	0.00678	-1.95270	0.08094
FOP5	Cortex	-0.01764	0.00678	-2.60173	0.01685
p10p	Cortex	0.04672	0.00678	6.89594	0.00000
p47r	Cortex	0.04122	0.00678	6.08200	0.00000
TGv	Cortex	0.01958	0.00678	2.88754	0.00799
MBelt	Cortex	0.00975	0.00678	1.43697	0.22022
LBelt	Cortex	-0.00400	0.00678	-0.58982	0.62556
A4	Cortex	0.01666	0.00678	2.45697	0.02427
STSva	Cortex	-0.01883	0.00678	-2.77684	0.01105
TE1m	Cortex	0.01817	0.00678	2.67953	0.01408
PI	Cortex	-0.03790	0.00678	-5.59189	0.00000
a32pr	Cortex	-0.00816	0.00678	-1.20293	0.31033
p24	Cortex	-0.01598	0.00678	-2.35588	0.03172

Table ST10: linear regression results at each brain area with CRP as the dependent variable and cortical thickness or subcortical volume as an independent variable

Brain area	Layer	β	SE	t-value	P_{FDR}
Thalamus-Proper	Subcortex	-0.02510	0.00678	-3.70189	0.00131
Caudate	Subcortex	-0.00140	0.00678	-0.20634	0.88881
Putamen	Subcortex	0.00031	0.00678	0.04633	0.97875
Pallidum	Subcortex	-0.04378	0.00678	-6.46063	0.00000
Hippocampus	Subcortex	-0.01871	0.00678	-2.75887	0.02116
Amygdala	Subcortex	0.00715	0.00678	1.05486	0.45425
Accumbens-area	Subcortex	-0.00986	0.00678	-1.45302	0.25320
V1	Cortex	0.01868	0.00678	2.75440	0.02116
MST	Cortex	0.00782	0.00678	1.15357	0.39747
V6	Cortex	-0.00348	0.00678	-0.51306	0.74313
V2	Cortex	0.01389	0.00678	2.04868	0.09017
V3	Cortex	0.01329	0.00678	1.95897	0.10406
V4	Cortex	0.01750	0.00678	2.57995	0.03082
V8	Cortex	0.01477	0.00678	2.17789	0.07146
4	Cortex	-0.02638	0.00678	-3.89051	0.00067
3b	Cortex	-0.01260	0.00678	-1.85681	0.12738
FEF	Cortex	0.01901	0.00678	2.80284	0.01975
PEF	Cortex	-0.00064	0.00678	-0.09478	0.95514
55b	Cortex	0.02687	0.00678	3.96339	0.00056
V3A	Cortex	0.00851	0.00678	1.25506	0.34360
RSC	Cortex	-0.00737	0.00678	-1.08597	0.43608
POS2	Cortex	0.01826	0.00678	2.69266	0.02457
V7	Cortex	0.01046	0.00678	1.54230	0.21702
IPS1	Cortex	0.02191	0.00678	3.23046	0.00661
FFC	Cortex	0.00284	0.00678	0.41908	0.77808
V3B	Cortex	0.01085	0.00678	1.59967	0.19535
LO1	Cortex	0.02014	0.00678	2.96967	0.01268
LO2	Cortex	0.00078	0.00678	0.11466	0.94405
PIT	Cortex	-0.00294	0.00678	-0.43325	0.77220
MT	Cortex	0.00128	0.00678	0.18817	0.89376
A1	Cortex	0.00642	0.00678	0.94695	0.50018
PSL	Cortex	0.01667	0.00678	2.45807	0.04084
SFL	Cortex	0.01434	0.00678	2.11403	0.07970
PCV	Cortex	0.01147	0.00678	1.69050	0.16674
STV	Cortex	0.01707	0.00678	2.51668	0.03575
7Pm	Cortex	0.01859	0.00678	2.74063	0.02165
7m	Cortex	0.01380	0.00678	2.03440	0.09223
POS1	Cortex	0.01211	0.00678	1.78594	0.14438
23d	Cortex	0.00408	0.00678	0.60208	0.69781
v23ab	Cortex	-0.00089	0.00678	-0.13152	0.93538
d23ab	Cortex	0.01630	0.00678	2.40320	0.04496
31pv	Cortex	0.01492	0.00678	2.19973	0.06849
5m	Cortex	-0.01086	0.00678	-1.60125	0.19535
5mv	Cortex	0.00181	0.00678	0.26650	0.86376
23c	Cortex	-0.00313	0.00678	-0.46123	0.76296
5L	Cortex	0.02851	0.00678	4.20448	0.00026

24dd	Cortex	-0.00042	0.00678	-0.06220	0.97651
24dv	Cortex	-0.00365	0.00678	-0.53813	0.73615
7AL	Cortex	0.02939	0.00678	4.33466	0.00017
SCEF	Cortex	0.00241	0.00678	0.35518	0.81879
6ma	Cortex	0.02820	0.00678	4.15865	0.00030
7Am	Cortex	0.01662	0.00678	2.45025	0.04109
7PL	Cortex	0.03646	0.00678	5.37942	0.00000
7PC	Cortex	0.01766	0.00678	2.60417	0.02961
LIPv	Cortex	0.01543	0.00678	2.27566	0.05781
VIP	Cortex	0.03132	0.00678	4.61966	0.00007
MIP	Cortex	0.03101	0.00678	4.57360	0.00008
1	Cortex	0.03842	0.00678	5.66841	0.00000
2	Cortex	0.00280	0.00678	0.41218	0.77808
3a	Cortex	-0.02909	0.00678	-4.28998	0.00020
6d	Cortex	0.02508	0.00678	3.69930	0.00131
6mp	Cortex	-0.00359	0.00678	-0.52859	0.73945
6v	Cortex	0.03318	0.00678	4.89420	0.00002
p24pr	Cortex	-0.00478	0.00678	-0.70456	0.64722
33pr	Cortex	-0.00407	0.00678	-0.59995	0.69781
a24pr	Cortex	-0.00319	0.00678	-0.46973	0.76057
p32pr	Cortex	-0.01174	0.00678	-1.73089	0.15612
a24	Cortex	0.00543	0.00678	0.80098	0.57759
d32	Cortex	-0.00221	0.00678	-0.32587	0.82930
8BM	Cortex	-0.00020	0.00678	-0.02923	0.97992
p32	Cortex	0.00348	0.00678	0.51292	0.74313
10r	Cortex	0.01147	0.00678	1.69142	0.16674
47m	Cortex	-0.00295	0.00678	-0.43554	0.77220
8Av	Cortex	0.03367	0.00678	4.96698	0.00001
8Ad	Cortex	0.01631	0.00678	2.40425	0.04496
9m	Cortex	-0.00511	0.00678	-0.75359	0.61128
8BL	Cortex	0.01238	0.00678	1.82579	0.13365
9p	Cortex	0.01548	0.00678	2.28195	0.05764
10d	Cortex	0.02134	0.00678	3.14638	0.00815
8C	Cortex	0.01796	0.00678	2.64804	0.02658
44	Cortex	0.01337	0.00678	1.97194	0.10218
45	Cortex	0.01326	0.00678	1.95465	0.10406
47l	Cortex	0.00579	0.00678	0.85321	0.54515
a47r	Cortex	0.03757	0.00678	5.54225	0.00000
6r	Cortex	-0.00429	0.00678	-0.63306	0.68398
IFJa	Cortex	0.01628	0.00678	2.40118	0.04496
IFJp	Cortex	0.01373	0.00678	2.02488	0.09327
IFSp	Cortex	0.02269	0.00678	3.34555	0.00452
IFSa	Cortex	0.02677	0.00678	3.94869	0.00057
p9-46v	Cortex	0.03084	0.00678	4.54877	0.00008
46	Cortex	0.04480	0.00678	6.61107	0.00000
a9-46v	Cortex	0.03848	0.00678	5.67716	0.00000
9-46d	Cortex	0.02787	0.00678	4.11076	0.00034
9a	Cortex	0.02686	0.00678	3.96178	0.00056
10v	Cortex	0.00807	0.00678	1.19017	0.37721
a10p	Cortex	0.01520	0.00678	2.24090	0.06244

10pp	Cortex	0.02050	0.00678	3.02366	0.01113
11l	Cortex	0.02556	0.00678	3.76887	0.00106
13l	Cortex	0.01204	0.00678	1.77505	0.14633
OFC	Cortex	-0.01194	0.00678	-1.75967	0.14824
47s	Cortex	-0.01342	0.00678	-1.97900	0.10164
LIPd	Cortex	0.01688	0.00678	2.48953	0.03799
6a	Cortex	0.00670	0.00678	0.98854	0.48306
i6-8	Cortex	0.02472	0.00678	3.64492	0.00157
s6-8	Cortex	0.01425	0.00678	2.10055	0.08139
43	Cortex	-0.00017	0.00678	-0.02517	0.97992
OP4	Cortex	0.00647	0.00678	0.95362	0.50018
OP1	Cortex	0.00436	0.00678	0.64350	0.67988
OP2-3	Cortex	-0.00943	0.00678	-1.39076	0.27933
52	Cortex	-0.01960	0.00678	-2.89073	0.01588
RI	Cortex	-0.01952	0.00678	-2.87908	0.01588
PFcm	Cortex	-0.00278	0.00678	-0.40923	0.77808
PoI2	Cortex	-0.01436	0.00678	-2.11754	0.07970
TA2	Cortex	-0.02121	0.00678	-3.12716	0.00847
FOP4	Cortex	-0.00381	0.00678	-0.56106	0.72135
MI	Cortex	-0.00149	0.00678	-0.21957	0.88794
Pir	Cortex	-0.02065	0.00678	-3.04461	0.01064
AVI	Cortex	-0.00334	0.00678	-0.49262	0.74594
AAIC	Cortex	0.00383	0.00678	0.56498	0.72135
FOP1	Cortex	-0.01869	0.00678	-2.75592	0.02116
FOP3	Cortex	0.00640	0.00678	0.94427	0.50018
FOP2	Cortex	-0.00628	0.00678	-0.92539	0.51033
PFt	Cortex	0.00453	0.00678	0.66759	0.66425
AIP	Cortex	0.00455	0.00678	0.67019	0.66425
EC	Cortex	-0.00821	0.00678	-1.21059	0.36760
PreS	Cortex	-0.01952	0.00678	-2.87914	0.01588
H	Cortex	-0.02114	0.00678	-3.11771	0.00853
ProS	Cortex	0.00132	0.00678	0.19507	0.89310
PeEc	Cortex	-0.00609	0.00678	-0.89819	0.52687
STGa	Cortex	-0.01199	0.00678	-1.76713	0.14735
PBelt	Cortex	-0.00701	0.00678	-1.03290	0.46620
A5	Cortex	-0.00338	0.00678	-0.49869	0.74559
PHA1	Cortex	-0.01447	0.00678	-2.13394	0.07878
PHA3	Cortex	-0.01721	0.00678	-2.53722	0.03428
STSda	Cortex	-0.01622	0.00678	-2.39126	0.04553
STSdp	Cortex	-0.00588	0.00678	-0.86678	0.54282
STSvp	Cortex	-0.01608	0.00678	-2.37029	0.04751
TGd	Cortex	-0.01311	0.00678	-1.93288	0.10827
TE1a	Cortex	-0.02805	0.00678	-4.13729	0.00031
TE1p	Cortex	0.01037	0.00678	1.52837	0.22097
TE2a	Cortex	0.00589	0.00678	0.86843	0.54282
TF	Cortex	-0.01367	0.00678	-2.01591	0.09419
TE2p	Cortex	0.00295	0.00678	0.43445	0.77220
PHT	Cortex	0.00772	0.00678	1.13787	0.40441
PH	Cortex	0.00408	0.00678	0.60084	0.69781
TPOJ1	Cortex	0.00342	0.00678	0.50365	0.74559

TPOJ2	Cortex	0.00923	0.00678	1.36014	0.29018
TPOJ3	Cortex	0.01416	0.00678	2.08758	0.08302
DVT	Cortex	0.01575	0.00678	2.32239	0.05252
PGp	Cortex	0.02893	0.00678	4.26706	0.00021
IP2	Cortex	0.01763	0.00678	2.59945	0.02961
IP1	Cortex	0.02430	0.00678	3.58343	0.00193
IP0	Cortex	0.00685	0.00678	1.01023	0.47856
PFop	Cortex	0.01878	0.00678	2.76961	0.02116
PF	Cortex	0.03030	0.00678	4.46957	0.00010
PFm	Cortex	0.03826	0.00678	5.64519	0.00000
PGi	Cortex	0.01118	0.00678	1.64843	0.18025
PGs	Cortex	0.03817	0.00678	5.63162	0.00000
V6A	Cortex	0.02030	0.00678	2.99326	0.01202
VMV1	Cortex	-0.00185	0.00678	-0.27295	0.86338
VMV3	Cortex	0.00549	0.00678	0.80959	0.57500
PHA2	Cortex	-0.01578	0.00678	-2.32703	0.05252
V4t	Cortex	0.00230	0.00678	0.33969	0.82696
FST	Cortex	0.01439	0.00678	2.12126	0.07970
V3CD	Cortex	0.00981	0.00678	1.44596	0.25426
LO3	Cortex	0.01822	0.00678	2.68627	0.02458
VMV2	Cortex	0.00461	0.00678	0.68033	0.66292
31pd	Cortex	0.00221	0.00678	0.32519	0.82930
31a	Cortex	0.00927	0.00678	1.36718	0.28906
VVC	Cortex	-0.00656	0.00678	-0.96711	0.49495
25	Cortex	-0.00033	0.00678	-0.04847	0.97875
s32	Cortex	0.00022	0.00678	0.03218	0.97992
pOFC	Cortex	0.00157	0.00678	0.23091	0.88353
PoI1	Cortex	-0.01809	0.00678	-2.66714	0.02556
Ig	Cortex	-0.02156	0.00678	-3.17896	0.00752
FOP5	Cortex	-0.00678	0.00678	-0.99960	0.47883
p10p	Cortex	0.02155	0.00678	3.17724	0.00752
p47r	Cortex	0.02734	0.00678	4.03254	0.00045
TGv	Cortex	0.00174	0.00678	0.25616	0.86741
MBelt	Cortex	-0.00682	0.00678	-1.00527	0.47856
LBelt	Cortex	0.00583	0.00678	0.85984	0.54409
A4	Cortex	0.00207	0.00678	0.30521	0.84118
STSva	Cortex	-0.02644	0.00678	-3.89910	0.00067
TE1m	Cortex	0.01247	0.00678	1.83826	0.13137
PI	Cortex	-0.03026	0.00678	-4.46342	0.00010
a32pr	Cortex	0.00144	0.00678	0.21235	0.88881
p24	Cortex	-0.00882	0.00678	-1.30075	0.31998

Table ST11: linear regression results at each brain area with AT as the dependent variable and cortical thickness or subcortical volume as an independent variable

Brain area	Layer	β	SE	t-value	P_{FDR}
Thalamus-Proper	Subcortex	-0.03434	0.00678	-5.06627	0.00004
Caudate	Subcortex	-0.01349	0.00678	-1.98851	0.25715
Putamen	Subcortex	-0.02407	0.00678	-3.54960	0.01446
Pallidum	Subcortex	-0.04276	0.00678	-6.31010	0.00000
Hippocampus	Subcortex	-0.02754	0.00678	-4.06238	0.00228
Amygdala	Subcortex	-0.02255	0.00678	-3.32470	0.02763
Accumbens-area	Subcortex	-0.03389	0.00678	-4.99936	0.00004
V1	Cortex	-0.00268	0.00678	-0.39545	0.87126
MST	Cortex	-0.00619	0.00678	-0.91218	0.65042
V6	Cortex	-0.01136	0.00678	-1.67556	0.34147
V2	Cortex	-0.00757	0.00678	-1.11619	0.56238
V3	Cortex	-0.00774	0.00678	-1.14115	0.55841
V4	Cortex	-0.00870	0.00678	-1.28274	0.49649
V8	Cortex	0.00516	0.00678	0.76012	0.71410
4	Cortex	-0.00927	0.00678	-1.36729	0.44680
3b	Cortex	-0.01087	0.00678	-1.60179	0.36470
FEF	Cortex	-0.00525	0.00678	-0.77366	0.71410
PEF	Cortex	-0.00834	0.00678	-1.22931	0.50552
55b	Cortex	-0.00636	0.00678	-0.93784	0.65042
V3A	Cortex	-0.00039	0.00678	-0.05764	0.96959
RSC	Cortex	-0.00196	0.00678	-0.28935	0.88295
POS2	Cortex	-0.00523	0.00678	-0.77153	0.71410
V7	Cortex	-0.00681	0.00678	-1.00386	0.62756
IPS1	Cortex	-0.00218	0.00678	-0.32098	0.87433
FFC	Cortex	0.00211	0.00678	0.31154	0.87433
V3B	Cortex	-0.00210	0.00678	-0.30895	0.87433
LO1	Cortex	0.00562	0.00678	0.82845	0.68639
LO2	Cortex	0.00452	0.00678	0.66653	0.74370
PIT	Cortex	-0.01022	0.00678	-1.50662	0.39158
MT	Cortex	-0.00152	0.00678	-0.22388	0.90514
A1	Cortex	0.00926	0.00678	1.36582	0.44680
PSL	Cortex	-0.01340	0.00678	-1.97633	0.25715
SFL	Cortex	-0.00646	0.00678	-0.95228	0.65042
PCV	Cortex	-0.01004	0.00678	-1.48012	0.39546
STV	Cortex	-0.01546	0.00678	-2.27965	0.18333
7Pm	Cortex	0.00619	0.00678	0.91210	0.65042
7m	Cortex	0.00896	0.00678	1.32046	0.47179
POS1	Cortex	-0.00631	0.00678	-0.93074	0.65042
23d	Cortex	-0.00758	0.00678	-1.11668	0.56238
v23ab	Cortex	0.00064	0.00678	0.09478	0.96044
d23ab	Cortex	-0.00435	0.00678	-0.64090	0.75447
31pv	Cortex	-0.00683	0.00678	-1.00731	0.62756
5m	Cortex	-0.00253	0.00678	-0.37312	0.87126
5mv	Cortex	-0.01919	0.00678	-2.82940	0.08734
23c	Cortex	-0.01919	0.00678	-2.82919	0.08734
5L	Cortex	-0.01353	0.00678	-1.99482	0.25715

24dd	Cortex	-0.01780	0.00678	-2.62438	0.10183
24dv	Cortex	-0.00956	0.00678	-1.40899	0.43685
7AL	Cortex	-0.01145	0.00678	-1.68809	0.34147
SCEF	Cortex	-0.00464	0.00678	-0.68450	0.73266
6ma	Cortex	-0.01601	0.00678	-2.36093	0.16240
7Am	Cortex	-0.00261	0.00678	-0.38468	0.87126
7PL	Cortex	-0.00926	0.00678	-1.36575	0.44680
7PC	Cortex	-0.00387	0.00678	-0.57090	0.80477
LIPv	Cortex	-0.00844	0.00678	-1.24381	0.50552
VIP	Cortex	-0.00576	0.00678	-0.84958	0.68203
MIP	Cortex	-0.00838	0.00678	-1.23564	0.50552
1	Cortex	-0.01192	0.00678	-1.75701	0.31404
2	Cortex	-0.01399	0.00678	-2.06310	0.24310
3a	Cortex	-0.00839	0.00678	-1.23635	0.50552
6d	Cortex	-0.00823	0.00678	-1.21284	0.51357
6mp	Cortex	-0.01293	0.00678	-1.90599	0.27270
6v	Cortex	-0.00045	0.00678	-0.06567	0.96959
p24pr	Cortex	-0.00324	0.00678	-0.47724	0.84687
33pr	Cortex	0.01002	0.00678	1.47744	0.39546
a24pr	Cortex	0.00574	0.00678	0.84603	0.68203
p32pr	Cortex	-0.00361	0.00678	-0.53207	0.82894
a24	Cortex	0.01665	0.00678	2.45488	0.14565
d32	Cortex	-0.00681	0.00678	-1.00457	0.62756
8BM	Cortex	-0.00346	0.00678	-0.50941	0.83939
p32	Cortex	-0.00165	0.00678	-0.24319	0.90141
10r	Cortex	0.00336	0.00678	0.49576	0.84024
47m	Cortex	-0.00083	0.00678	-0.12227	0.95368
8Av	Cortex	-0.00243	0.00678	-0.35841	0.87126
8Ad	Cortex	-0.01002	0.00678	-1.47770	0.39546
9m	Cortex	-0.00585	0.00678	-0.86247	0.67886
8BL	Cortex	-0.00626	0.00678	-0.92292	0.65042
9p	Cortex	-0.01713	0.00678	-2.52518	0.12728
10d	Cortex	-0.00487	0.00678	-0.71860	0.72773
8C	Cortex	-0.01045	0.00678	-1.54135	0.37947
44	Cortex	-0.01262	0.00678	-1.86137	0.27270
45	Cortex	-0.01284	0.00678	-1.89287	0.27270
47l	Cortex	0.00237	0.00678	0.34882	0.87126
a47r	Cortex	-0.00464	0.00678	-0.68454	0.73266
6r	Cortex	-0.02172	0.00678	-3.20281	0.03641
IFJa	Cortex	-0.00172	0.00678	-0.25339	0.90117
IFJp	Cortex	0.00253	0.00678	0.37325	0.87126
IFSp	Cortex	-0.00485	0.00678	-0.71474	0.72773
IFSa	Cortex	-0.01245	0.00678	-1.83554	0.28237
p9-46v	Cortex	-0.00905	0.00678	-1.33371	0.46702
46	Cortex	-0.01508	0.00678	-2.22323	0.19605
a9-46v	Cortex	-0.00632	0.00678	-0.93132	0.65042
9-46d	Cortex	-0.01794	0.00678	-2.64598	0.10183
9a	Cortex	-0.01536	0.00678	-2.26490	0.18333
10v	Cortex	0.00268	0.00678	0.39465	0.87126
a10p	Cortex	0.00055	0.00678	0.08039	0.96695

10pp	Cortex	0.00357	0.00678	0.52667	0.82894
11l	Cortex	0.01119	0.00678	1.64977	0.34285
13l	Cortex	0.01039	0.00678	1.53163	0.37947
OFC	Cortex	0.01134	0.00678	1.67256	0.34147
47s	Cortex	-0.00137	0.00678	-0.20182	0.91332
LIPd	Cortex	-0.01465	0.00678	-2.16046	0.21296
6a	Cortex	-0.00470	0.00678	-0.69285	0.73266
i6-8	Cortex	-0.00702	0.00678	-1.03519	0.61821
s6-8	Cortex	-0.00866	0.00678	-1.27653	0.49649
43	Cortex	-0.00742	0.00678	-1.09429	0.57537
OP4	Cortex	0.00432	0.00678	0.63644	0.75447
OP1	Cortex	-0.00154	0.00678	-0.22677	0.90514
OP2-3	Cortex	-0.01053	0.00678	-1.55240	0.37947
52	Cortex	-0.01038	0.00678	-1.53088	0.37947
RI	Cortex	-0.01352	0.00678	-1.99418	0.25715
PFcm	Cortex	-0.02033	0.00678	-2.99862	0.06346
PoI2	Cortex	0.00194	0.00678	0.28669	0.88295
TA2	Cortex	0.00071	0.00678	0.10425	0.95798
FOP4	Cortex	-0.01450	0.00678	-2.13747	0.21753
MI	Cortex	0.01268	0.00678	1.86912	0.27270
Pir	Cortex	-0.01391	0.00678	-2.05078	0.24310
AVI	Cortex	0.00376	0.00678	0.55448	0.81445
AAIC	Cortex	-0.00240	0.00678	-0.35393	0.87126
FOP1	Cortex	-0.00254	0.00678	-0.37462	0.87126
FOP3	Cortex	-0.00567	0.00678	-0.83533	0.68602
FOP2	Cortex	-0.00657	0.00678	-0.96802	0.65017
PFt	Cortex	-0.00979	0.00678	-1.44359	0.41550
AIP	Cortex	-0.01223	0.00678	-1.80372	0.29433
EC	Cortex	0.01219	0.00678	1.79667	0.29433
PreS	Cortex	0.00124	0.00678	0.18282	0.91882
H	Cortex	0.01125	0.00678	1.65861	0.34285
ProS	Cortex	-0.01312	0.00678	-1.93387	0.27270
PeEc	Cortex	0.00276	0.00678	0.40748	0.87126
STGa	Cortex	-0.01267	0.00678	-1.86882	0.27270
PBelt	Cortex	-0.00797	0.00678	-1.17498	0.53432
A5	Cortex	-0.00702	0.00678	-1.03466	0.61821
PHA1	Cortex	0.00322	0.00678	0.47505	0.84687
PHA3	Cortex	-0.00128	0.00678	-0.18897	0.91882
STSda	Cortex	-0.00338	0.00678	-0.49877	0.84024
STSdp	Cortex	0.00009	0.00678	0.01343	0.99576
STSvp	Cortex	0.00217	0.00678	0.31982	0.87433
TGd	Cortex	-0.00533	0.00678	-0.78574	0.71410
TE1a	Cortex	0.00512	0.00678	0.75442	0.71410
TE1p	Cortex	0.00003	0.00678	0.00504	0.99598
TE2a	Cortex	0.00757	0.00678	1.11550	0.56238
TF	Cortex	0.01546	0.00678	2.28029	0.18333
TE2p	Cortex	0.00318	0.00678	0.46893	0.84687
PHT	Cortex	0.00512	0.00678	0.75501	0.71410
PH	Cortex	-0.01278	0.00678	-1.88491	0.27270
TPOJ1	Cortex	-0.00947	0.00678	-1.39555	0.44139

TPOJ2	Cortex	-0.01859	0.00678	-2.74176	0.09559
TPOJ3	Cortex	-0.00798	0.00678	-1.17720	0.53432
DVT	Cortex	-0.01614	0.00678	-2.37940	0.16222
PGp	Cortex	-0.00276	0.00678	-0.40620	0.87126
IP2	Cortex	-0.01149	0.00678	-1.69398	0.34147
IP1	Cortex	-0.00852	0.00678	-1.25662	0.50552
IP0	Cortex	-0.00476	0.00678	-0.70218	0.73266
PFop	Cortex	-0.00266	0.00678	-0.39232	0.87126
PF	Cortex	-0.01048	0.00678	-1.54499	0.37947
PFm	Cortex	-0.00611	0.00678	-0.90093	0.65474
PGi	Cortex	-0.01779	0.00678	-2.62335	0.10183
PGs	Cortex	-0.01044	0.00678	-1.53966	0.37947
V6A	Cortex	-0.00489	0.00678	-0.72048	0.72773
VMV1	Cortex	-0.01859	0.00678	-2.74079	0.09559
VMV3	Cortex	0.00487	0.00678	0.71773	0.72773
PHA2	Cortex	-0.00110	0.00678	-0.16258	0.92528
V4t	Cortex	-0.00008	0.00678	-0.01199	0.99576
FST	Cortex	-0.00209	0.00678	-0.30885	0.87433
V3CD	Cortex	-0.00071	0.00678	-0.10422	0.95798
LO3	Cortex	-0.00246	0.00678	-0.36228	0.87126
VMV2	Cortex	0.00040	0.00678	0.05884	0.96959
31pd	Cortex	-0.01485	0.00678	-2.18936	0.20557
31a	Cortex	-0.00116	0.00678	-0.17035	0.92403
VVC	Cortex	0.01280	0.00678	1.88680	0.27270
25	Cortex	0.01133	0.00678	1.66989	0.34147
s32	Cortex	0.01427	0.00678	2.10453	0.22791
pOFC	Cortex	0.00314	0.00678	0.46340	0.84687
PoI1	Cortex	-0.00393	0.00678	-0.57892	0.80317
Ig	Cortex	-0.00656	0.00678	-0.96655	0.65017
FOP5	Cortex	-0.00443	0.00678	-0.65275	0.75081
p10p	Cortex	-0.00551	0.00678	-0.81255	0.69538
p47r	Cortex	-0.01780	0.00678	-2.62540	0.10183
TGv	Cortex	0.00233	0.00678	0.34315	0.87126
MBelt	Cortex	0.00248	0.00678	0.36585	0.87126
LBelt	Cortex	-0.00163	0.00678	-0.24065	0.90141
A4	Cortex	-0.00603	0.00678	-0.88937	0.65946
STSva	Cortex	-0.00177	0.00678	-0.26153	0.89951
TE1m	Cortex	0.01102	0.00678	1.62466	0.35444
PI	Cortex	0.00632	0.00678	0.93178	0.65042
a32pr	Cortex	-0.00142	0.00678	-0.20969	0.91194
p24	Cortex	0.01653	0.00678	2.43747	0.14565

Supplementary References

[1] K. G. M. M. Alberti et al. "Harmonizing the metabolic syndrome: a joint interim statement of the International Diabetes Federation Task Force on Epidemiology and Prevention; National Heart, Lung, and Blood Institute; American Heart Association; World Heart Federation; International Atherosclerosis Society; and International Association for the Study of Obesity". eng. In: *Circulation* 120.16 (Oct. 2009), pp. 1640–1645. ISSN: 1524-4539. DOI: 10.1161/CIRCULATIONAHA.109.192644.

[2] Fidel Alfaro-Almagro et al. "Confound modelling in UK Biobank brain imaging". en. In: *NeuroImage* (June 2020), p. 117002. ISSN: 1053-8119. DOI: 10.1016/j.neuroimage.2020.117002. URL: http://www.sciencedirect.com/science/article/pii/S1053811920304882 (visited on 08/18/2020).

[3] Fidel Alfaro-Almagro et al. "Image processing and Quality Control for the first 10,000 brain imaging datasets from UK Biobank". In: *Neuroimage* 166 (Feb. 2018), pp. 400–424. ISSN: 1053-8119. DOI: 10.1016/j.neuroimage.2017.10.034. URL: https://www.ncbi.nlm.nih.gov/pmc/articles/PMC5770339/ (visited on 07/21/2020).

[4] Leo A. Aroian. "The Probability Function of the Product of Two Normally Distributed Variables". In: *The Annals of Mathematical Statistics* 18.2 (June 1947). Publisher: Institute of Mathematical Statistics, pp. 265–271. ISSN: 0003-4851, 2168-8990. DOI: 10.1214/aoms/1177730442.

[5] Manfred E. Beutel et al. "Childhood adversities and distress - The role of resilience in a representative sample". eng. In: *PloS One* 12.3 (2017), e0173826. ISSN: 1932-6203. DOI: 10.1371/journal.pone.0173826.

[6] C. C. Craig. "On the frequency function of xy". In: *Annals of Mathematical Statistics* 7 (1936), pp. 1–15. DOI: 10.1214/aoms/1177732541.

[7] Katrina A. S. Davis et al. "Mental health in UK Biobank – development, implementation and results from an online questionnaire completed by 157 366 participants: a reanalysis". en. In: *BJPsych Open* 6.2 (Mar. 2020). Publisher: Cambridge University Press. ISSN: 2056-4724. DOI: 10.1192/bjo.2019.100.

[8] Bradley Efron and Robert J Tibshirani. *An introduction to the bootstrap.* CRC press, 1994.

[9] Lloyd T. Elliott et al. "Genome-wide association studies of brain imaging phenotypes in UK Biobank". en. In: *Nature* 562.7726 (Oct. 2018). Number: 7726 Publisher: Nature Publishing Group, pp. 210–216. ISSN: 1476-4687. DOI: 10.1038/s41586-018-0571-7. URL: https://www.nature.com/articles/s41586-018-0571-7 (visited on 06/11/2020).

[10] Bruce Fischl et al. "Whole brain segmentation: automated labeling of neuroanatomical structures in the human brain". eng. In: *Neuron* 33.3 (Jan. 2002), pp. 341–355. ISSN: 0896-6273.

[11] Anna Fry et al. "Comparison of Sociodemographic and Health-Related Characteristics of UK Biobank Participants With Those of the General Population". eng. In: *American Journal of Epidemiology* 186.9 (Nov. 2017), pp. 1026–1034. ISSN: 1476-6256. DOI: 10.1093/aje/kwx246.

[12] Heide Glaesmer et al. "The childhood trauma screener (CTS) - development and validation of cut-off-scores for classificatory diagnostics". ger. In: *Psychiatrische Praxis* 40.4 (May 2013), pp. 220–226. ISSN: 1439-0876. DOI: 10.1055/s-0033-1343116.

[13] Matthew F. Glasser and David C. Van Essen. "Mapping human cortical areas in vivo based on myelin content as revealed by T1- and T2-weighted MRI". eng. In: *The Journal of Neuroscience: The Official Journal of the Society for Neuroscience* 31.32 (Aug. 2011), pp. 11597–11616. ISSN: 1529-2401. DOI: 10.1523/JNEUROSCI.2180-11.2011.

[14] Matthew F. Glasser et al. "A multi-modal parcellation of human cerebral cortex". en. In: *Nature* 536.7615 (Aug. 2016). Number: 7615 Publisher: Nature Publishing Group, pp. 171–178. ISSN: 1476-4687. DOI: 10.1038/nature18933. URL: https://www.nature.com/articles/nature18933 (visited on 07/15/2020).

[15] Matthew F. Glasser et al. "The minimal preprocessing pipelines for the Human Connectome Project". eng. In: *NeuroImage* 80 (Oct. 2013), pp. 105–124. ISSN: 1095-9572. DOI: 10.1016/j.neuroimage.2013.04.127.

[16] Hans Jörgen Grabe et al. "A brief instrument for the assessment of childhood abuse and neglect: the childhood trauma screener (CTS)". ger. In: *Psychiatrische Praxis* 39.3 (Apr. 2012), pp. 109–115. ISSN: 1439-0876. DOI: 10.1055/s-0031-1298984.

[17] Thomas E. Kraynak et al. "Retrospectively reported childhood physical abuse, systemic inflammation, and resting corticolimbic connectivity in midlife adults". en. In: *Brain, Behavior, and Immunity* 82 (Nov. 2019), pp. 203–213. ISSN: 08891591. DOI: 10.1016/j.bbi.2019.08.186. URL: https://linkinghub.elsevier.com/retrieve/pii/S0889159119303071 (visited on 10/31/2019).

[18] Heidi Lindroth et al. "Examining the identification of age-related atrophy between T1 and T1 + T2-FLAIR cortical thickness measurements". en. In: *Scientific Reports* 9.1 (Aug. 2019). Number: 1 Publisher: Nature Publishing Group, p. 11288. ISSN: 2045-2322. DOI: 10.1038/s41598-019-47294-2. URL: https://www.nature.com/articles/s41598-019-47294-2 (visited on 03/13/2023).

[19] Mary-Ellen Lynall et al. "Peripheral Blood Cell–Stratified Subgroups of Inflamed Depression". English. In: *Biological Psychiatry* 0.0 (Dec. 2019). ISSN: 0006-3223, 1873-2402. DOI: 10.1016/j.biopsych.2019.11.017. URL: https://www.biologicalpsychiatryjournal.com/article/S0006-3223(19)31886-4/abstract (visited on 03/03/2020).

[20] Karla L. Miller et al. "Multimodal population brain imaging in the UK Biobank prospective epidemiological study". en. In: *Nature Neuroscience* 19.11 (Nov. 2016). Number: 11 Publisher: Nature Publishing Group, pp. 1523–1536. ISSN: 1546-1726. DOI: 10.1038/nn.4393. URL: https://www.nature.com/articles/nn.4393 (visited on 07/15/2020).

[21] Gwenllian Moody et al. "Establishing the international prevalence of self-reported child maltreatment: a systematic review by maltreatment type and gender". In: *BMC Public Health* 18.1 (Oct. 2018), p. 1164. ISSN: 1471-2458. DOI: 10.1186/s12889-018-6044-y. URL: https://doi.org/10.1186/s12889-018-6044-y (visited on 08/19/2020).

[22] Athanasia Mo Mowinckel and Didac Vidal-Piñeiro. *ggsegGlasser: Glasser datasets for the ggseg-plotting tool.* 2020. URL: https://github.com/LCBC-UiO/ggsegGlasser.

[23] Alejo J Nevado-Holgado et al. "Commonly prescribed drugs associate with cognitive function: a cross-sectional study in UK Biobank". In: *BMJ Open* 6.11 (Nov. 2016). ISSN: 2044-6055. DOI: 10.1136/bmjopen-2016-012177. URL: https://www.ncbi.nlm.nih.gov/pmc/articles/PMC5168501/ (visited on 04/06/2020).

[24] Kristopher J. Preacher and Andrew F. Hayes. "Asymptotic and resampling strategies for assessing and comparing indirect effects in multiple mediator models". en. In: *Behavior Research Methods* 40.3 (Aug. 2008), pp. 879–891. ISSN: 1554-3528. DOI: 10.3758/BRM.40.3.879. URL: https://doi.org/10.3758/BRM.40.3.879 (visited on 07/09/2020).

[25] Kristopher J. Preacher and James P. Selig. "Advantages of Monte Carlo Confidence Intervals for Indirect Effects". In: *Communication Methods and Measures* 6.2 (Apr. 2012). Publisher: Routledge _eprint: https://doi.org/10.1080/19312458.2012.679848, pp. 77–98. ISSN: 1931-2458. DOI: 10.1080/19312458.2012.679848. URL: https://doi.org/10.1080/19312458.2012.679848 (visited on 11/02/2021).

[26] Martin Reuter et al. "Head Motion during MRI Acquisition Reduces Gray Matter Volume and Thickness Estimates". In: *NeuroImage* 107 (Feb. 2015), pp. 107–115. ISSN: 1053-8119. DOI: 10.1016/j.neuroimage.2014.12.006. URL: https://www.ncbi.nlm.nih.gov/pmc/articles/PMC4300248/ (visited on 08/18/2020).

[27] Adon F. G. Rosen et al. "Quantitative assessment of structural image quality". eng. In: *NeuroImage* 169 (2018), pp. 407–418. ISSN: 1095-9572. DOI: 10.1016/j.neuroimage.2017.12.059.

[28] Yves Rosseel. “lavaan: An R Package for Structural Equation Modeling”. In: *Journal of Statistical Software* 48.2 (2012), pp. 1–36. URL: http://www.jstatsoft.org/v48/i02/.

[29] Rita Santos et al. “A comprehensive map of molecular drug targets”. eng. In: *Nature Reviews. Drug Discovery* 16.1 (2017), pp. 19–34. ISSN: 1474-1784. DOI: 10.1038/nrd.2016.230.

[30] Neil K. Savalia et al. “Motion-related artifacts in structural brain images revealed with independent estimates of in-scanner head motion”. eng. In: *Human Brain Mapping* 38.1 (2017), pp. 472–492. ISSN: 1097-0193. DOI: 10.1002/hbm.23397.

[31] Karin Schermelleh-Engel, Helfried Moosbrugger, Hans Müller, et al. “Evaluating the fit of structural equation models: Tests of significance and descriptive goodness-of-fit measures”. In: *Methods of psychological research online* 8.2 (2003). Publisher: Citeseer, pp. 23–74.

[32] Michael E Sobel. “Asymptotic confidence intervals for indirect effects in structural equation models”. In: *Sociological methodology* 13 (1982). Publisher: JSTOR, pp. 290–312.

[33] Eva-Maria Stauffer et al. “Grey and white matter microstructure is associated with polygenic risk for schizophrenia”. en. In: *Molecular Psychiatry* (Aug. 2021), pp. 1–10. ISSN: 1476-5578. DOI: 10.1038/s41380-021-01260-5. URL: https://www.nature.com/articles/s41380-021-01260-5 (visited on 12/13/2021).

[34] Anne Tüngler et al. “Body mass index but not genetic risk is longitudinally associated with altered structural brain parameters”. en. In: *Scientific Reports* 11.1 (Dec. 2021). Number: 1 Publisher: Nature Publishing Group, p. 24246. ISSN: 2045-2322. DOI: 10.1038/s41598-021-03343-3. URL: https://www.nature.com/articles/s41598-021-03343-3 (visited on 01/25/2023).

[35] Hadley Wickham. *ggplot2: Elegant Graphics for Data Analysis.* Springer-Verlag New York, 2016. ISBN: 978-3-319-24277-4. URL: https://ggplot2.tidyverse.org.

[36] Hadley Wickham et al. *dplyr: A Grammar of Data Manipulation.* 2020. URL: https://CRAN.R-project.org/package=dplyr.

[37] Hadley Wickham et al. “Welcome to the Tidyverse”. en. In: *Journal of Open Source Software* 4.43 (Nov. 2019), p. 1686. ISSN: 2475-9066. DOI: 10.21105/joss.01686. URL: https://joss.theoj.org/papers/10.21105/joss.01686 (visited on 02/03/2023).

[38] Andreas Witt et al. “Validation and standardization of the Childhood Trauma Screener (CTS) in the general population”. In: *Child and Adolescent Psychiatry and Mental Health* 16.1 (Sept. 2022), p. 73. ISSN: 1753-2000. DOI: 10.1186/s13034-022-00506-6. URL: https://doi.org/10.1186/s13034-022-00506-6 (visited on 10/27/2023).

[39] Yeda Wu et al. “Genome-wide association study of medication-use and associated disease in the UK Biobank”. en. In: *Nature Communications* 10.1 (Apr. 2019). Number: 1 Publisher: Nature Publishing Group, pp. 1–10. ISSN: 2041-1723. DOI: 10.1038/s41467-019-09572-5. URL: https://www.nature.com/articles/s41467-019-09572-5 (visited on 04/06/2020).
